# Central Adiposity Increases Risk of Kidney Stone Disease through Effects on Serum Calcium Concentrations

**DOI:** 10.1681/ASN.0000000000000238

**Published:** 2023-10-03

**Authors:** Catherine E. Lovegrove, Jelena Bešević, Akira Wiberg, Ben Lacey, Thomas J. Littlejohns, Naomi E. Allen, Michelle Goldsworthy, Jihye Kim, Fadil M. Hannan, Gary C. Curhan, Ben W. Turney, Mark I. McCarthy, Anubha Mahajan, Rajesh V. Thakker, Michael V. Holmes, Dominic Furniss, Sarah A. Howles

**Affiliations:** 1 Nuffield Department of Surgical Sciences, University of Oxford, Oxford, United Kingdom; 2 Nuffield Department of Population Health, University of Oxford, Oxford, United Kingdom; 3 Nuffield Department of Orthopaedics, Rheumatology and Musculoskeletal Sciences, University of Oxford, Oxford, United Kingdom; 4 Harvard T.H. Chan School of Public Health, Boston, Massachusetts; 5 Nuffield Department of Women's and Reproductive Health, University of Oxford, Oxford, United Kingdom; 6 Channing Division of Network Medicine and Renal Division, Brigham and Women's Hospital, Harvard Medical School, Boston, Massachusetts; 7 Wellcome Centre for Human Genetics, Nuffield Department of Medicine, University of Oxford, Oxford, United Kingdom; 8 Genentech, South San Francisco, Califirnia; 9 Academic Endocrine Unit, Radcliffe Department of Medicine, University of Oxford, Oxford, United Kingdom; 10 Medical Research Council, Integrative Epidemiology Unit, University of Bristol, Bristol, United Kingdom

**Keywords:** calcium, diabetes, human genetics, kidney stones, obesity

## Abstract

**Significance Statement:**

Kidney stone disease is a common disorder with poorly understood pathophysiology. Observational and genetic studies indicate that adiposity is associated with an increased risk of kidney stone disease. However, the relative contribution of general and central adipose depots and the mechanisms by which effects of adiposity on kidney stone disease are mediated have not been defined. Using conventional and genetic epidemiological techniques, we demonstrate that general and central adiposity are independently associated with kidney stone disease. In addition, one mechanism by which central adiposity increases risk of kidney stone disease is by increasing serum calcium concentration. Therapies targeting adipose depots may affect calcium homeostasis and help to prevent kidney stone disease.

**Background:**

Kidney stone disease affects approximately 10% of individuals in their lifetime and is frequently recurrent. The disease is linked to obesity, but the mechanisms mediating this association are uncertain.

**Methods:**

Associations of adiposity and incident kidney stone disease were assessed in the UK Biobank over a mean of 11.6 years/person. Genome-wide association studies and Mendelian randomization (MR) analyses were undertaken in the UK Biobank, FinnGen, and in meta-analyzed cohorts to identify factors that affect kidney stone disease risk.

**Results:**

Observational analyses on UK Biobank data demonstrated that increasing central and general adiposity is independently associated with incident kidney stone formation. Multivariable MR, using meta-analyzed UK Biobank and FinnGen data, established that risk of kidney stone disease increases by approximately 21% per one standard deviation increase in body mass index (BMI, a marker of general adiposity) independent of waist-to-hip ratio (WHR, a marker of central adiposity) and approximately 24% per one standard deviation increase of WHR independent of BMI. Genetic analyses indicate that higher WHR, but not higher BMI, increases risk of kidney stone disease by elevating adjusted serum calcium concentrations (β=0.12 mmol/L); WHR mediates 12%–15% of its effect on kidney stone risk in this way.

**Conclusions:**

Our study indicates that visceral adipose depots elevate serum calcium concentrations, resulting in increased risk of kidney stone disease. These findings highlight the importance of weight loss in individuals with recurrent kidney stones and suggest that therapies targeting adipose depots may affect calcium homeostasis and contribute to prevention of kidney stone disease.

## Visual Abstract

**Figure:**
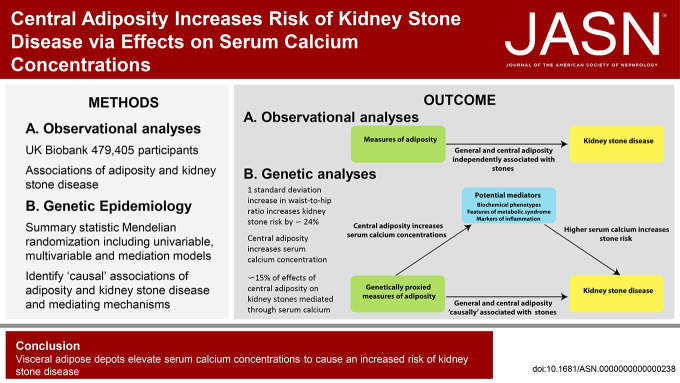

## Introduction

Kidney stone disease is a common pathology, affecting up to 20% of men and 10% of women by age 70 years with a recurrence rate of approximately 50% at 5 years.^1,2^ Our understanding of the pathophysiological processes underlying kidney stone formation is incomplete, preventing effective prophylaxis in many cases.^2^

Observational studies indicate that general adiposity (body mass index [BMI]) and central adiposity (waist–hip ratio [WHR] and waist circumference [WC]) are associated with an increased risk of kidney stone disease.^3^ Metabolic syndrome includes central obesity, hypertension, dyslipidemia, and impaired glucose tolerance, all of which have been postulated to increase risk of kidney stone formation.^4,5^ The mechanisms linking obesity and metabolic syndrome with an increased risk of kidney stone disease are uncertain but may include hyperuricosuria and hyperoxaluria, hyperinsulinaemia resulting in hypercalciuria, insulin resistance causing impaired renal ammonium generation and hypocitraturia, hypertension altering the urinary lithogenic profile, and vascular insult modifying renal papillary circulation.^3,6–8^ Increasing adiposity has also been linked to alterations in serum concentrations of calcium, phosphate, vitamin D, and urate, all of which may affect risk of kidney stone disease.^9,10^ Furthermore, obesity increases serum markers of systemic inflammation, and inflammation has been postulated to increase risk of kidney stone disease.^11^

Conventional epidemiologic studies may be subject to bias, particularly from reverse causality and confounding.^12^ Mendelian randomization (MR) is a genetic epidemiological technique that aims to overcome these problems, using genetic variants associated with an exposure to reduce bias in identifying causal effects and their magnitude and allow direction of effect to be established.^12,13^ Furthermore, multivariable and mediation MR facilitate the identification of independent causal effects and estimation of the relative importance of multiple exposures.^12,13^ Recent studies have used MR techniques to increase understanding of the pathogenesis of kidney stone disease and its relation to adiposity and metabolic syndrome^14,15^; however, the relative contributions of central and general adiposity have not been assessed using multivariable techniques, nor have mediators of effects of adiposity on risk of kidney stone disease been identified. In this study, we use both conventional and genetic epidemiological approaches to demonstrate that increased central adiposity is causally associated with kidney stone disease, independent of general adiposity, and that the influence of central adiposity on kidney stone risk is due, in part, to increasing serum calcium concentrations. In contrast to previous smaller studies, we find no evidence that other components of the metabolic syndrome, serum uric acid levels, or biochemical markers of inflammation are causally associated with nephrolithiasis.^14,15^

## Methods

### Study Participants

The UK Biobank recruited 502,000 individuals aged 40–69 years from 2006 to 2010. Participants provided health-related questionnaires, physical measurements, and blood samples and consented to linkage of data to medical records.^16^ UK Biobank is approved by the National Information Governance Board for Health and Social Care and the National Health Service North West Centre for Research Ethics Committee (Ref: 11/NW/0382). Kidney stone cases were identified using International Classification of Diseases (ICD) revisions 9 and 10, Office of Population Censuses and Surveys Classification of Surgical Operations and Procedures (OPCS) revisions 3 and 4, and self-report codes (Supplementary Tables 1 and 2).

FinnGen (https://www.finngen.fi/en) is a prospective study combining data from Finnish biobanks and digital health record data from Finnish health registries using personal identification numbers. The Coordinating Ethics Committee of the Helsinki and Uusimaa Hospital District has evaluated the project.^17^ FinnGen release R8 comprises genetic data for 342,499 individuals (190,879 female participants and 151,620 male participants), 20,175,454 genetic variants, and 2202 disease end points in individuals of Finnish ancestry.^17^ Kidney stone cases were identified using ICD-8, -9, and -10 codes for calculus of kidney and ureter.^18^

### Observational Analyses

Observational analyses were undertaken in the UK Biobank, excluding participants with missing or extreme (top/bottom 0.001%) values of anthropometric measurements, prevalent kidney stone disease, or conditions predisposing to kidney stone formation (Supplementary Table 3). Participants were censored at earliest diagnosis of kidney stone disease, death, loss to follow-up, or February 28, 2018 (Wales) or March 31, 2021 (England and Scotland).

**Figure 1:**
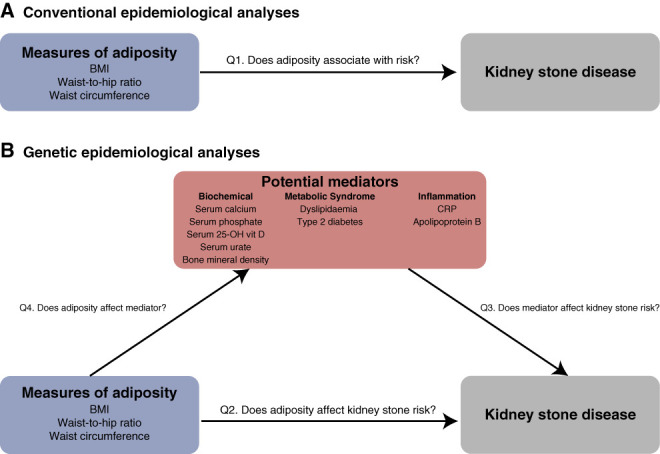
**Study design.** Analyses undertaken to explore associations of adiposity and kidney stone disease. (A) depicts observational analyses in the UK Biobank using Cox proportional hazards regression models to address question 1 (Q1): Does adiposity associate with increased risk of kidney stones? (B) depicts genetic epidemiological analyses using univariable and multivariable Mendelian randomization techniques to address questions 2 (Q2): Does adiposity affect kidney stone risk? and 3 (Q3): Do potential mediators of effects of adiposity on kidney stone risk influence risk of kidney stone disease? Where a potential mediator (X) of effects of adiposity on kidney stone risk was found have effects on kidney stone disease, genetic analyses were undertaken to answer question 4 (Q4): Does adiposity have effects on X? Kidney stone outcomes were based on kidney stone genome-wide association studies (GWAS) summary statistics from the UK Biobank, FinnGen, and a meta-analysis of these two GWAS datasets.

Hazard ratios (HRs) for associations of general adiposity (BMI) and central adiposity (WHR and WC) with incident kidney stone disease were estimated using Cox proportional hazards regression models (Figure 1). Models were stratified by age at risk (in 5-year groups) and ethnicity (White, Other). Given the association of deprivation with obesity and kidney stone disease,^19,20^ we adjusted models for the Townsend Deprivation Index (quintiles) as well as smoking (never, former, current), alcohol drinking (never, former, occasional, at least weekly), and, where appropriate, sex. Associations were corrected for regression dilution using correlations between resurvey and baseline measurements (*i.e.* to estimate associations with long-term average levels); hence, the values reported in the observational analysis represent the association with the average usual levels of the anthropometric measure.^21^

Anthropometric measures were categorized into fifths separately in men and women, with HRs reported relative to the lowest category. Confidence intervals (CIs) were calculated using variance of the log risk.^22^ Linear associations (reported per 5 kg/m^2^, 0.05, and 10 cm higher BMI, WHR, and WC, respectively) were used to facilitate comparison of estimates between male and female subsets. To assess the relative independence of anthropometric measures, associations with BMI were adjusted for WHR and WC and associations with WHR and WC by BMI. χ^2^ values were derived from likelihood ratio statistics.^23^ This statistic provides a significance test for the improvement in fit from including the main adiposity term and a quantitative measure of the extent to which the adiposity term improves risk prediction in different models (*e.g.* with and without adjustment of other adiposity terms).

The effect of WHR on albumin-adjusted serum calcium concentrations in male participants and female participants was evaluated in a linear regression model adjusted for age. Analyses were performed using Rv4.1.1.

### Genome-wide Association Studies and Meta-analysis

Genome-wide association studies (GWAS) were performed in the UK Biobank, excluding participants with conditions predisposing to kidney stone disease (Supplementary Table 3). Genotyping was undertaken using UK-BiLEVE and UK-Biobank Axiom Arrays and called using array intensity data and a custom genotype-calling pipeline.^24^ PLINKv1.9 and Rv3.6.1 were used for quality control (QC). Sample-, individual-, and SNP-level QC exclusions are shown in Supplementary Methods.

UK Biobank phasing on autosomes was performed with SHAPEIT3 using the 1000 Genomes phase 3 dataset as a reference panel. The Haplotype Reference Consortium reference panel and a merged UK10K/1000 Genomes Phase 3 panel were used in imputation. The resultant dataset comprised 92,693,895 autosomal SNPs, short indels, and large structural variants.^24^

A total of 547,011 genotyped and 8,397,548 imputed autosomal SNPs and 733,758 genotyped and 2,635,881 X-chromosome SNPs with MAF ≥0.01 and Info Score ≥0.9 were used at GWAS, using a linear mixed noninfinitesimal model implemented in BOLT-LMMv2.3.^25^ BOLT-LMM accounts for genetic relationships between individuals. The hg19 reference genetic map and reference linkage disequilibrium (LD) score file for European ancestry were used. Genotyping platform and sex were incorporated as covariates. In X-chromosome analyses, multiallelic SNPs, SNPs of differential missingness between male participants and female participants (*P*<5×10^−7^), or Hardy–Weinberg equilibrium *P*<1×10^−6^ in female participants were excluded. Male genotype was specified as 0/2 and female genotype as 0/1/2 corresponding to a model of random X inactivation. Pseudoautosomal and nonpseudoautosomal SNPs were merged.^25^ Quantile–quantile and Manhattan plots were generated in FUMA.^26^ Conditional analyses were performed using QCTOOLv2.

A fixed-effects meta-analysis of kidney stone disease was undertaken using UK Biobank and FinnGen kidney stone GWAS summary statistics for autosomes and the X-chromosome.^17,18^ FinnGen r8 GWAS data are publicly available for the phenotype N14 calculus of kidney and ureter comprising 8597 cases and 333,128 controls.^17,18^ Information on sample phenotyping, genotyping, and GWAS in the FinnGen sample has been previously described.^17^ SNPs with MAF <0.01 were omitted from FinnGen summary statistics. The FinnGen study GWAS summary statistics do not report sex-specific datasets, precluding sex-stratified analyses. Meta-analysis was undertaken in METAL using inverse-weighting of log odds ratios.^27^ SNPs with a high level of heterogeneity between studies (I^2^ statistic >75%) were excluded. The resultant summary statistics were used to perform MR analyses.

### Multiple Association Signals at Kidney Stone Disease Risk Loci (Genome-Wide Complex Trait Analysis)

A GWAS locus was defined as a chromosomal region with adjacent pairs of kidney stone disease-associated SNPs <1 Mb apart.^28,29^ To identify the presence of distinct association signals at genome-wide significant loci, Genome-Wide Complex Trait Analysis (GCTA) software version 1.94.1 was used to perform step-wise approximate conditional and joint analysis with the same UK Biobank LD reference panel as was used in the UK Biobank kidney stone disease GWAS.^28,30^ Where there was a single signal of association at a locus, we defined the index SNP as the lead SNP from unconditional meta-analysis. For loci with multiple association signals, we defined the index SNP as that with the lowest *P* value in conditional approximate analysis.

### Heritability of Kidney Stone Disease, Polygenicity, and Population Stratification

Estimates of genomic inflation and the heritability of kidney stone disease in each cohort and subsequent meta-analysis were obtained using LD score regression (LDSC) v1.0.1.^27,28^ Analyses were restricted to variants present in HapMap3 (https://www.sanger.ac.uk/resources/downloads/human/hapmap3.html) and LD Scores computed using 1000 Genomes European data (https://data.broadinstitute.org/alkesgroup/LDSCORE/eur_w_ld_chr.tar.bz2).^29^ For liability transformations, a population prevalence approximation of 10% was used. To assess similarity of genetic effects between cohorts, the cross-trait LD Score Regression (LDSC) v1.0.1 was used to calculate pairwise genetic correlations (r_*g*_) on the basis of summary statistics from each cohort for variants present in HapMap3.^27,28^

### Gene and Gene Set-Based Analyses

To identify genes and gene sets associated with kidney stone disease on the basis of effect estimates from meta-analysis, MAGMA v1.10^31^ was used. MAGMA summarizes variant-level *P*-values according to gene positions and LD structure. Variants were mapped to 18,143 genes based on rsID. For each gene, associations with kidney stone disease were determined using a SNP-wise mean model, where LD patterns were calculated using ancestry appropriate 1 KGP reference genotypes. Statistical significance of associations with kidney stone disease was defined using a Bonferroni-corrected threshold of *P*=0.05/18,143=2.76×10^−6^. To identify biological pathways or cell types implicated in kidney stone disease, gene-based test statistics were used to perform a competitive set-based analysis of 15,685 Human Phenotype Ontology (HPO) GO term-based gene sets downloaded from the Molecular Signature Database v2022.1 (https://www.gsea-msigdb.org/gsea/msigdb/collections.jsp).^31–33^ Bonferroni-corrected *P*-value thresholds were used to identify HPO gene sets and genes within each gene set showing significant overlap with kidney stone meta-analysis summary statistics (*P*=0.05/18,685=2.68×10-6 and *P*=0.05/number of genes in gene set, respectively). Functional Mapping and Annotation of genetic associations (FUMA) v1.3.0 (https://fuma.ctglab.nl/) Gene2Func module was used to identify differential gene expression in GTEx v8 kidney cortex tissue.^26^

### Mendelian Randomization

MR analyses use genetic instrumental variables (IVs) to interrogate causal effects of an exposure on an outcome.^34^ Three key assumptions underlie the principles of MR: that IVs are associated with the risk factor of interest (relevance), that IVs only affect the outcome through their effect on the exposure variable (exclusion restriction); and that there are no unmeasured confounders of the associations between IVs and the outcome (independence).^34,35^

MR analyses were performed for IVW, MR-Egger, weighted median, contamination mixture, and multivariable analyses using MendelianRandomization and TwoSampleMR in R.^12,36,37^ SNPs with independent, GWAS-significant (*P*<5×10^−8^) associations with phenotypes of interest in individuals of European ancestry were selected as IVs from relevant studies for summary statistic Mendelian randomization (Supplementary Table 4). For increased stringency, and to minimize risk of type-1 error from IVs being in linkage disequilibrium, further pruning of IVs was undertaken using the *clump_data()* function with clump-r^2^ set at 0.01 using a European population reference panel. Genetically proxied measures of adiposity (BMI,^38^ WHR,^38^ WC,^38^ and visceral, abdominal subcutaneous, and gluteofemoral fat depots^39^) were identified from relevant studies to facilitate MR studies to ascertain whether adiposity increases kidney stone risk (Figure 1).

To investigate the mechanisms by which general and central adiposity influence risk of kidney stone disease, investigations were undertaken in two steps: First, MR was used to determine whether risk factors for kidney stone disease identified by conventional epidemiologic studies had causal associations with kidney stone disease; subsequently, where causal effects on kidney stones were identified, MR was used to assess the effects of adiposity on these kidney stone risk factors (Figure 1). Genetic proxies of risk factors for kidney stone disease that were considered included features of the metabolic syndrome (type 2 diabetes,^40^ 2-hour glucose tolerance,^41^ fasting glucose,^41^ fasting insulin,^41^ HbA1c,^41^ HDL cholesterol,^42^ LDL cholesterol,^42^ triglyceride concentrations,^42^ and systolic and diastolic bloods pressure^43^), serum and urinary biochemical phenotypes (24-hour urinary calcium [personal communication with G. Curhan], serum 25-hydroxy vitamin D, albumin-adjusted calcium,^44^ phosphate,^44^ and urate concentrations^45^), heel bone mineral density,^46^ and markers of inflammation (serum apolipoprotein-B^42^ and C-reactive protein concentrations^47^) (Supplementary Tables 4 and 5). Outcome IVs for kidney stone disease were derived from summary statistics generated by UK Biobank and FinnGen GWAS for kidney stone disease, and meta-analysis, described above. Mean and SD of IV R^2^ were reported to evaluate the proportion of variability explained by the IVs (Supplementary Table 4).

Exposure and outcome data were harmonized for exposure phenotypes with three or more associated, significant, independent SNPs, and allele frequencies were used to infer positive strand alleles for palindromic IVs. Where harmonization was not possible and the positive strand alleles remained ambiguous, IVs were omitted from analysis. MR-Egger and inverse-variance weighted (IVW) analyses were undertaken for all exposure–outcome pairs. Where MR-Egger regression intercept estimate was zero (*P*>0.05), IVW was interpreted as estimate of best fit. Where MR-Egger intercept estimate suggested potential horizontal pleiotropy (*P*<0.05), MR-Egger regression was interpreted as estimate of best fit.^48^ In instances where there was ambiguity surrounding results, further estimates were considered, including weighted median and contamination mix methods. All IVW *P*-values were adjusted for multiple testing using the Benjamini–Hochberg false discovery rate method, controlled at 5%, to account for exploring the relationships of numerous obesity and metabolic traits with kidney stone disease.^49^ To investigate reverse causality, bidirectional MR analyses were performed. Where significant exposure–outcome relationships were identified on univariable MR or where the Steiger test for directionality demonstrated that IVs explained greater variance in the exposure than the outcome, Steiger filtering was undertaken as a sensitivity analysis.^50^ Mediation analyses were performed to identify direct and indirect effects as previously reported.^12^ In brief, the direct effect of an exposure on an outcome (c′) and on a mediating variable (A) are estimated, as is the effect of a mediating variable on the outcome (B). The indirect effect of a mediating variable on the outcome is the product of the effect of the exposure on the mediator and the mediator on the outcome (A × B). The proportion mediated effect is the quotient of the indirect and the total effect (Supplementary Figure 1).

To assess the plausibility of the core IV assumptions, mean F statistics and total *R*^*2*^ across SNPs used as exposure IVs were calculated using the following formulas where the genetic association with the risk factor (â) is in standard deviation units, *MAF* is the minor allele frequency, *N* is the sample size for the IV–outcome association, and *K* is the number of IVs (Supplementary Table 4)^51–53^:

$$R^{2}=2{â}^{2}x\;MAF\;x\;\left(1-MAF\right)$$

$$F=\frac{R^{2}\left(N-1-K\right)}{\left(1-R^{2}\right)K}$$

F statistics ranged from 0.01 (serum phosphate concentration) to 62.41 (systolic blood pressure), and mean *R*^*2*^ ranged from 2.76×10^−6^, SD=9.69×10^−7^ (serum phosphate and UK Biobank kidney stone disease), to 0.035, SD=0.034 (systolic blood pressure) (Supplementary Table 4). Probability of weak instrument bias and type 1 error from participant overlap between study cohorts for BMI, WHR, and kidney stone disease in UK Biobank were calculated as 0.001 and 0.05, respectively, using https://sb452.shinyapps.io/overlap/.^51^ Multivariable MR was performed to control for the effect of IVs on possible confounding variables; the Cochrane Q test was used to identify heterogeneity in causal estimates. Analyses omitting one IV, leave-one-out analyses, were undertaken where significant effects were identified from univariable MR estimates. Our study is reported in accordance with the STROBE-MR guidelines.^54,55^

### Ethical Approval

This research was conducted using the UK Biobank Resource under application number 885. UK Biobank is approved by the National Information Governance Board for Health and Social Care and the National Health Service North West Centre for Research Ethics Committee (Ref:11/NW/0382). The Coordinating Ethics Committee of the Helsinki and Uusimaa Hospital District has evaluated the FinnGen study.

## Results

### Associations of Adiposity and Kidney Stone Disease

Among 479,405 participants included in observational analyses, mean age at baseline was 56.5 years (SD=8.1); 55% were female (Table 1, Supplementary Figure 2).

**Table 1:** Baseline characteristics of the participants in observational studies of UK Biobank

Characteristic	Men (*n*=216,157)	Women (*n*=263,248)	Overall (*n*=479,405)
Demographic and lifestyle factors			
Age, mean (SD)	56.7 (8.2)	56.3 (8.0)	56.5 (8.1)
White, *n* (%)	204,727 (94.7)	249,298 (94.7)	454,025 (94.7)
Current smoker, *n* (%)	26,863 (12.4)	23,288 (8.8)	50,151 (10.5)
Alcohol drinker (at least weekly), *n* (%)	168,274 (77.8)	165,048 (62.7)	333,322 (69.5)
Townsend Deprivation Index, mean (SD)	−1.3 (3.1)	−1.4 (3.0)	−1.3 (3.1)
Anthropometric measures, mean (SD)			
Body mass index, kg/m^2^	27.8 (4.2)	27.1 (5.1)	27.4 (4.8)
Waist circumference (cm)	96.8 (11.2)	84.6 (12.4)	90.1 (13.4)
Waist-to-hip ratio	0.94 (0.06)	0.82 (0.07)	0.87 (0.09)
Kidney stone disease status			
Case, *n* (%)	3146 (1.5)	1830 (0.7)	4976 (1.0)

Participants were mainly of White ethnicity (95%) and from less-deprived areas than the national average (mean Townsend Deprivation Index score −1.33, SD=3.1). At baseline, mean BMI, WHR, and WC were 27.8 kg/m^2^ (SD=4.8), 0.94 (SD=0.06), and 96.8 cm (SD=11.2), respectively, among men, and 27.1 kg/m^2^ (SD=5.1), 0.82 (SD=0.07), and 84.6 cm (SD=12.4), respectively, among women. Self-correlations between baseline and resurvey measurements were 0.93 for BMI, 0.67 for WHR, and 0.82 for WC in men and 0.92 for BMI, 0.66 for WHR, and 0.83 for WC in women.

**Figure 2:**
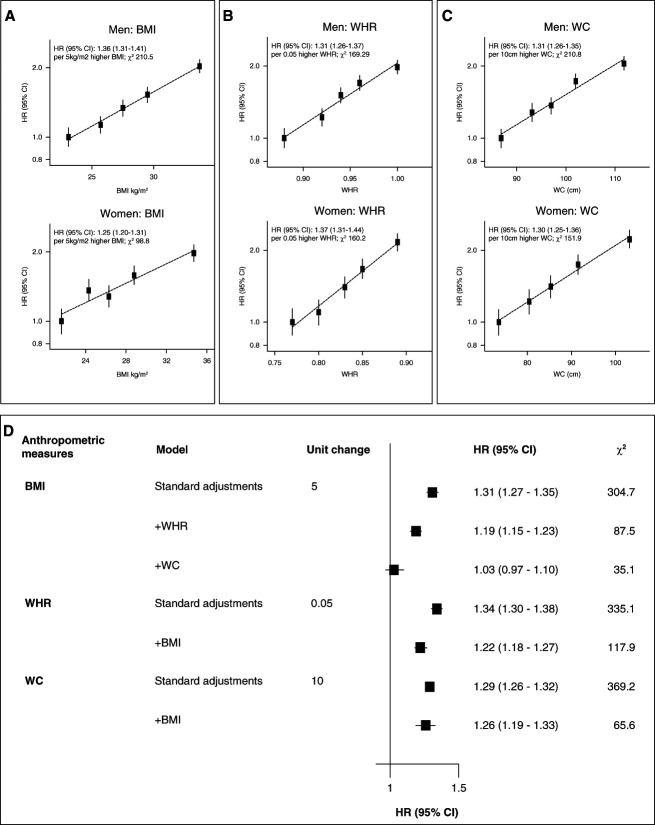
**Incident kidney stones versus anthropometric measures in observational analyses in the UK Biobank.** Hazard ratios (HR) stratified by age at risk and ethnicity for incident kidney stones versus (A) body mass index (BMI), (B) waist-to-hip ratio (WHR), and (C) waist circumference (WC) among 479,405 participants. (D) HR of anthropometric measures with additional adjustment for each other in combined-sex analyses. Analyses are adjusted for Townsend Deprivation Index, smoking, and alcohol drinking and exclude participants with preexisting kidney stones (or conditions known to predispose to kidney stones) at baseline and those with missing or outlying values in anthropometric variables or key covariates. The variance of the category−specific log risk determines the confidence interval (CI).

During 5.6 million person-years of follow-up (mean 11.6 years/person), 4976 individuals developed incident kidney stone disease. In both sexes, each adiposity measure was strongly positively associated with incident kidney stone disease, with overlapping 95% CIs, and no evidence of a threshold effect (Figure 2). In analyses combining both sexes, a 5 kg/m^2^ higher BMI was associated with an approximately 30% higher risk of incident kidney stone disease (hazard ratio [HR]=1.31, 95% CI=1.27–1.35), as was a 0.05 higher WHR (HR=1.34, 95% CI=1.30–1.38) and a 10 cm larger WC (HR=1.29, 95% CI=1.26–1.32) (Figure 2, Table 2). In sex-stratified analyses, measures of central adiposity (WHR and WC) conferred a higher risk of incident kidney stone disease than measures of general adiposity (BMI); however, there was overlap of 95% CIs (Supplementary Figure 3).

**Table 2:** Incident kidney stones versus anthropometric measures in UK Biobank (quintile analysis)

Men				Women			
Exposure	Quintile	*N*	HR (95% CI)	Exposure	*N*	Quintile	HR (95% CI)
BMI	Q1	449/42,910	REF	BMI	248/53,833	Q1	REF
	Q2	519/44,051	1.13 (1.00 to 1.29)		322/51,261	Q2	1.36 (1.15 to 1.60)
	Q3	603/43,154	1.34 (1.18 to 1.51)		320/52,273	Q3	1.28 (1.09 to 1.52)
	Q4	662/41,252	1.53 (1.35 to 1.72)		414/52,868	Q4	1.58 (1.35 to 1.85)
	Q5	913/41,644	2.03 (1.81 to 2.28)		526/51,183	Q5	1.97 (1.69 to 2.30)
WHR	Q1	420/42,575	REF	WHR	234/52,856	Q1	REF
	Q2	522/43,410	1.22 (1.07 to 1.39)		259/51,980	Q2	1.10 (0.92 to 1.31)
	Q3	628/41,857	1.51 (1.33 to 1.71)		341/52,014	Q3	1.40 (1.18 to 1.65)
	Q4	724/42,684	1.70 (1.51 to 1.92)		428/52,878	Q4	1.67 (1.42 to 1.97)
	Q5	852/42,485	1.98 (1.76 to 2.23)		568/51,690	Q5	2.16 (1.85 to 2.53)
WC	Q1	479/48,430	REF	WC	252/57,631	Q1	REF
	Q2	487/38,497	1.28 (1.13 to 1.46)		282/52,281	Q2	1.22 (1.03 to 1.44)
	Q3	562/41,494	1.37 (1.21 to 1.54)		321/49,870	Q3	1.41 (1.19 to 1.66)
	Q4	747/43,231	1.73 (1.54 to 1.94)		442/53,817	Q4	1.74 (1.49 to 2.03)
	Q5	871/41,359	2.05 (1.84 to 2.30)		533/47,819	Q5	2.22 (1.91 to 2.59)

Models stratified by age at risk and ethnicity and adjusted for Townsend Deprivation Index, smoking, and alcohol drinking. 95% CI, 95% confidence interval; BMI, body mass index; HR, hazard ratio; Q, quintile; REF, reference quintile; WC, waist circumference; WHR, waist–hip ratio.

In combined and sex-stratified analyses, the association of BMI with incident kidney stone disease was attenuated after adjustment for WC, whereas BMI remained positively associated with kidney stone disease after adjustment for WHR (Figure 2, Supplementary Figure 3). WC and WHR remained positively associated with kidney stone disease after adjustment for BMI (Figure 2, Supplementary Figure 3).

### Genome-Wide Association Study of Kidney Stone Disease in UK Biobank

**Figure 3:**
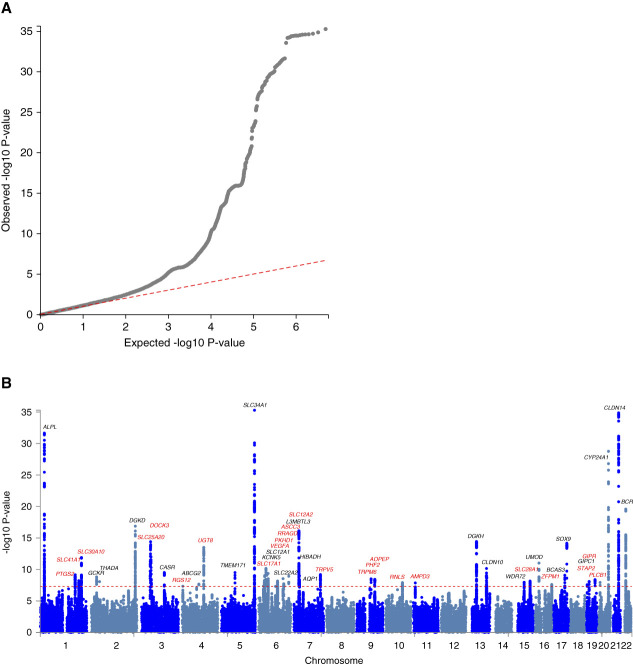
**Results of meta-analysis of GWAS in kidney stone disease (KSD) in UK Biobank and FinnGen study.** A meta-analysis of GWAS of KSD was performed for 17,101 individuals with KSD and 721,947 controls from the UK Biobank and FinnGen study. (A) Is a quantile–quantile plot of observed versus expected *P*-values. The λGC demonstrated some inflation (1.12), but the LD score regression (LDSC) intercept of 1.03, with an attenuation ratio of 0.10, indicated that the inflation was largely due to polygenicity and the large sample size. (B) Is a Manhattan plot showing the genome-wide *P* values (-log10) plotted against their respective positions on each of the chromosomes? The horizontal red line indicates the genome-wide significance threshold of 5.0 × 10−8. Loci have been labeled with the primary candidate gene at each locus, as shown in Table 2. Novel loci are shown in red.

To facilitate MR analyses, we extended our previous genome-wide association study (GWAS) of kidney stone disease in the UK Biobank, using a greater number of inclusion codes to optimize ascertainment (Supplementary Tables 2–3)^2^; GWAS were performed in combined-sex, male, and female populations (Supplementary Tables 6–9, Supplementary Figures 4–6). In addition, we undertook a meta-analysis (combined-sex outcomes) using data from UK Biobank (8504 cases and 388,819 controls) and FinnGen r8 (8597 cases and 333,128 controls).^2^ Meta-analysis revealed 55 independent signals at 47 genetic loci that are associated with kidney stone disease; of these, 23 loci are novel (candidate genes in proximity to association signals: *PTGS2*, *SLC41A1*, *SLC30A10*, *SLC25A20*, *DOCK3*, *RGS12*, *UGT8*, *SLC17A1*, *VEGFA*, *PKHD1*, *RRAGD*, *ASCC3*, *SLC2A12*, *TRPV5*, *TRPM6*, *AOPEP*, *RNLS*, *AMPD3*, *SLC28A1*, *ZFPM1*, *STAP2*, *GIPR*, and *PLCB1*, Table 3, Figure 3).

**Table 3:** Single-nucleotide polymorphisms significantly associated with kidney stone disease from combined-sex meta-analysis of UK Biobank and FinnGen study

Locus	Chr	Pos	rsID	Candidate Gene	EA	NEA	EAF	Meta-analysis OR (95% CI)	Meta-analysis *P*-Value	UK Biobank OR (95% CI)	UK Biobank *P*-Value	FinnGen OR (95% CI)	FinnGen *P*-Value	Het I^2^	Het *P*-Value
1	1	rs116799286	21658347	*ALPL*	G	T	0.04	1.22 (1.15 to 1.3)	9.70×10−10	1.09 (0.95 to 1.26)	0.22	1.28 (1.19 to 1.37)	1.40×10−11	73.10	0.05
1	1	rs115239632	21826530	*ALPL*	T	C	0.05	1.33 (1.27 to 1.4)	2.26×10−32	1.34 (1.24 to 1.46)	2.90×10−13	1.33 (1.25 to 1.41)	1.03×10−20	0.00	0.81
1	1	rs1256332	21893344	*ALPL*	A	C	0.14	1.17 (1.13 to 1.2)	1.57×10−22	1.17 (1.12 to 1.22)	5.40×10−14	1.13 (1.08 to 1.19)	3.32×10−7	10.50	0.29
2	1	rs4648298	186641682	*PTGS2* ^a^	C	T	0.02	1.25 (1.16 to 1.34)	7.04×10−10	1.24 (1.12 to 1.37)	2.70×10−5	1.26 (1.14 to 1.39)	6.16×10−6	0.00	0.79
3	1	rs823130	205714372	*SLC41A1* ^a^	T	C	0.44	1.07 (1.04 to 1.09)	6.12×10−9	1.06 (1.03 to 1.09)	1.80×10−4	1.07 (1.04 to 1.11)	7.55×10−6	0.00	0.61
4	1	rs6694088	220076288	*SLC30A10* ^a^	T	A	0.59	1.08 (1.06 to 1.11)	1.28×10−12	1.08 (1.05 to 1.12)	7.00×10−7	1.08 (1.05 to 1.12)	3.87×10−7	0.00	0.96
5	2	rs6753534	27752871	*GCKR*	C	T	0.42	1.07 (1.05 to 1.09)	1.57×10−9	1.08 (1.04 to 1.11)	3.70×10−6	1.06 (1.03 to 1.1)	9.35×10−5	0.00	0.63
6	2	rs1430083	43448479	*THADA*	A	T	0.76	1.08 (1.05 to 1.11)	8.59×10−9	1.11 (1.07 to 1.17)	2.50×10−6	1.07 (1.03 to 1.1)	2.58×10−4	58.60	0.12
7	2	rs838717	234296444	*DGKD*	G	A	0.42	1.1 (1.08 to 1.12)	1.34×10−17	1.1 (1.06 to 1.13)	2.90×10−9	1.1 (1.07 to 1.14)	8.27×10−10	0.00	0.87
8	3	rs74780677	48601774	*SLC25A20* ^a^	A	G	0.94	1.25 (1.18 to 1.32)	1.58×10−14	1.12 (0.99 to 1.27)	0.07	1.28 (1.2 to 1.37)	1.44×10−14	72.10	0.06
8	3	rs200495345	48861058	*SLC25A20* ^a^	A	AT	0.94	1.31 (1.22 to 1.4)	3.99×10−15			1.31 (1.22 to 1.4)	3.99×10−15	0.00	1.00
9	3	rs138789058	52124388	*DOCK3* ^a^	C	T	0.93	1.19 (1.13 to 1.25)	5.48×10−10	1.07 (0.94 to 1.21)	0.30	1.22 (1.15 to 1.3)	1.47×10−10	72.70	0.06
10	3	rs34172859	121942713	*CASR*	G	GA	0.28	1.08 (1.05 to 1.11)	3.07×10−10	1.07 (1.04 to 1.11)	8.00×10−5	1.09 (1.05 to 1.12)	8.09×10−7	0.00	0.61
11	4	rs4498196	3747842	*RGS12* ^a^	A	C	0.61	1.06 (1.04 to 1.09)	4.63×10−8	1.05 (1.02 to 1.08)	2.30×10−3	1.08 (1.05 to 1.11)	2.65×10−6	30.80	0.23
12	4	rs2231142^b^	89052323	*ABCG2*	T	G	0.10	1.11 (1.07 to 1.15)	2.18×10−8	1.12 (1.07 to 1.18)	2.80×10−6	1.09 (1.03 to 1.16)	1.80×10−3	0.00	0.53
13	4	rs6857337	115588961	*UGT8* ^a^	T	A	0.28	1.1 (1.07 to 1.12)	3.28×10−14	1.08 (1.05 to 1.12)	1.20×10−5	1.11 (1.08 to 1.15)	3.38×10−10	5.20	0.30
14	5	rs638333^b^	72419267	*TMEM171*	C	T	0.27	1.08 (1.06 to 1.11)	3.12×10−10	1.07 (1.03 to 1.11)	1.10×10−4	1.09 (1.06 to 1.13)	4.79×10−7	0.00	0.39
15	5	rs10051765	176799992	*SLC34A1*	C	T	0.37	1.15 (1.13 to 1.18)	5.19×10−36	1.16 (1.12 to 1.2)	8.70×10−20	1.15 (1.11 to 1.18)	6.21×10−18	0.00	0.54
16	6	rs13191296	25684606	*SLC17A1* ^a^	C	T	0.92	1.13 (1.08 to 1.18)	4.43×10−8	1.13 (1.07 to 1.19)	3.60×10−6	1.12 (1.04 to 1.2)	3.42×10−3	0.00	0.81
17	6	rs10807202	39184039	*KCNK5*	T	C	0.26	1.08 (1.06 to 1.11)	6.84×10−11	1.1 (1.06 to 1.14)	8.20×10−8	1.07 (1.03 to 1.11)	8.73×10−5	37.20	0.21
18	6	rs9472142	43818942	*VEGFA* ^a^	T	C	0.31	1.08 (1.05 to 1.1)	3.74×10−10	1.07 (1.03 to 1.1)	1.00×10−4	1.09 (1.05 to 1.12)	6.92×10−7	0.00	0.49
19	6	rs2206271	50786008	*PKHD1* ^a^	A	T	0.32	1.08 (1.05 to 1.1)	4.37×10−10	1.06 (1.02 to 1.09)	1.70×10−3	1.1 (1.06 to 1.13)	1.91×10−8	60.50	0.11
20	6	rs2478887	51236318	*PKHD1* ^a^	G	A	0.91	1.12 (1.08 to 1.17)	2.95×10−9	1.1 (1.05 to 1.16)	7.40×10−5	1.16 (1.09 to 1.24)	4.25×10−6	38.50	0.20
21	6	rs148684631	90121976	*RRAGD* ^a^	G	GGAGA	0.68	1.07 (1.04 to 1.09)	4.95×10−8	1.07 (1.03 to 1.11)	1.40×10−4	1.07 (1.03 to 1.1)	9.76×10−5	0.00	0.92
22	6	rs6928418	101175347	*ASCC3* ^a^	T	C	0.55	1.07 (1.04 to 1.09)	6.82×10−9	1.07 (1.04 to 1.1)	2.60×10−5	1.06 (1.03 to 1.1)	6.69×10−5	0.00	0.89
23	6	rs7740107	130374461	*L3MBTL3*	A	T	0.74	1.08 (1.05 to 1.1)	2.89×10−9	1.08 (1.05 to 1.12)	7.10×10−6	1.07 (1.04 to 1.11)	9.55×10−5	0.00	0.70
23	6	rs9389108	134170549	*SLC12A2* ^a^	A	G	0.34	1.07 (1.04 to 1.09)	1.89×10−8	1.08 (1.05 to 1.12)	3.90×10−7	1.05 (1.01 to 1.08)	4.90×10−3	50.70	0.15
24	6	rs74495751	160624947	*SLC22A2*	C	G	0.03	1.3 (1.2 to 1.42)	1.18×10−9	1.3 (1.2 to 1.42)	1.20×10−9			0.00	1.00
25	7	rs1404278	27634726	*HIBADH*	T	C	0.31	1.1 (1.08 to 1.13)	7.52×10−17	1.12 (1.08 to 1.16)	1.90×10−11	1.09 (1.05 to 1.12)	3.63×10−7	30.60	0.23
26	7	rs1004317	30956858	*AQP1*	G	A	0.39	1.07 (1.05 to 1.09)	1.01×10−9	1.07 (1.04 to 1.11)	1.20×10−5	1.07 (1.04 to 1.1)	2.00×10−5	0.00	0.95
27	7	rs4252512	142605221	*TRPV5* ^a^	C	T	0.02	1.44 (1.28 to 1.61)	6.46×10−10	1.44 (1.28 to 1.61)	6.50×10−10			0.00	1.00
28	9	rs12378991	77472066	*TRPM6* ^a^	A	G	0.07	1.13 (1.09 to 1.18)	3.09×10−9	1.15 (1.09 to 1.22)	1.20×10−6	1.12 (1.05 to 1.19)	5.11×10−4	0.00	0.48
29	9	rs41281168	97480140	*AOPEP* ^a^	A	T	0.91	1.13 (1.08 to 1.17)	3.69×10−9	1.1 (1.04 to 1.18)	1.80×10−3	1.15 (1.09 to 1.21)	3.75×10−7	0.00	0.39
30	10	rs11202736	90142203	*RNLS* ^a^	T	A	0.32	1.07 (1.04 to 1.09)	1.23×10−8	1.05 (1.02 to 1.09)	2.70×10−3	1.09 (1.05 to 1.12)	5.41×10−7	39.90	0.20
31	11	rs1450270	10458596	*AMPD3* ^a^	T	C	0.49	1.06 (1.04 to 1.09)	1.38×10−8	1.06 (1.03 to 1.1)	6.90×10−5	1.07 (1.03 to 1.1)	5.19×10−5	0.00	0.96
32	13	rs9533022	42749606	*DGKH*	C	T	0.42	1.09 (1.07 to 1.12)	3.68×10−15	1.11 (1.07 to 1.14)	1.90×10−10	1.08 (1.04 to 1.11)	1.97×10−6	22.90	0.25
33	13	rs57719175	96175396	*CLDN10*	G	A	0.60	1.08 (1.05 to 1.1)	6.98×10−11	1.07 (1.03 to 1.1)	3.50×10−5	1.09 (1.05 to 1.12)	3.54×10−7	0.00	0.46
34	15	rs201275031	53977631	*WDR72*	T	C	0.51	1.09 (1.06 to 1.12)	2.22×10−8	1.09 (1.06 to 1.12)	2.20×10−8			0.00	1.00
35	15	rs71397835	85530771	*SLC28A1* ^a^	T	C	0.15	1.13 (1.09 to 1.18)	7.11×10−9	1.13 (1.09 to 1.18)	7.10×10−9			0.00	1.00
36	16	rs12921916	20407196	*UMPD*	C	T	0.28	1.09 (1.06 to 1.11)	9.58×10−12	1.11 (1.07 to 1.14)	4.30×10−9	1.07 (1.03 to 1.11)	1.82×10−4	51.80	0.15
37	16	rs34442094	88545395	*ZFPM1* ^a^	T	G	0.34	1.07 (1.04 to 1.09)	2.44×10−8	1.07 (1.04 to 1.1)	2.90×10−5	1.06 (1.03 to 1.1)	2.15×10−4	0.00	0.76
38	17	rs117518238	59462062	*BCAS3*	C	T	0.92	1.21 (1.14 to 1.28)	8.44×10−−10			1.21 (1.14 to 1.28)	8.44×10−10	0.00	1.00
39	17	rs2240736	59485393	*BCAS3*	T	C	0.73	1.07 (1.04 to 1.1)	3.80×10−8	1.05 (1.01 to 1.09)	6.30×10−3	1.09 (1.06 to 1.13)	4.99×10−7	62.10	0.10
40	17	rs11077601	70350140	*SOX9*	T	G	0.48	1.09 (1.07 to 1.11)	6.83×10−15	1.08 (1.05 to 1.11)	6.40×10−7	1.1 (1.07 to 1.13)	1.57×10−9	0.00	0.45
41	19	rs3810368	4342730	*STAP2* ^a^	G	A	0.68	1.07 (1.04 to 1.09)	2.62×10−8	1.07 (1.04 to 1.11)	4.20×10−5	1.06 (1.03 to 1.1)	1.60×10−4	0.00	0.77
42	19	rs3760702	14588237	*GIPC1*	A	G	0.30	1.07 (1.05 to 1.1)	8.15×10−9	1.09 (1.05 to 1.12)	5.10×10−7	1.06 (1.02 to 1.09)	2.21×10−3	30.90	0.23
43	19	rs11672660	46180184	*GIPR* ^a^	C	T	0.77	1.08 (1.05 to 1.11)	4.22×10−9	1.08 (1.04 to 1.13)	4.70×10−5	1.08 (1.04 to 1.12)	2.26×10−5	0.00	0.90
44	20	rs6055748	8315317	*PLCB1* ^a^	G	A	0.32	1.07 (1.05 to 1.09)	1.16×10−8	1.09 (1.05 to 1.12)	8.20×10−7	1.05 (1.02 to 1.09)	1.57×10−3	42.70	0.19
45	20	rs6123359	52714706	*CYP24A1*	G	A	0.11	1.1 (1.06 to 1.14)	2.15×10−8	1.1 (1.05 to 1.16)	1.40×10−4	1.16 (1.11 to 1.22)	3.03×10−10	55.40	0.13
45	20	rs6127099	52731402	*CYP24A1*	A	T	0.73	1.15 (1.12 to 1.18)	1.74×10−29	1.14 (1.1 to 1.18)	2.80×10−14	1.16 (1.12 to 1.2)	7.16×10−17	0.00	0.53
45	20	rs2585442	52737123	*CYP24A1*	G	C	0.29	1.08 (1.05 to 1.11)	4.69×10−10	1.12 (1.08 to 1.16)	5.40×10−10	1.12 (1.09 to 1.16)	4.46×10−12	0.00	0.95
45	20	rs2762943	52790786	*CYP24A1*	G	T	0.91	1.15 (1.1 to 1.19)	7.88×10−12	1.13 (1.06 to 1.19)	4.00×10−5	1.2 (1.14 to 1.27)	4.47×10−11	60.50	0.11
46	21	rs128494	37834258	*CLDN14*	C	T	0.70	1.08 (1.05 to 1.1)	1.09×10−9	1.04 (1 to 1.08)	0.05	1.02 (0.98 to 1.05)	0.36	0.00	0.37
46	21	rs219772	37835347	*CLDN14*	A	T	0.76	1.18 (1.15 to 1.21)	1.36×10−35	1.17 (1.13 to 1.21)	1.70×10−19	1.18 (1.14 to 1.23)	9.30×10−18	0.00	0.74
47	22	rs13054904	23410918	*BCR*	A	T	0.23	1.13 (1.1 to 1.16)	2.60×10−20	1.15 (1.11 to 1.19)	8.30×10−16	1.1 (1.06 to 1.14)	1.03×10−6	70.60	0.07

95% CI, 95% confidence interval; Chr, chromosome; EA, effect allele; EAF, effect allele frequency in kidney stone formers; Het, heterogeneity; NEA, noneffect allele; OR, odds ratio; Pos, position based on NCBI Genome Build 37 (hg19).

a Novel loci.

b Coding region variant.

Sex-specific GWAS in the UK Biobank identified 3 and 15 independent signals associated with kidney stone disease in female and male participants, respectively. All signals were directionally concordant in analyses of male participants and female participants; however, only *SLC34A1*, *UMOD*, *CYP24A1*, and *CLDN14* loci reached GWAS replication significance threshold (*P*<5.0×10^−5^) in both sexes (Supplementary Tables 8–9, Supplementary Figures 5–6).

For UK Biobank, FinnGen, and meta-analysis GWAS, SNP-based heritability of kidney stone disease was approximately 20% (Supplementary Table 12). Gene-set analyses in MAGMA indicated a role for hypermagnesemia, hypocalciuria, and abnormal blood inorganic cation concentration in the pathogenesis of kidney stone disease (Supplementary Table 10). Seventy-three candidate genes were identified through *in silico* analysis of the 47 loci identified at meta-analysis on the basis of FUMA positional mapping, functional annotation, and biologic plausibility. Gene property analysis implemented in FUMA revealed an overexpression of these genes in the kidney cortex; the GENE2FUNC tool demonstrated enrichment of 12 genes, including *CASR*, *CLDN10*, *CLDN14*, *KCNK5*, *SLC28A1*, and *UGT8*, in GTEx v8 kidney cortex tissue (Supplementary Tables 10–11, Supplementary Figure 7).

### Effects of Adiposity on Risk of Kidney Stone Disease

MR analyses using kidney stone meta-analysis results demonstrated that a one SD higher genetically determined BMI,^38^ WC,^56^ and WHR^38^ (Supplementary Table 4) resulted in an odds ratio (OR) for kidney stone disease of 1.36 (95% CI=1.25–1.48), 1.35 (95% CI=1.07–1.71), and 1.33 (95% CI=1.18–1.50), respectively (Table 4, Supplementary Figure 8). The Cochrane Q test suggested significant heterogeneity in IVs (Table 4), and pairwise genetic correlations of summary statistics from kidney stone disease meta-analysis and BMI,^38^ WHR,^38^ and WC^56^ indicated a shared genetic etiology (Supplementary Table 12).

**Table 4:** Univariable Mendelian randomization analyses in meta-analysis of UK Biobank and FinnGen study

Analysis				Inverse-Variance Weighted				Intercept		MR Egger			
				Estimate		Heterogeneity				Estimate		Heterogeneity	
Group	Exposure	Outcome	*N* SNP	OR (95% CI)	*P* ^a^	Q	*P*	Beta (SE)	*P*	OR (95% CI)	*P*	Q	*P*
Adiposity	ASAT	KSD	4	1.73 (0.97 to 3.1)^b^	0.15^b^	20.15	1.58×10−4	0.01 (0.07)	0.86	1.38 (0.13 to 14.39)	0.81	19.75	5.14×10−5
Adiposity	ASAT/GFAT	KSD	13	1.1 (0.79 to 1.54)^b^	0.73^b^	80.6	3.17×10−12	−0.02 (0.04)	0.53	1.71 (0.44 to 6.62)	0.45	77.57	4.35×10−12
Adiposity	BMI	KSD	569	1.36 (1.25 to 1.48)^b^	1.30x10^−10^^b^	1000.77	2.36×10−26	−1.86×10−3 (1.91×10−3)	0.33	1.51 (1.2 to 1.91)	5.46×10−4	999.09	2.56×10−26
Adiposity	BMI-IVs in WHR genetic instrument removed	KSD	543	1.39 (1.27 to 1.53)^b^	1.48x10^−11^^b^	945.18	2.56×10−24	−3.12×10−03 (2.04×10−3)	0.13	1.68 (1.3 to 2.17)	7.59×10−5	941.11	4.65×10−24
Adiposity	GFAT	KSD	16	1.05 (0.79 to 1.39)^b^	0.86^b^	74.43	7.18×10−10	1.11×10−3 (0.03)	0.97	1.03 (0.34 to 3.06)	0.96	74.42	3.02×10−10
Adiposity	VAT	KSD	3	1.5 (0.64 to 3.54)^b^	0.58^b^	19.49	5.85×10−5	0.01 (0.34)	0.98	1.18 (9.44e−07 to 1470,111.75)	0.98	19.47	1.02×10−5
Adiposity	VAT/ASAT	KSD	18	1.11 (0.99 to 1.25)^b^	0.18^b^	24.26	0.11	5.07×10−3 (0.01)	0.68	1.03 (0.71 to 1.49)	0.87	23.99	0.09
Adiposity	VAT/GFAT	KSD	19	1.2 (0.97 to 1.49)^b^	0.18^b^	73.07	1.35×10−8	−2.61×10−4 (0.02)	0.99	1.21 (0.64 to 2.29)	0.57	73.07	6.33×10−9
Adiposity	WC	KSD	43	1.35 (1.07 to 1.71)^b^	0.03^b^	120.72	1.53×10−9	9.45×10−3 (0.01)	0.40	0.97 (0.43 to 2.18)	0.94	118.67	1.76×10−9
Adiposity	WHR	KSD	265	1.33 (1.18 to 1.5)^b^	4.47x10^−5^^b^	473.41	4.36×10−14	−1.17×10−3 (2.83×10−3)	0.68	1.42 (1.02 to 1.97)	0.04	473.1	3.46×10−14
Adiposity	WHR-IVs in BMI genetic instrument removed	KSD	239	1.33 (1.17 to 1.51)^b^	6.71x10^−5^^b^	427.26	6.23e−13	1.50×10−05 (2.95×10−03)	1.00	1.33 (0.94 to 1.88)	0.11	427.26	4.62×10−13
Biochemistry	Heel bone mineral density	KSD	977	0.95 (0.91 to 1)^b^	0.14^b^	1833.59	5.34×10−55	−7.04×10−4 (1.17×10−3)	0.55	0.97 (0.89 to 1.07)	0.59	1832.9	4.57×10−55
Biochemistry	Serum 25-OH vitamin D concentration	KSD	162	1.23 (1.07 to 1.42)^b^	0.02^b^	539.78	2.07×10−42	−1.75×10−3 (3.17×10−3)	0.58	1.29 (1.04 to 1.61)	0.02	538.76	1.61×10−42
Biochemistry	Serum calcium concentration^c^	KSD	180	1.48 (1.25 to 1.76)^b^	5.31x10^−5^^b^	1289.53	2.73×10−167	−5.95×10−3 (5.34×10−3)	0.27	1.74 (1.25 to 2.42)	1.12×10−3	1280.59	4.78×10−166
Biochemistry	Serum phosphate concentration	KSD	90	0.71 (0.59 to 0.87)^b^	5.20x10^−3^^b^	388.82	4.32×10−39	−6.72×10−3 (5.54×10−3)	0.23	0.85 (0.6 to 1.2)	0.36	382.44	2.45×10−38
Biochemistry	Urate	KSD	337	0.97 (0.88 to 1.07)^b^	0.73^b^	1170.33	9.34×10−93	−4.21×10−3 (2.17×10−3)	0.05	1.08 (0.93 to 1.25)	0.3	1157.4	5.05×10−91
Metabolic syndrome	2-hour glucose tolerance	KSD	12	0.95 (0.71 to 1.25)^b^	0.85^b^	81.75	6.76×10−13	0.03 (0.03)	0.21	0.61 (0.3 to 1.23)	0.2	69.39	5.80×10−11
Metabolic syndrome	DBP	KSD	747	1 (0.99 to 1.01)^b^	0.9^b^	1432.28	9.80×10−46	−1.61×10−3 (1.71×10−3)	0.35	1.01 (0.99 to 1.03)	0.43	1430.57	1.07×10−45
Metabolic syndrome	Fasting glucose adjusted for BMI	KSD	85	1.03 (0.87 to 1.23)^b^	0.86^b^	157.97	1.90×10−6	4.32×10−3 (3.75×10−3)	0.25	0.88 (0.65 to 1.21)	0.44	155.47	2.52×10−6
Metabolic syndrome	Fasting insulin adjusted for BMI	KSD	41	1.48 (0.92 to 2.4)^b^	0.22^b^	148.28	2.36×10−14	3.78×10−3 (0.01)	0.77	1.2 (0.27 to 5.35)	0.81	147.95	1.35×10−14
Metabolic syndrome	HbA1c	KSD	69	1.23 (0.95 to 1.59)^b^	0.22^b^	124.05	3.93×10−5	−1.30×10−3 (4.14×10−3)	0.75	1.32 (0.8 to 2.16)	0.28	123.87	2.96×10−5
Metabolic syndrome	SBP	KSD	702	1 (1 to 1.01)^b^	0.71^b^	1369.81	1.23×10−45	1.90×10−3 (1.78×10−3)	0.29	1 (0.98 to 1.01)	0.48	1367.57	1.53×10−45
Metabolic syndrome	Serum HDL concentration	KSD	611	1.02 (0.96 to 1.08)^b^	0.72^b^	1081.74	8.14×10−29	−2.35×10−3 (1.23×10−3)	0.06	1.09 (1 to 1.19)	0.05	1075.24	2.55×10−28
Metabolic syndrome	Serum LDL concentration	KSD	271	0.99 (0.92 to 1.07)^b^	0.87^b^	520.07	4.94×10−18	2.96×10−3 (1.89×10−3)	0.12	0.93 (0.83 to 1.04)	0.18	515.37	1.12×10−17
Metabolic syndrome	Serum TG concentration	KSD	511	1.12 (1.05 to 1.2)	4.09x10−3	1007.63	5.72×10−35	2.95×10−3 (1.40×10−3)	0.04	1.03 (0.92 to 1.14)^b^	0.61^b^	998.89	3.55×10−34
Metabolic syndrome	T2D	KSD	354	1.09 (1.05 to 1.14)	1.45x10−4	780.4	2.47×10−34	4.65×10−3 (2.17×10−3)	0.03	1.01 (0.92 to 1.1)^b^	0.9^b^	770.34	2.65×10−33
Systemic inflammation	APO-B	KSD	313	1.00 (0.94 to 1.06)^b^	0.92^b^	516.01	2.92×10−12	2.26×10−3 (1.64×10−3)	0.17	0.95 (0.87 to 1.04)	0.27	512.87	4.25×10−12
Systemic inflammation	CRP	KSD	66	1.06 (0.95 to 1.18)^b^	0.51^b^	227.6	6.59×10−20	3.42×10−3 (4.46×10−3)	0.45	1.01 (0.86 to 1.19)	0.91	225.53	7.36×10−20

Group	Exposure	Outcome	*N* SNP	Beta (SE)	*P* ^a^	Q	*P*	Beta (SE)	*P*	Beta (SE)	*P*	Q	*P*
Adiposity	KSD	WC	36	6.76e−03 (0.02)^b^	0.86^b^	171.44	6.71×10−20	3.84×10−3 (5.82×10−3)	0.51	−0.03 (0.06)	0.61	169.27	7.14×10−20
Adiposity	KSD	WHR	50	0.02 (8.14e−03)^b^	0.09^b^	275.47	2.69×10−33	−2.64×10−4 (2.14×10−3)	0.9	0.02 (0.02)	0.38	275.38	1.16×10−33
Adiposity	BMI	Urine calcium excretion	587	−7.94e−03 (5.13e−03)	0.23	619.47	0.16	2.25×10−3 (7.04×10−4)	1.50×10−3	−0.18 (0.06)^b^	9.55×10−4^b^	608.88	0.24
Adiposity	BMI	WHR	596	0.48 (0.01)^b^	3.81×10−272^b^	4136.42	0.00	−5.02×10−4 (5.88×10−4)	0.39	0.51 (0.04)	2.05×10−38	4131.34	0.00
Adiposity	WHR	24-h urine calcium excretion	284	5.04e−03 (7.32e−03)^b^	0.73^b^	324.31	0.05	1.44×10−3 (9.87×10−4)	0.15	−0.09 (0.07)	0.17	321.87	0.05
Adiposity	WHR	BMI	287	0.47 (0.04)	2.48×10−30	10,439.33	0.00	7.46×10−3 (1.97×10−3)	1.84×10−4	0.05 (0.12)^b^	0.68^b^	9938.47	0.00
Biochemistry	KSD	Serum calcium concentration^c^	54	0.03 (0.03)^b^	0.47^b^	2577.14	0.00	−0.01 (0.01)	0.31	0.11 (0.08)	0.18	2525.85	0.00
Biochemistry	KSD	Serum calcium concentration^c^	54	0.03 (0.03)^b^	0.47^b^	2577.14	0.00	−0.01 (0.01)	0.31	0.11 (0.08)	0.18	2525.85	0.00

APO-B, apolipoprotein-B; ASAT, abdominal subcutaneous adipose tissue; BMI, body mass index; CI, confidence interval; CRP, C-reactive protein; GFAT, gluteofemoral adipose tissue; HDL, high-density lipoprotein; LDL, low-density lipoprotein; KSD, kidney stone disease; LDL, low-density lipoprotein; *N* SNP, number of single nucleotide polymorphisms included in analysis; OR, odds ratio for outcome per 1 standard deviation increase in genetically instrumented exposure variable; SE, standard error; T2D, type 2 diabetes; TG, triglyceride; VAT, visceral adipose tissue; WHR, waist-to-hip ratio; 25-OH vitamin D, hydroxyvitamin D.

a *P* value adjusted for multiple testing using the false discovery rate method.

b The sensitivity analysis to be interpreted after considering the estimate of the intercept.

c Albumin-adjusted serum calcium concentration.

**Figure 4:**
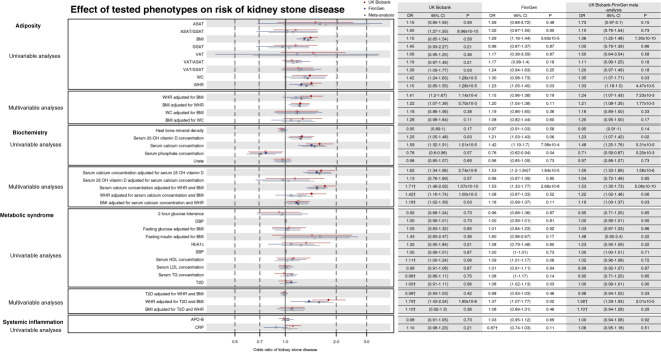
**Effect of tested phenotypes on risk of kidney stone disease in Mendelian randomization analyses.** Odds ratios (OR) and 95% confidence intervals (95% CI) of kidney stone disease per 1-standard deviation higher genetically instrumented exposure variable. Nonsignificant results are indicated by hollow symbols. Estimates, by default, refer to inverse-variance weighted (IVW) estimates; where MR-Egger estimate has been used, this is indicated by † annotation. APO-B, apolipoprotein-B; BMI, body mass index; CRP, C-reactive protein; HDL, high-density lipoprotein; LDL, low-density lipoprotein; T2D, type 2 diabetes; WC, waist circumference; WHR, waist-to-hip ratio.

MR results were directionally concordant in UK Biobank and FinnGen analyses and robust to Steiger filtering (Figure 4, Supplementary Tables 13–16). When overlapping IVs were removed from BMI and WHR^38^ genetic instruments, both retained a significant effect on risk of kidney stone disease (Supplementary Table 13). Furthermore, multivariable MR estimates indicated that measures of general and central adiposity are independent, causal risk factors for kidney stone disease. Thus, in the UK Biobank-FinnGen meta-analysis, the effect of BMI^38^ on risk of kidney stone disease after adjustment for WHR^38^ was 1.21 (95% CI=1.08–1.35), the effect of WC^56^ on risk of kidney stone disease after adjustment for BMI^38^ was 1.16 (95% CI=0.89–1.50), and the effect of WHR^38^ on risk of kidney stone disease after adjustment for BMI^38^ was 1.24 (95% CI=1.07–1.43) (Table 5, Supplementary Tables 17–18, Figure 4). Bidirectional MR identified that increasing BMI^38^ increases WHR^38^; however, no effects of increasing WHR^38^ on BMI^38^ were detected after accounting for horizontal pleiotropy (Table 4, Supplementary Figure 8). No causal effects of genetically proxied measures of MRI-derived visceral, abdominal subcutaneous, or gluteofemoral adipose tissue volumes, or ratios of fat deposits^39^ on nephrolithiasis were detected (Table 4, Supplementary Tables 13–14, Figure 4). Elevated WC did not exert an effect on risk of kidney stones in female participants (OR=1.16, 95%, CI=0.71–1.91); however, there was no other evidence for sex-specific effects of adiposity on risk of kidney stone disease (Supplementary Tables 13 and 15–17, Supplementary Figure 6), nor evidence that kidney stones causally increase markers of adiposity^38,56^ (Table 4, Supplementary Table 13).

**Table 5:** Multivariable Mendelian randomization analyses in meta-analysis of UK Biobank and FinnGen study

Analysis					Inverse-Variance Weighted				Intercept		MR Egger			
					Estimate		Heterogeneity				Estimate		Heterogeneity	
Multivariable Model	Exposure	Adjusted for	Outcome	*N* SNP	OR (95% CI)	*P*	Q	*P*	Beta (SE)	*P*	OR (95% CI)	*P*	Q	*P*
WHR and BMI	WHR	BMI	KSD	735	1.24 (1.07 to 1.43)^a^	7.23×10−3^a^	1240.66	1.00×10−28	−1.00×10−3 (1.00×10−3)	0.48	1.32 (1.06 to 1.63)	0.01	1239.8	9.11×10−29
	BMI	WHR	KSD	735	1.21 (1.08 to 1.35)^a^	1.77×10−3^a^	1240.66	1.00×10−28	−1.00×10−3 (1.00×10−3)	0.48	1.24 (1.09 to 1.42)	1.00×10−3	1239.8	9.11×10−29
WC and BMI	WC	BMI	KSD	555	1.16 (0.89 to 1.50)^a^	0.33^a^	938.63	2.11×10−22	9.80×10−5 (2.00×10−3)	0.95	1.16 (0.89 to 1.51)	0.28	938.63	1.62×10−22
	BMI	WC	KSD	555	1.20 (0.95 to 1.50)^a^	0.17^a^	938.63	2.11×10−22	9.80×10−5 (2.00×10−3)	0.95	1.19 (0.92 to 1.54)	0.19	938.63	1.62×10−22
Serum calcium concentration^b^ and BMI and WHR	Serum calcium concentration^b^	WHR and BMI	KSD	850	1.53 (1.35 to 1.73)^a^	5.06×10−10^a^	2233.05	2.48×10−125	−4.58×10−4 (1.32×10−3)	0.73	1.58 (1.4 to 1.77)	4.32×10−14	2062.07	5.44×10−103
	WHR	Serum calcium concentration^b^ and BMI	KSD	850	1.22 (1.02 to 1.46)^a^	0.06^a^	2233.05	2.48×10−125	−4.58×10−4 (1.32×10−3)	0.73	1.22 (1.02 to 1.45)	0.03	2062.07	5.44×10−103
	BMI	Serum calcium concentration^b^ and WHR	KSD	850	1.19 (1.03 to 1.37)^a^	0.03^a^	2233.05	2.48×10−125	−4.58×10−4 (1.32×10−3)	0.73	1.22 (0.99 to 1.51)	0.06	2062.07	5.44×10−103
Serum calcium concentration^b^ and serum 25 OH vitamin D	Serum calcium concentration^b^	Serum 25 OH vitamin D	KSD	228	1.59 (1.33 to 1.89)^a^	1.56×10−6^a^	1388.05	4.01×10−166	−7.00×10−3 (4.00×10−3)	0.09	2.03 (1.46 to 2.83)	2.93×10−5	1370.02	3.10×10−163
	Serum 25 OH vitamin D	Serum calcium concentration^b^	KSD	228	1.04 (0.72 to 1.49)^a^	0.85^a^	1388.05	4.01×10−166	−7.00×10−3 (4.00×10−3)	0.09	1.07 (0.74 to 1.53)	0.73	1370.02	3.10×10−163
T2D and BMI and WHR	T2D	WHR and BMI	KSD	588	0.98 (0.94 to 1.02)^a^	0.33^a^	1174.59	7.63×10−42	1.00×10−3 (1.00×10−3)	0.69	0.96 (0.89 to 1.04)	0.36	1174.28	5.81×10−42
	WHR	T2D and BMI	KSD	588	1.58 (1.29 to 1.93)^a^	2.01×10−5^a^	1174.59	7.63×10−42	1.00×10−3 (1.00×10−3)	0.69	1.58 (1.29 to 1.93)	6.69×10−6	1174.28	5.81×10−42
	BMI	T2D and WHR	KSD	588	1.1 (0.94 to 1.28)^a^	0.29^a^	1174.59	7.63×10−42	1.00×10−3 (1.00×10−3)	0.69	1.1 (0.94 to 1.28)	0.22	1174.28	5.81×10−42

APO-B, apolipoprotein-B; BMI, body mass index; CI, confidence interval; CRP, C-reactive protein; HDL, high density lipoprotein; LDL, low-density lipoprotein; KSD, kidney stone disease; LDL, low-density lipoprotein; *N* SNP, number of single-nucleotide polymorphisms included in analysis; OR, odds ratio for outcome per 1 standard deviation increase in genetically instrumented exposure variable; SE, standard error; sIL-6R, serum IL-6 receptor; T2D, type 2 diabetes; TG, triglyceride; WHR, waist-to-hip ratio; 25-OH vitamin D, hydroxyvitamin D.

a The sensitivity analysis to be interpreted after considering the estimate of the intercept.

b Albumin-adjusted serum calcium concentration.

Our findings indicate that general and central adiposity are independent causal factors in the pathogenesis of kidney stone disease. We hypothesized that these factors may exert effects on serum or urinary biochemical phenotypes, features of metabolic syndrome, or inflammation to increase risk of kidney stones and used MR to explore these relationships (Figure 1).

### Effects of Central Adiposity on Serum and Urinary Biochemical Phenotypes

Univariable IVW MR estimates identified that a one SD higher genetically instrumented albumin-adjusted serum calcium^44^ (equivalent to 0.08 mmol/L) was associated with an OR for kidney stone disease of 1.48 (95% CI=1.25–1.76, Table 4, Supplementary Tables 13–14, Figure 4, Supplementary Figure 8); this result was robust to Steiger filtering (Supplementary Tables 15–16). Higher WHR^38^ and BMI^38^ led to increased serum calcium concentrations^44^ in the UK Biobank; after accounting for multiple testing, WC^56^ did not, likely as a result of a lack of statistical power (Supplementary Table 13, Supplementary Figure 8). MR demonstrated that higher serum calcium concentrations^44^ increase WHR^38^ (β=0.03, SE=0.01), but not BMI^38^ (Supplementary Table 13, Supplementary Figure 8).

After the removal of overlapping IVs from BMI^38^ and WHR^38^ genetic instruments, effects of BMI and WHR^38^ on serum calcium concentrations^44^ were not detected, indicating the importance of adiposity in this mechanism (Supplementary Table 13). Multivariable MR suggested that higher WHR^38^ causally increased serum calcium concentrations independent of BMI^38^ (IVW β=0.12 mmol/L, SE=0.02); however, BMI^38^ had no effect on serum calcium concentration^44^ after adjustment for WHR (IVW β=−0.03 mmol/L, SE=0.02, Supplementary Table 17). The effects of WHR on serum calcium concentrations were recapitulated in observational data from UK Biobank; thus, a one SD increase in WHR was associated with a 0.05 mmol/L and 0.09 mmol/L higher albumin-adjusted serum calcium concentration in male participants and female participants, respectively (Supplementary Table 19).

**Figure 5:**
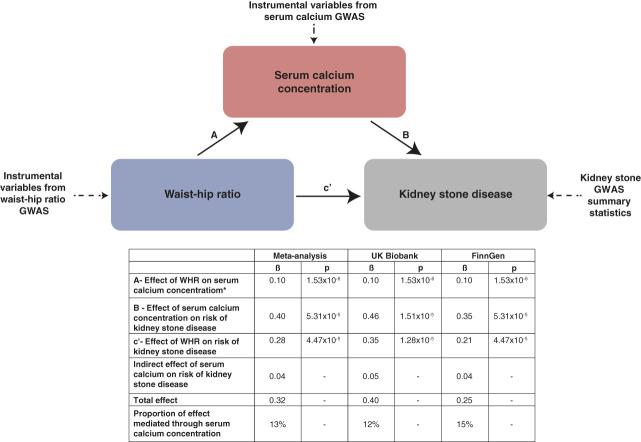
**Mediation Mendelian randomisation—effects of waist-to-hip ratio on serum calcium concentration and kidney stone disease in meta-analysis, UK Biobank, and FinnGen cohorts.** Mediation Mendelian randomization results. β, regression coefficient for each MR analysis; *P*, *P* value adjusted for multiple testing using the 5% false discovery rate method. *Estimate for effect of waist-to-hip ratio on serum calcium concentration is derived from UK Biobank serum calcium data for all analyses.

Multivariable MR demonstrated that the effects of serum calcium concentration^44^ on risk of kidney stone disease were retained after adjustment for WHR^38^ and BMI^38^; however, effects of WHR^38^ and BMI^38^ on risk of kidney stone disease were partially attenuated after adjustment for serum calcium concentration,^44^ in keeping with an effect mediated by this biochemical phenotype (Table 5, Supplementary Tables 17–18, Figure 4). Furthermore, mediation MR demonstrated that 12%–15% of the effect of WHR^38^ on kidney stone disease risk is mediated through alterations in serum calcium concentration^44^ (Table 4, Supplementary Tables 13–14, Figure 5). We postulate that an individual's risk of kidney stone disease may be increased by small increments in adjusted serum calcium concentrations which lie within normal reference ranges.

We hypothesized that adiposity may alter bone resorption to increase serum calcium concentrations. However, on MR analyses, both BMI^38^ and WHR^38^ led to a higher heel bone mineral density^46^ (WHR: β=0.14, SE=0.03, BMI: β=0.12, SE=0.02, Supplementary Table 13, Supplementary Figure 8), while estimated heel bone mineral density decreased serum calcium concentrations with no effect on risk of kidney stone disease (Table 4, Supplementary Tables 13–14, Figure 4, Supplementary Figure 8).

Univariable MR analyses showed that higher serum phosphate concentrations^44^ reduce the risk of kidney stone disease (meta-analysis OR=0.67, 95% CI=0.59 to 0.87) (Table 4, Supplementary Tables 13–14, Figure 4, Supplementary Figure 8). Leave-one-out analyses indicated that this effect is driven by rs10051765 (Supplementary Figures 10–11, Supplementary Data), an intergenic variant which is associated with reduced serum phosphate concentrations (β=−0.05, SE=0.003, *P*=9.80×10^−90^) and increased albumin-adjusted serum calcium concentrations (β=0.03, SE=0.003, *P*=5.30×10^−22^) in GWAS data from the UK Biobank.^44^ After accounting for multiple testing, higher BMI,^38^ WHR,^38^ and WC^56^ had no effect on serum phosphate concentrations^44^ (Supplementary Table 13). Multivariable MR indicated that higher 25-OH vitamin D concentration^57^ was not a risk factor for kidney stone disease after adjustment for serum calcium concentration^44^ (Table 5, Supplementary Tables 17–18, Figure 4). Furthermore, higher WHR^38^ lowered concentrations of 25-OH vitamin D^57^ concentration (β=-0.04, SE=0.02, Supplementary Table 13).

Only one independent SNP was identified from a GWAS of 24-hour urinary calcium excretion precluding confirmation through MR that higher urinary calcium excretion has a causal effect on kidney stone disease. However, neither higher BMI^38^ nor WHR^38^ were found to alter urinary calcium excretion after accounting for Steiger filtering (Table 4, Supplementary Tables 15–16). Serum urate^45^ showed no effect on risk of kidney stone disease (Table 4, Supplementary Tables 13–14).

### Effects of Components of the Metabolic Syndrome and Markers of Inflammation on Kidney Stone Risk

Using MR analyses, no evidence was found that dyslipidemias^42^ associated with metabolic syndrome (triglyceride concentrations, HDL, and LDL cholesterol concentrations^42^) have causal effects on kidney stone disease (Supplementary Tables 13–14).

Previous studies in UK Biobank and FinnGen cohorts have reported causal effects of type 2 diabetes (T2D) on kidney stone disease; we therefore extended our MR analyses to include weighted median and contamination mixture models.^14^ Univariable MR estimates revealed a potential causal pathway linking genetic liability to T2D^40^ with kidney stone disease (Supplementary Table 16). To account for the shared genetic architecture of T2D and adiposity, we undertook multivariable MR adjusting for BMI,^38^ WHR,^38^ and T2D^40^ simultaneously. This indicated that causal effects of T2D on kidney stone disease are likely confounded by coexisting adiposity (Table 5, Supplementary Tables 17–18, Figure 4). Furthermore, we explored the effects of phenotypes associated with T2D, including fasting glucose adjusted for BMI,^41^ fasting insulin adjusted for BMI,^41^ HbA1c,^41^ and 2-hour glucose tolerance,^41^ on risk of kidney stone disease and found no evidence to support causal effects of these diabetic phenotypes on risk of kidney stone disease (Table 4, Supplementary Tables 13–14, Figure 4).

No evidence was found for causal effects of blood pressure,^43^ increasing serum concentrations of CRP,^47^ or apolipoprotein B^42^ on kidney stone disease (Figure 4, Supplementary Tables 13–14).

## Discussion

This study demonstrates that BMI, a marker of general adiposity, and WHR, a marker of central adiposity, are independent risk factors for kidney stone disease. In multivariable observational and genetic analyses of combined-sex cohorts, we found that a one SD higher BMI and WHR results in approximately 19%–21% and approximately 22%–24% increased risk of kidney stone disease, respectively. We report the novel finding that higher WHR causes elevation of serum calcium concentrations, with a 0.08 mmol/L higher serum calcium concentration causing a 42%–59% increased risk of kidney stone disease. Through MR techniques, we show that this pathogenic mechanism mediates 12%–15% of the effect of increasing WHR on risk of kidney stone disease. We predict that small alterations in adjusted serum calcium concentrations, within the normal reference range, increase an individual's risk of kidney stone disease.

Our study highlights that adiposity is an important determinant of kidney stone disease risk. Adipose tissue has multiple functions including energy storage, glucose homeostasis, and endocrine activity and is related to all-cause mortality, cardiovascular disease, and cancer risk.^58–60^ We hypothesize that the effects of central adiposity on serum calcium concentration and risk of kidney stone disease are related to the transcriptional and adipokine profiles of visceral adipose depots affecting on calcium homeostatic pathways. We postulate that therapies targeting adipose depots, for example, glucagon-like peptide-1 receptor agonists, may affect calcium homeostasis and have utility for the prevention of kidney stone disease.^61–64^ Studies are required to investigate these mechanisms and reveal novel therapeutic targets to facilitate improved management strategies for patients with kidney stone.^65^

Kidney stone disease has historically been more common in male patients than female patients; however, epidemiological trends in kidney stone disease indicate that this sex gap is closing.^1^ We found no evidence of genetic or anthropometric sex-specific risk factors for kidney stone disease. Rates of obesity are reported to be increasing at a greater rate in female patients than male patients; thus, it is plausible that variations in adiposity may have driven the previously reported sex differences in kidney stone prevalence.^66^

It has been widely postulated that obesity is linked to risk of kidney stone disease because of associations with metabolic syndrome.^4,5^ Our results, that are based on optimized outcome data, differ from recent studies in finding limited evidence that genetic liability to T2D increases risk of kidney stones and no evidence to support causal effects of dyslipidemias associated with metabolic syndrome on risk of kidney stone formation.^14,15^ Considering the results of our multivariable MR analyses, we propose that the effects of T2D on increasing risk of kidney stone disease are confounded by coexisting adiposity. This study indicates that hypertension does not have a causal effect on risk of nephrolithiasis. Our conclusions contrast with studies demonstrating that the causal effects of adiposity on chronic kidney disease (CKD) are largely mediated by T2D and blood pressure, highlighting the different causal architectures of CKD and kidney stone disease.^67,68^ Furthermore, we found no evidence for causal effects of markers of systemic inflammation or serum urate concentrations on kidney stone formation. These results support observational data reporting no correlation between serum and urinary urate concentrations.^69^

Using MR analyses, we identified that a one SD higher serum phosphate concentration (equivalent to 0.16 mmol/L) reduces the odds of kidney stones by 29–32%. Our GWAS meta-analysis identified an intergenic variant, rs10051765, approximately 6 Kbp upstream of *SLC34A1*, that is associated with an increased risk of kidney stones, increased albumin-adjusted serum calcium, and decreased serum phosphate concentrations, which drives effects of decreasing serum phosphate concentrations on risk of kidney stone disease. *SLC34A1* encodes the renally expressed sodium-phosphate transport protein 2A (NaPi-IIa); biallelic loss-of-function mutations in *SLC34A1* cause idiopathic infantile hypercalcemia type 2 (IH2) as a result of urinary phosphate wasting leading to a reduction in serum FGF23 concentrations that cause an increase in 1α‐hydroxylase activity and a decrease in 24-hydroxylase.^70,71^ Nephrocalcinosis and hypercalciuria are common in individuals with IH2; patients with this disorder can be successfully treated with oral phosphate supplemetation,^71^ and we predict that phosphate supplementation may have therapeutic efficacy in individuals with recurrent kidney stone disease.

This study has a number of limitations; using White British ethnicity participant data from the UK Biobank and European ancestry data from the FinnGen study may limit the applicability of our findings across more diverse populations, and despite limited evidence of violations of MR assumptions, bias may still exist with significant heterogeneity identified by the Cochrane Q test in MR analyses. It is plausible that variants included in genetic instruments may affect behaviors, such as fluid ingestion and energy intake, affecting risk of kidney stone formation.^72^ Moreover, some analyses may be underpowered; for example, variability in 24-hour urinary excretion values may explain why higher WHR and BMI were not found to affect urinary calcium excretion. Furthermore, the incidence and prevalence of kidney stone disease in the UK Biobank and FinnGen cohorts are lower than would be expected,^1^ likely as a result of incomplete case ascertainment, and we were unable to ascertain the effects of exposures including interleukin-6 concentrations, Homeostatic Model Assessment for Insulin Resistance, and Homeostatic Model Assessment for Beta Cell Function, on risk of kidney stone disease because of a lack of a suitable number of genetic instruments.

In summary, this study indicates that higher central and general adiposity are independent causal risk factors for kidney stone disease and reveals a novel pathogenic mechanism, demonstrating that higher central adiposity increases serum calcium concentrations to increase risk of kidney stone disease. We predict that medications targeting adipose depots may have effects on calcium homeostasis and utility to prevent kidney formation.

## Supplementary Material

## Supplemental Material

Supplementary Methods.

Supplementary Table 1. Inclusion criteria for observational analyses.

Supplementary Table 2. Exclusion criteria for observational and genome-wide association analyses.

Supplementary Table 3. Inclusion criteria for genome-wide association analyses.

Supplementary Table 4. GWAS data sources for exposure and outcome variables in Mendelian randomization and association analyses.

Supplementary Table 5. All exposure–outcome pairs analyzed using Mendelian randomization.

Supplementary Table 6. UK Biobank Study Population for genome-wide association studies.

Supplementary Table 7. Single-nucleotide polymorphisms significantly associated with kidney stone disease from combined-sex genome-wide association study in the UK Biobank.

Supplementary Table 8. Single-nucleotide polymorphisms significantly associated with kidney stone disease from male sex genome-wide association study in the UK Biobank.

Supplementary Table 9. Single-nucleotide polymorphisms significantly associated with kidney stone disease from female sex genome-wide association study in the UK Biobank.

Supplementary Table 10. Gene set enrichment analyses from UK Biobank-FinnGen meta-analysis executed in MAGMA 18.

Supplementary Table 11. Significantly enriched gene expression in GTEx v8 kidney cortex tissue from UK Biobank-FinnGen meta-analysis

Supplementary Table 12. Heritability estimates and genetic correlation of kidney stone disease GWAS with GWAS of measures of adiposity.

Supplementary Table 13. Univariable Mendelian randomization analyses in UK Biobank.

Supplementary Table 14. Univariable Mendelian randomization analyses on risk of kidney stone disease in FinnGen study.

Supplementary Table 15. Steiger test for directionality of instrumental variables used in Mendelian randomization.

Supplementary Table 16. Sensitivity analyses—Mendelian randomization analyses results after applying Steiger filtering to significant results in univariable Mendelian randomization.

Supplementary Table 17. Multivariable Mendelian randomization analyses in UK Biobank.

Supplementary Table 18. Multivariable Mendelian randomization analyses in FinnGen study.

Supplementary Table 19. Observational associations of the effects of waist-to-hip ratio on albumin-adjusted serum calcium concentrations in UK Biobank.

Supplementary Table 20. Summary of Mendelian randomization estimates for type 2 diabetes and kidney stone disease.

Supplementary Figure 1. Methods for mediation Mendelian randomization.

Supplementary Figure 2. Derivation of UK Biobank cohort for observational analyses.

Supplementary Figure 3. Hazard ratio (HR) for incident kidney stone disease (KSD) and 95% confidence interval (CI) per standard deviation (SD) change in body mass index (BMI), waist-to-hip ratio (WHR), and waist circumference (WC) in observational analysis in the UK Biobank.

Supplementary Figure 4. Results of combined sex genome-wide association study (GWAS) in kidney stone disease (KSD) in UK Biobank.

Supplementary Figure 5. Results of sex-specific genome-wide association study in kidney stone disease in the UK Biobank.

Supplementary Figure 6. Sex-specific odds ratios, 95% confidence intervals, and *P* values for significant hits in genome-wide association studies in UK Biobank of male patients or female participants.

Supplementary Figure 7. Differential gene expression across 54 tissue types in GTEx v8.

Supplementary Figure 8. Significant relationships identified on Mendelian randomization.

Supplementary Figure 9. Sex-specific Mendelian randomization estimates for adiposity-related exposure variables on risk of kidney stone disease.

Supplementary Figure 10. Mendelian randomization leave-one-out analyses for the effects of serum phosphate concentrations on risk of kidney stone disease in UK Biobank-FinnGen meta-analysis.

Supplementary Figure 11. Mendelian randomization leave-one-out analyses for the effects of serum phosphate concentrations on risk of kidney stone disease in FinnGen.

Supplemental Data. All leave-one-out analyses.
