# Supplementary material for: Central Adiposity Increases Risk of Kidney Stone Disease through Effects on Serum Calcium Concentrations
Source: J Am Soc Nephrol. 2023 Oct 3;34(12):1991–2011. doi: 10.1681/ASN.0000000000000238 (PMC10703081; doi:10.1681/ASN.0000000000000238)
Supplement: Supplementary file 1 [file jasn-34-1991-s001.pdf]

Supplementary information

This appendix has been provided by the authors to give additional information about their work

Supplement to:

Central adiposity increases risk of kidney stone disease via effects on serum calcium concentrations

Contents

Supplementary Methods: 4

Supplementary Table 1: Inclusion criteria for observational analyses 5

Supplementary Table 2: Exclusion criteria for observational and genome-wide association analyses 7

Supplementary Table 3: Inclusion criteria for genome-wide association analyses 8

Supplementary Table 4: GWAS data sources for exposure and outcome variables in Mendelian randomisation and association analyses 10

Supplementary Table 5: All exposure-outcome pairs analysed using Mendelian randomisation. 12

Supplementary Table 6: UK Biobank Study Population for genome-wide association studies. 16

Supplementary Table 7: Single nucleotide polymorphisms significantly associated with kidney stone disease from combined sex genome-wide association study in the UK Biobank 16

Supplementary Table 8: Single nucleotide polymorphisms significantly associated with kidney stone disease from male sex genome-wide association study in the UK Biobank 17

Supplementary Table 9: Single nucleotide polymorphisms significantly associated with kidney stone disease from female sex genome-wide association study in the UK Biobank 18

Supplementary Table 10: Gene set enrichment analyses from UK Biobank-FinnGen meta-analysis executed in MAGMA18

Supplementary Table 11: Significantly enriched gene expression in GTEx v8 kidney cortex tissue from UK Biobank-FinnGen meta-analysis 19

Supplementary Table 12: Heritability estimates and genetic correlation of kidney stone disease GWAS with GWAS of measures of adiposity 19

Supplementary Table 13: Univariable Mendelian randomization analyses in UK Biobank 20

Supplementary Table 14: Univariable Mendelian randomization analyses on risk of kidney stone disease in FinnGen study 23

Supplementary Table 15: Steiger test for directionality of instrumental variables used in Mendelian randomisation 25

Supplementary Table 16: Sensitivity analyses- Mendelian randomization analyses results after applying Steiger filtering to significant results in univariable Mendelian randomisation 27

Supplementary Table 17: Multivariable Mendelian randomization analyses in UK Biobank 29

Supplementary Table 18: Multivariable Mendelian randomization analyses in FinnGen study 31

Supplementary Table 19: Observational associations of the effects of waist-to-hip ratio on albumin-adjusted serum calcium concentrations in UK Biobank 32

Supplementary Table 20: Summary of Mendelian randomisation estimates for type 2 diabetes and kidney stone disease. 32

Supplementary Figure 1: Methods for mediation Mendelian randomisation 33

Supplementary Figure 2: Derivation of UK Biobank cohort for observational analyses 34

Supplementary Figure 3: Hazard ratio (HR) for incident kidney stone disease (KSD) and 95% confidence interval (CI) per standard deviation (SD) change in body mass index (BMI), waist-to-hip ratio (WHR) and waist circumference (WC) in observational analysis in the UK Biobank 35

Supplementary Figure 4: Results of combined sex genome-wide association study (GWAS) in kidney stone disease (KSD) in UK Biobank 36

Supplementary Figure 5: Results of sex-specific genome wide association study in kidney stone disease in the UK Biobank 37

Supplementary Figure 6: Sex-specific odds ratios, 95% confidence intervals and p values for significant hits in genome wide association studies in UK Biobank of males or females. 38

Supplementary Figure 7: Differential gene expression across 54 tissue types in GTEx v8. 39

Supplementary Figure 8: Significant relationships identified on Mendelian randomisation 43

Supplementary Figure 9. Sex-specific Mendelian randomisation estimates for adiposity-related exposure variables on risk of kidney stone disease. 44

Supplementary Figure 10. Mendelian randomisation leave-one-out analyses for the effects of serum phosphate concentrations on risk of kidney stone disease in UK Biobank-FinnGen meta-analysis. 46

Supplementary Figure 11. Mendelian randomisation leave-one-out analyses for the effects of serum phosphate concentrations on risk of kidney stone disease in FinnGen. **48**

Supplemental Data. All leave-one-out analyses

Supplementary Methods:

Sample, individual, and single nucleotide polymorphism quality control criteria prior to genome wide analysis of kidney stone disease in the UK Biobank

Sample-level quality control excluded individuals with one or more of: call rate <98%; discrepancy between genetic-sex (data-field 22001) and self-reported sex (data-field 21); sex chromosome aneuploidy (data-field 22019); heterozygosity >3SD from the mean (UKB PCA-adjusted heterozygosity values, data-field 20004). Individuals not of white British ancestry were excluded (principal component analysis and self-reported ethnicity (data-field 22006). SNPs with Hardy–Weinberg equilibrium $p<10^{-4}$, <98% call rate, and minor allele frequency (MAF) <1% were excluded in SNP-level quality control. Single nucleotide polymorphisms (SNPs) with a call rate <90% were omitted.

Genome-wide association studies of albumin-adjusted serum calcium and phosphate concentrations (Fadil Hannan, Anubha Mahajan, personal communication, doi: https://doi.org/10.25446/oxford.23735709.v1):

Serum albumin-adjusted calcium concentrations for UK Biobank participants were derived using the following equation: *adjusted calcium (mmol/L)=total Calcium(mmol/L) + 0.0177 *(46.3 – albumin (g/L))*. Data from participants with eGFR(MDRD) <60ml/min/1.73m^2 and 25-OH vitamin D concentrations <30nmol/L were excluded from association analyses for both serum phosphate concentrations and serum albumin-adjusted calcium concentrations. Analyses were performed using genotyped and imputed variants from the UK Biobank. Genotyping was undertaken using UK-BiLEVE and UK-Biobank Axiom Arrays, as described in the "Methods" section of the main text. Phenotypes were inverse normalised with additional adjustments for array, age, and sex. Analyses were undertaken in individuals of European ancestry using BOLT-LMM to account for population sub-structure and cryptic relatedness. Imputed SNPs of minor allele frequency (MAF) <1% and of imputation quality score <0.3 were excluded from analyses. Lead SNPs were identified from unconditional meta-analyses and loci defined as ±500kb surrounding each SNP. Overlapping loci were merged as one locus. GCTA was used to perform a stepwise model selection procedure to select independently-associated SNPs within each 1Mb region of significance $p<5x10^{-9}$. Directly genotyped variants underwent stringent quality control checks, including call rate per array, manual cluster plot checks and status in Gnomad. Only variants with MAF <1% and the coding or loss-of-function annotations of "missense variant", "stop gain", "frameshift variant", "splice acceptor variant", "splice donor variant", "splice region variant", "start lost", or "stop lost" were included. A significance threshold of $p<5x10^{-6}$ was used to identify directly genotyped SNPs with significant associations with each phenotype.

Supplementary Table 1: Inclusion criteria for observational analyses

International Classification of Diseases Codes (ICD)		OPCS Classification of Interventions and Procedures Codes (OPCS)		Self-reported operation codes		Self-reported condition		Death	
ICD9-5920	Calculus of kidney	OPCS3-5631	Removal of renal calculus: nephrolithomy	1197	Percutaneous/open kidney stone surgery/lithotripsy	1197	Kidney stone/ureter stone/bladder stone	N20.0	Calculus of kidney
ICD9-5921	Calculus of ureter	OPCS3-5632	Removal of renal calculus: pyelolithotomy					N20.1	Calculus of ureter
ICD9-5929	Urinary calculus, unspecified	OPCS3-5633	Removal of renal calculus: removal without incision					N20.2	Calculus of kidney with calculus of ureter
ICD9-7880	Renal colic	OPCS4-M06.1	Open removal of calculus from kidney					N20.9	Urinary calculus, unspecified
ICD10-N20.0	Calculus of kidney	OPCS4-M09.1	Endoscopic ultrasound fragmentation of calculus of kidney					N23	Unspecified Renal Colic
ICD10-N20.1	Calculus of ureter	OPCS4-M09.2	Endoscopic electrohydraulic shock wave fragmentation of calculus of kidney						
ICD10-N20.2	Calculus of kidney with calculus of ureter	OPCS4-M09.3	Endoscopic laser fragmentation of calculus of kidney						
ICD10-N20.9	Urinary calculus, unspecified	OPCS4-M09.4	Endoscopic extraction of calculus of kidney						
ICD10-N23	Unspecified Renal Colic	OPCS4-M09.8	Other specified therapeutic endoscopic operations on calculus of kidney,						
		OPCS4-M09.9	Unspecified therapeutic endoscopic operations on calculus of kidney						
		OPCS4-M14.1	Extracorporeal shock wave lithotripsy of calculus of kidney						
		OPCS4-M14.8	Other specified extracorporeal fragmentation of calculus of kidney						
		OPCS4-M14.9	Unspecified extracorporeal fragmentation of calculus of kidney						
		OPCS4-M16.4	Percutaneous nephrolithotomy						
		OPCS4-M27.1	Ureteroscopic laser fragmentation of calculus of ureter						
		OPCS4-M27.2	Ureteroscopic fragmentation of calculus of ureter						
		OPCS4-M27.3	Ureteroscopic extraction of calculus of ureter						
		OPCS4-M28.1	Endoscopic laser fragmentation of calculus of ureter						

OPCS4-M28.2	Endoscopic fragmentation of calculus of ureter
OPCS4-M28.3	Endoscopic extraction of calculus of ureter
OPCS4-M28.4	Endoscopic catheter drainage of calculus of ureter
OPCS4-M28.5	Endoscopic drainage of calculus of ureter by dilation of ureter
OPCS4-M28.8	Other specified other endoscopic removal of calculus from ureter
OPCS4-M28.9	Unspecified other endoscopic removal of calculus from ureter
OPCS4-M31.1	Extracorporeal shock wave lithotripsy of calculus of ureter
OPCS4-M31.8	Other specified extracorporeal fragmentation of calculus of ureter
OPCS4-M31.9	Unspecified extracorporeal fragmentation of calculus of ureter
OPCS4-M26.1	Nephroscopic laser fragmentation of calculus of ureter
OPCS4-M26.2	Nephroscopic fragmentation of calculus of ureter NEC
OPCS4-M26.3	Nephroscopic extraction of calculus of ureter

Supplementary Table 2: Exclusion criteria for observational and genome-wide association analyses

ICD-10 Codes		ICD9	OPCS 4 codes		OPCS3
E26.81	Bartter syndrome	255.13	M39.1	Open removal of calculus from bladder	600.2
E72.0	Disorders of amino acid transport	270	M44.2	Endoscopic extraction of calculus of bladder	
E21.0	Hyperparathyroidism	252	M67.4	Endoscopic removal of calculus from prostate	
E21.1	Hyperparathyroidism	252.02	M75.8	Open extraction of calculus from urethra	
E21.2	Hyperparathyroidism	252.08	G27.1	Gastric bypass surgery	426.3
E21.3	Hyperparathyroidism		G27.2	Gastric bypass surgery	426
Q61.5	Medullary sponge kidney	753.17	G27.3	Gastric bypass surgery	
N25.8	Type 1 renal tubular acidosis		G27.4	Gastric bypass surgery	
K50	Inflammatory bowel disease	558.9	G27.5	Gastric bypass surgery	
K51	Inflammatory bowel disease		G27.8	Gastric bypass surgery	
K91.2	Postsurgical malabsorption	579.3	G28.1	Gastric bypass surgery	
Q62	Congenital obstructive defects of the renal pelvis and malformations of the ureter	753.29	G28.2	Gastric bypass surgery	
			G28.3	Gastric bypass surgery	
E83.31	Hereditary hypophosphatemic rickets with hypercalciuria and nephrolithiasis, osteoporosis, and hypophosphatemia	275.3	G28.4	Gastric bypass surgery	
			G28.5	Gastric bypass surgery	
E83.42	Familial hypomagnesemia with hypercalciuria and nephrocalcinosis and Familial hypomagnesemia with hypercalciuria and nephrocalcinosis with ocular abnormalities	275.2	G28.8	Gastric bypass surgery	
			G28.9	Gastric bypass surgery	
E74.8	Oxaluria and oxalosis	271.4	G31.1	Gastric bypass surgery	
		271.8	G31.2	Gastric bypass surgery	
N21.0	Calculus in bladder	594	G31.3	Gastric bypass surgery	
		594.1	G31.4	Gastric bypass surgery	
N21.1	Calculus in urethra	594.2	G31.8	Gastric bypass surgery	
N21.8	Other lower urinary tract calculus	594.8	G31.9	Gastric bypass surgery	
N21.9	Calculus of the lower urinary tract	594.9	G31.0	Gastric bypass surgery	
			G32.1	Gastric bypass surgery	
			G32.2	Gastric bypass surgery	
			G32.3	Gastric bypass surgery	
			G32.4	Gastric bypass surgery	
			G32.8	Gastric bypass surgery	
			G32.9	Gastric bypass surgery	
			G32.0	Gastric bypass surgery	
			G33.1	Gastric bypass surgery	
			G33.2	Gastric bypass surgery	
			G33.3	Gastric bypass surgery	
			G33.6	Gastric bypass surgery	
			G33.8	Gastric bypass surgery	
			G33.9	Gastric bypass surgery	
			G33.0	Gastric bypass surgery	

Supplementary Table 3: Inclusion criteria for genome-wide association analyses

**International Classification of Diseases Codes (ICD)**		**OPCS Classification of Interventions and Procedures Codes (OPCS)**		**Self-reported operation code**		**Death**	
ICD9-5920	Calculus of kidney	OPCS3-5631	Removal of renal calculus: nephrolithomy	1197	Percutaneous/open kidney stone surgery/lithotripsy	N20.0	Calculus of kidney
ICD9-5921	Calculus of ureter	OPCS3-5632	Removal of renal calculus: pyelolithotomy			N20.1	Calculus of ureter
ICD9-5929	Urinary calculus, unspecified	OPCS3-5633	Removal of renal calculus: removal without incision			N20.2	Calculus of kidney with calculus of ureter
ICD9-7880	Renal colic	OPCS4-M06.1	Open removal of calculus from kidney			N20.9	Urinary calculus, unspecified
ICD10-N20.0	Calculus of kidney	OPCS4-M09.1	Endoscopic ultrasound fragmentation of calculus of kidney			N23	Unspecified Renal Colic
ICD10-N20.1	Calculus of ureter	OPCS4-M09.2	Endoscopic electrohydraulic shock wave fragmentation of calculus of kidney				
ICD10-N20.2	Calculus of kidney with calculus of ureter	OPCS4-M09.3	Endoscopic laser fragmentation of calculus of kidney				
ICD10-N20.9	Urinary calculus, unspecified	OPCS4-M09.4	Endoscopic extraction of calculus of kidney				
ICD10-N23	Unspecified Renal Colic	OPCS4-M09.8	Other specified therapeutic endoscopic operations on calculus of kidney,				
		OPCS4-M09.9	Unspecified therapeutic endoscopic operations on calculus of kidney				
		OPCS4-M14.1	Extracorporeal shock wave lithotripsy of calculus of kidney				
		OPCS4-M14.8	Other specified extracorporeal fragmentation of calculus of kidney				
		OPCS4-M14.9	Unspecified extracorporeal fragmentation of calculus of kidney				
		OPCS4-M16.4	Percutaneous nephrolithotomy				
		OPCS4-M26.1	Nephroscopic laser fragmentation of calculus of ureter				
		OPCS4-M26.2	Nephroscopic fragmentation of calculus of ureter NEC				
		OPCS4-M26.3	Nephroscopic extraction of calculus of ureter				
		OPCS4-M27.1	Ureteroscopic laser fragmentation of calculus of ureter				
		OPCS4-M27.2	Ureteroscopic fragmentation of calculus of ureter				

OPCS4-M27.3	Ureteroscopic extraction of calculus of ureter
OPCS4-M28.1	Endoscopic laser fragmentation of calculus of ureter
OPCS4-M28.2	Endoscopic fragmentation of calculus of ureter
OPCS4-M28.3	Endoscopic extraction of calculus of ureter
OPCS4-M28.4	Endoscopic catheter drainage of calculus of ureter
OPCS4-M28.5	Endoscopic drainage of calculus of ureter by dilation of ureter
OPCS4-M28.8	Other specified other endoscopic removal of calculus from ureter
OPCS4-M28.9	Unspecified other endoscopic removal of calculus from ureter
OPCS4-M31.1	Extracorporeal shock wave lithotripsy of calculus of ureter
OPCS4-M31.8	Other specified extracorporeal fragmentation of calculus of ureter
OPCS4-M31.9	Unspecified extracorporeal fragmentation of calculus of ureter

Supplementary Table 4: GWAS data sources for exposure and outcome variables in Mendelian randomisation and association analyses

Parameter	Source	Sample population	Mean R^2 (SD) of exposure instrumental variables	F statistic of exposure instrumental variables
2-hour glucose tolerance	Chen et al [1]	Meta-analysis of 24 of European ancestry	1.97x10-3 (1.07x10-3)	33.00
24h urine calcium	Gary Curhan	2014 participants, NHSI/NHSII/HPFS, personal communication	NA	NA
25-OH Vitamin D	Revez et al[2]	UK Biobank- European ancestry	4.31x10-4 (1.83x10-3)	3.76
Adjusted serum calcium*	Fadil Hannan, Anubha Mahajan et al[3]	UK Biobank- European ancestry, personal communication See supplementary methods page 4	3.18x10-4 (5.54x10-4)	0.53
APO-B	Richardson et al [4]	UK Biobank- European ancestry	4.52x10-4 (1.27x10-3)	1.13
ASAT, GFAT, VAT	Agrawal et al[5]	GWAS of 38,965 individuals in the UK Biobank, 87% of white British ancestry	ASAT: 1.12x10-3 (7.27x10-4)<br>GFAT: 9.47x10-4 (2.10x10-4)<br>VAT: 1.02x10-3 (1.24x10-4)<br>ASAT/GFAT: 1.00x10-3 (4.00x10-4)<br>VAT/ASAT: 1.01x10-3 (4.46x10-4)<br>VAT/GSAT: 1.01x10-3 (3.36x10-4)	8.74<br>2.17<br>9.94<br>2.78<br>1.97<br>2.07
BMD	Morris et al[6]	GWAS of 426,824 individuals in the UK Biobank- European ancestry	2.86x10-4 (7.12x10-4)	0.15
BMI	Pulit et al [7]	Meta-analysis of UK Biobank GWAS and GIANT consortium studies. European ancestry	1.12x10-3 (7.27x10-4)	1.53
CRP	Ligthart et al [8]	Meta-analysis of HapMap (204,402 individuals from 78 studies) and 1000 Genomes (148,164 individuals from 49 studies), European ancestry	1.27x10-3 (3.04x10-3)	8.30
Fasting glucose	Chen et al [1]	Meta-analysis of 71 studies including European ancestry	1.98x10-4 (3.96x10-4)	0.62
Fasting insulin	Chen et al [1]	Meta-analysis of 60 studies of European ancestry	9.74x10-5 (5.91x10-5)	0.51
HbA1c	Chen et al [1]	Meta-analysis of 41 studies of European ancestry	8.46x10-5 (1.18x10-4)	0.21
HDL	Richardson et al [4]	UK Biobank- European ancestry	2.63x10-4 (6.46x10-4)	0.33
Hypertension	Evangelou et al [9]	Meta-analysis of UK Biobank GWAS and exome data in 8 studies, European ancestry	SBP: 0.035 (0.034)<br>DBP: 0.012 (0.013)	62.41<br>21.00
KSD	GWAS and meta-analysis presented in this manuscript	UK Biobank- European ancestry	UK Biobank GWAS: 2.76x10-6 (9.69x10-7)<br>Meta-analysis: 5.13x10-3 (4.34x10-3)	0.08
LDL	Richardson et al [4]	UK Biobank- European ancestry	3.73x10-4 (7.98x10-4)	1.07
Serum phosphate	Fadil Hannan, Anubha Mahajan et al[3]	UK Biobank- European ancestry, personal communication See supplementary methods page 4	2.76x10-6 (9.69x10-7)	0.01
T2D	Vijkovic et al [10]	Multi-ancestry meta-analysis of 228,499 cases and 1,178,783 controls in the Million Veteran Program (MVP), DIAMANTE, Biobank Japan, and other studies. European ancestry data used.	8.42x10-4 (1.80x10-3)	3.20
Triglycerides	Richardson et al [4]	UK Biobank- European ancestry	2.94x10-4 (6.50x10-4)	0.46
Urate	Neale lab[11]	UK Biobank- European ancestry	4.31x10-4 (1.83x10-3)	0.78
WC	Shungin et al[12]	GWAS meta-analyses of traits related to waist and hip circumferences in up to 224,459 individuals. European ancestry.	1.00x10-3 (4.00x10-4)	5.62
WHR	Pulit et al [7]	Meta-analysis of UK Biobank GWAS and GIANT consortium studies. European ancestry	1.01x10-4 (7.09x10-5)	0.25

APO-B= apolipoprotein-B, BMD= bone mineral density, BMI= body mass index, CRP= C-reactive protein, GWAS= genome-wide association study, HDL= high-density lipoprotein, KSD= kidney stone disease, LDL= low-density lipoprotein, sIL-6R= serum IL-6 receptor, T2D= type 2 diabetes, WC= waist circumference, WHR= waist-to-hip ratio. *Total calcium + 0.0177x(46.3-albumin).

1. Chen J, Spracklen CN, Marenne G, et al. The trans-ancestral genomic architecture of glycemic traits. *Nature genetics*. 2021;53(6):840-860.
2. Revez JA, Lin T, Qiao Z, et al. Genome-wide association study identifies 143 loci associated with 25 hydroxyvitamin D concentration. *Nat Commun*. Apr 2 2020;11(1):1647. doi:10.1038/s41467-020-15421-7
3. Hannan F, Mahajan A. Summary statistics for mineral metabolism GWAS. University of Oxford; 2023.

4. Richardson TG, Sanderson E, Palmer TM, et al. Evaluating the relationship between circulating lipoprotein lipids and apolipoproteins with risk of coronary heart disease: A multivariable Mendelian randomisation analysis. *PLoS medicine*. Mar 2020;17(3):e1003062. doi:10.1371/journal.pmed.1003062
5. Agrawal S, Wang M, Klarqvist MDR, et al. Inherited basis of visceral, abdominal subcutaneous and gluteofemoral fat depots. *Nat Commun*. Jun 30 2022;13(1):3771. doi:10.1038/s41467-022-30931-2
6. Morris JA, Kemp JP, Youlten SE, et al. An atlas of genetic influences on osteoporosis in humans and mice. *Nature genetics*. Feb 2019;51(2):258-266. doi:10.1038/s41588-018-0302-x
7. Pulit SL, Stoneman C, Morris AP, et al. Meta-analysis of genome-wide association studies for body fat distribution in 694 649 individuals of European ancestry. *Human molecular genetics*. Jan 1 2019;28(1):166-174. doi:10.1093/hmg/ddy327
8. Ligthart S, Vaez A, Vosa U, et al. Genome Analyses of >200,000 Individuals Identify 58 Loci for Chronic Inflammation and Highlight Pathways that Link Inflammation and Complex Disorders. *American journal of human genetics*. Nov 1 2018;103(5):691-706. doi:10.1016/j.ajhg.2018.09.009
9. Evangelou E, Warren HR, Mosen-Ansorena D, et al. Genetic analysis of over 1 million people identifies 535 new loci associated with blood pressure traits. *Nature genetics*. Oct 2018;50(10):1412-1425. doi:10.1038/s41588-018-0205-x
10. Vujkovic M, Keaton JM, Lynch JA, et al. Discovery of 318 new risk loci for type 2 diabetes and related vascular outcomes among 1.4 million participants in a multi-ancestry meta-analysis. *Nature genetics*. Jul 2020;52(7):680-691. doi:10.1038/s41588-020-0637-y
11. Neale lab. Urate- metabolite GWAS. 22.02.2023, 2023. Accessed 22.02.2023, 2023. http://www.nealelab.is/
12. Shungin D, Winkler TW, Croteau-Chonka DC, et al. New genetic loci link adipose and insulin biology to body fat distribution. *Nature*. Feb 12 2015;518(7538):187-196. doi:10.1038/nature14132

Supplementary Table 5: All exposure-outcome pairs analysed using Mendelian randomisation.

Group	Exposure	Outcome
Adiposity	Abdominal subcutaneous adipose tissue	Kidney stone disease (UK Biobank)
Adiposity	Abdominal subcutaneous adipose tissue	Kidney stone disease (FinnGen)
Adiposity	Abdominal subcutaneous adipose tissue	Kidney stone disease (Meta-analysis)
Adiposity	Abdominal subcutaneous adipose tissue/ Gluteofemoral adipose tissue	Kidney stone disease (UK Biobank)
Adiposity	Abdominal subcutaneous adipose tissue/ Gluteofemoral adipose tissue	Kidney stone disease (FinnGen)
Adiposity	Abdominal subcutaneous adipose tissue/ Gluteofemoral adipose tissue	Kidney stone disease (Meta-analysis)
Adiposity	Body mass index	Heel bone mineral density
Adiposity	Body mass index	Kidney stone disease (UK Biobank Males)
Adiposity	Body mass index	Kidney stone disease (UK Biobank Females)
Adiposity	Body mass index	Kidney stone disease (UK Biobank)
Adiposity	Body mass index	Kidney stone disease (FinnGen)
Adiposity	Body mass index	Kidney stone disease (Meta-analysis)
Adiposity	Body mass index	Serum 25-OH vitamin D concentration
Adiposity	Body mass index	Serum calcium concentration
Adiposity	Body mass index	Serum phosphate concentration
Adiposity	Body mass index	Urine calcium excretion
Adiposity	Body mass index	Waist-to-hip ratio
Adiposity	Gluteofemoral adipose tissue	Kidney stone disease (UK Biobank)
Adiposity	Gluteofemoral adipose tissue	Kidney stone disease (FinnGen)
Adiposity	Gluteofemoral adipose tissue	Kidney stone disease (Meta-analysis)
Adiposity	Kidney stone disease (UK Biobank GWAS)	Body mass index
Adiposity	Kidney stone disease (UK Biobank GWAS)	Waist circumference
Adiposity	Kidney stone disease (UK Biobank GWAS)	Waist-to-hip ratio
Adiposity	Kidney stone disease (UK Biobank-FinnGen Meta-analysis)	Body mass index
Adiposity	Kidney stone disease (UK Biobank-FinnGen Meta-analysis)	Waist circumference
Adiposity	Kidney stone disease (UK Biobank-FinnGen Meta-analysis)	Waist-to-hip ratio
Adiposity	Visceral adipose tissue	Kidney stone disease (UK Biobank)
Adiposity	Visceral adipose tissue	Kidney stone disease (FinnGen)
Adiposity	Visceral adipose tissue	Kidney stone disease (Meta-analysis)
Adiposity	Visceral adipose tissue/ Abdominal subcutaneous adipose tissue	Kidney stone disease (UK Biobank)
Adiposity	Visceral adipose tissue/ Abdominal subcutaneous adipose tissue	Kidney stone disease (FinnGen)

Adiposity	Visceral adipose tissue/ Abdominal subcutaneous adipose tissue	Kidney stone disease (Meta-analysis)
Adiposity	Visceral adipose tissue/ Gluteofemoral adipose tissue	Kidney stone disease (UK Biobank)
Adiposity	Visceral adipose tissue/ Gluteofemoral adipose tissue	Kidney stone disease (FinnGen)
Adiposity	Visceral adipose tissue/ Gluteofemoral adipose tissue	Kidney stone disease (Meta-analysis)
Adiposity	Waist circumference	Kidney stone disease (UK Biobank Males)
Adiposity	Waist circumference	Kidney stone disease (UK Biobank Females)
Adiposity	Waist circumference	Kidney stone disease (UK Biobank)
Adiposity	Waist circumference	Kidney stone disease (FinnGen)
Adiposity	Waist circumference	Kidney stone disease (Meta-analysis)
Adiposity	Waist circumference	Serum calcium concentration
Adiposity	Waist-to-hip ratio	24 hour urine calcium excretion
Adiposity	Waist-to-hip ratio	Body mass index
Adiposity	Waist-to-hip ratio	Heel bone mineral density
Adiposity	Waist-to-hip ratio	Kidney stone disease (UK Biobank Males)
Adiposity	Waist-to-hip ratio	Kidney stone disease (UK Biobank Females)
Adiposity	Waist-to-hip ratio	Kidney stone disease (UK Biobank)
Adiposity	Waist-to-hip ratio	Kidney stone disease (FinnGen)
Adiposity	Waist-to-hip ratio	Kidney stone disease (Meta-analysis)
Adiposity	Waist-to-hip ratio	Serum 25-OH vitamin D concentration
Adiposity	Waist-to-hip ratio	Serum calcium concentration
Adiposity	Waist-to-hip ratio	Serum phosphate concentration
Biochemistry	Heel bone mineral density	Kidney stone disease (UK Biobank)
Biochemistry	Heel bone mineral density	Kidney stone disease (FinnGen)
Biochemistry	Heel bone mineral density	Kidney stone disease (Meta-analysis)
Biochemistry	Heel bone mineral density	Serum calcium concentration
Biochemistry	Kidney stone disease (Meta-analysis)	Serum calcium concentration
Biochemistry	Kidney stone disease (UK Biobank)	Serum calcium concentration
Biochemistry	Serum 25-OH vitamin D concentration	Kidney stone disease (UK Biobank)
Biochemistry	Serum 25-OH vitamin D concentration	Kidney stone disease (FinnGen)
Biochemistry	Serum 25-OH vitamin D concentration	Kidney stone disease (Meta-analysis)
Biochemistry	Serum calcium concentration	Body mass index
Biochemistry	Serum calcium concentration	Kidney stone disease (UK Biobank)
Biochemistry	Serum calcium concentration	Kidney stone disease (FinnGen)
Biochemistry	Serum calcium concentration	Kidney stone disease (Meta-analysis)

Biochemistry	Serum calcium concentration	Serum 25-OH vitamin D concentration
Biochemistry	Serum calcium concentration	Waist-to-hip ratio
Biochemistry	Serum phosphate concentration	Body mass index
Biochemistry	Serum phosphate concentration	Kidney stone disease (UK Biobank)
Biochemistry	Serum phosphate concentration	Kidney stone disease (FinnGen)
Biochemistry	Serum phosphate concentration	Kidney stone disease (Meta-analysis)
Biochemistry	Urate	Kidney stone disease (UK Biobank)
Biochemistry	Urate	Kidney stone disease (FinnGen)
Biochemistry	Urate	Kidney stone disease (Meta-analysis)
Metabolic Syndrome	2 hour glucose tolerance	Kidney stone disease (UK Biobank)
Metabolic Syndrome	2 hour glucose tolerance	Kidney stone disease (FinnGen)
Metabolic Syndrome	2 hour glucose tolerance	Kidney stone disease (Meta-analysis)
Metabolic Syndrome	Diastolic blood pressure	Kidney stone disease (UK Biobank)
Metabolic Syndrome	Diastolic blood pressure	Kidney stone disease (FinnGen)
Metabolic Syndrome	Diastolic blood pressure	Kidney stone disease (Meta-analysis)
Metabolic Syndrome	Fasting glucose adjusted for Body mass index	Kidney stone disease (UK Biobank)
Metabolic Syndrome	Fasting glucose adjusted for Body mass index	Kidney stone disease (FinnGen)
Metabolic Syndrome	Fasting glucose adjusted for Body mass index	Kidney stone disease (Meta-analysis)
Metabolic Syndrome	Fasting insulin adjusted for Body mass index	Kidney stone disease (UK Biobank)
Metabolic Syndrome	Fasting insulin adjusted for Body mass index	Kidney stone disease (FinnGen)
Metabolic Syndrome	Fasting insulin adjusted for Body mass index	Kidney stone disease (Meta-analysis)
Metabolic Syndrome	HbA1c	Kidney stone disease (UK Biobank)
Metabolic Syndrome	HbA1c	Kidney stone disease (FinnGen)
Metabolic Syndrome	HbA1c	Kidney stone disease (Meta-analysis)
Metabolic Syndrome	Systolic blood pressure	Kidney stone disease (UK Biobank)
Metabolic Syndrome	Systolic blood pressure	Kidney stone disease (FinnGen)
Metabolic Syndrome	Systolic blood pressure	Kidney stone disease (Meta-analysis)
Metabolic Syndrome	Serum high density cholesterol concentration	Kidney stone disease (UK Biobank)
Metabolic Syndrome	Serum high density cholesterol concentration	Kidney stone disease (FinnGen)
Metabolic Syndrome	Serum high density cholesterol concentration	Kidney stone disease (Meta-analysis)
Metabolic Syndrome	Serum low density cholesterol concentration	Kidney stone disease (UK Biobank)
Metabolic Syndrome	Serum low density cholesterol concentration	Kidney stone disease (FinnGen)

Metabolic Syndrome	Serum low density cholesterol concentration	Kidney stone disease (Meta-analysis)
Metabolic Syndrome	Serum triglyceride concentration	Kidney stone disease (UK Biobank)
Metabolic Syndrome	Serum triglyceride concentration	Kidney stone disease (FinnGen)
Metabolic Syndrome	Serum triglyceride concentration	Kidney stone disease (Meta-analysis)
Metabolic Syndrome	Type 2 diabetes	Kidney stone disease (UK Biobank)
Metabolic Syndrome	Type 2 diabetes	Kidney stone disease (FinnGen)
Metabolic Syndrome	Type 2 diabetes	Kidney stone disease (Meta-analysis)
Systemic Inflammation	Apolipoprotein-B	Kidney stone disease (UK Biobank)
Systemic Inflammation	Apolipoprotein-B	Kidney stone disease (FinnGen)
Systemic Inflammation	Apolipoprotein-B	Kidney stone disease (Meta-analysis)
Systemic Inflammation	C reactive protein	Kidney stone disease (UK Biobank)
Systemic Inflammation	C reactive protein	Kidney stone disease (FinnGen)
Systemic Inflammation	C reactive protein	Kidney stone disease (Meta-analysis)

Supplementary Table 6: UK Biobank Study Population for genome-wide association studies.

Cohort	Number of Samples	Female (%)	Male (%)
Kidney stone	8,504	2,871 (33.8)	5,633 (66.2)
Control	388,819	212,081 (54.5)	176,738 (45.5)

Supplementary Table 7: Single nucleotide polymorphisms significantly associated with kidney stone disease from combined sex genome-wide association study in the UK Biobank

Chr	Pos	rsID	Annotation	EA	NEA	EAF	Info	OR (95% CI)	P	Candidate Gene
1	21836204	rs77362499	Intronic	G	C	0.13	0.995	1.24 (1.18-1.30)	$2.3x10^{-17}$	*ALPL*
1	21893344	rs1256332	Intronic	A	C	0.18	0.995	1.17 (1.12-1.22)	$5.4x10^{-14}$	*ALPL*
2	234296444	rs838717	Intronic	G	A	0.46	0.995	1.10 (1.06-1.13)	$2.9x10^{-9}$	*DGKD*
5	176798040	rs56235845	Intronic	G	T	0.36	0.986	1.16 (1.13-1.20)	$5.0x10^{-20}$	*SLC34A1*
6	39146230	rs1155347	Intergenic	C	T	0.24	0.975	1.14 (1.10-1.18)	$3.8x10^{-12}$	*KCNK5*
6	134205092	rs969282	Intronic	G	A	0.48	0.996	1.09 (1.06-1.12)	$3.6x10^{-8}$	*SLC2A12*
6	160611103	rs78693187	Intronic	C	T	0.04	0.997	1.33 (1.21-1.45)	$5.7x10^{-10}$	*SLC22A2*
7	27653207	rs7790498	Intronic	A	G	0.30	0.999	1.12 (1.08-1.16)	$1.10x10^{-11}$	*HIBADH*
7	142605221	rs4252512	Intergenic	C	T	0.02	0.993	1.44 (1.28-1.61)	$6.5x10^{-10}$	*TRPV5*
13	42674601	rs1182959	Intronic	A	G	0.20	0.985	1.15 (1.10-1.19)	$2.1x10^{-11}$	*DGKH*
15	53997089	rs578595	Intronic	C	A	0.48	0.996	1.10 (1.06-1.13)	$4.2x10^{-9}$	*WDR72*
15	85520329	rs12439802	Intergenic	G	C	0.16	1	1.13 (1.09-1.18)	$5.5x10^{-9}$	*SLC28A1*
16	20392332	rs77924615	Intronic	A	G	0.22	0.980	1.16 (1.12-1.20)	$5.6x10^{-14}$	*UMOD*
20	52731402	rs6127099	Intergenic	A	T	0.75	0.949	1.14 (1.10-1.18)	$2.8x10^{-14}$	*CYP24A1*
21	37818871	rs2776288	Intronic	A	G	0.66	0.988	1.16 (1.12-1.20)	$1.7x10^{-20}$	*CLDN14*
22	23410918	rs13054904	Intronic	A	T	0.29	0.999	1.15 (1.11-1.19)	$8.3x10^{-16}$	*BCR*

Chr= chromosome; Pos= position based on NCBI Genome Build 37 (hg19); EA= effect allele; EAF= effect allele frequency in kidney stone formers; NEA= Non-effect allele; OR= odds ratio; 95% CI= 95% confidence interval.
>1 independent signals were identified at *ALPL*, *SLC34A1*, *DGKH*, *CYP24A1*, and *CLDN14* loci

Supplementary Table 8: Single nucleotide polymorphisms significantly associated with kidney stone disease from male sex genome-wide association study in the UK Biobank

Chr	Pos	rsID	Annotation	EA	NEA	EAF	Info	OR (95% CI)	P	Candidate Gene
1	21826530	rs115239632	Intergenic	T	C	0.05	1	1.44 (1.31-1.59)	2.7×10^{-13}	*ALPL*
1	21840129	rs869179	Intronic	G	A	0.67	1	1.12 (1.08-1.17)	1.3×10^{-8}	*ALPL*
1	21893344	rs1256332	Intronic	A	C	0.19	1	1.20 (1.14-1.26)	2.0×10^{-12}	*ALPL*
5	176798040	rs56235845	Intronic	G	T	0.36	0.99	1.18 (1.13-1.22)	9.5×10^{-16}	*SLC34A1*
6	160611103	rs78693187	Intronic	C	T	0.04	1	1.32 (1.22-1.53)	2.1×10^{-8}	*SLC22A2*
7	30957616	rs2299905	Intronic	T	A	0.31	0.99	1.12 (1.08-1.17)	4.9×10^{-8}	*AQP1*
7	142605221	rs4252512	Intergenic	C	T	0.02	0.99	1.51 (1.31-1.74)	1.2×10^{-8}	*TRPV5*
13	42758805	rs9590676	Intronic	C	T	0.40	1	1.12 (1.08-1.17)	1.9×10^{-9}	*DGKH*
15	53997089	rs578595	Intronic	C	A	0.49	1	1.11 (1.07-1.16)	2.3×10^{-8}	*WDR72*
16	20392332	rs77924615	Intronic	A	G	0.22	0.98	1.15 (1.10-1.21)	3.2×10^{-9}	*UMOD*
20	52742680	rs111707488	Intergenic	A	AGT	0.32	0.94	1.13 (1.09-1.19)	3.2×10^{-9}	*CYP24A1*
21	37818871	rs2776288	Intronic	A	G	0.66	0.99	1.15 (1.11-1.20)	7.5×10^{-13}	*CLDN14*
22	23410918	rs13054904	Intronic	A	T	0.30	1	1.17 (1.12-1.22)	4.4×10^{-13}	*BCR*

Chr= chromosome; Pos= position based on NCBI Genome Build 37 (hg19); EA= effect allele; EAF= effect allele frequency in kidney stone formers; NEA= Non-effect allele; OR= odds ratio; 95% CI= 95% confidence interval.

Supplementary Table 9: Single nucleotide polymorphisms significantly associated with kidney stone disease from female sex genome-wide association study in the UK Biobank

**Chr**	**Pos**	**rsID**	**Annotation**	**EA**	**NEA**	**EAF**	**Info**	**OR (95% CI)**	**P**	**Candidate Gene**
7	27617940	7:27617940_AT_A	Intronic	AT	A	0.32	0.95	1.18 (1.12-1.26)	$1.5x10^{-8}$	*HIBADH*
20	52731402	rs6127099	Intergenic	A	T	0.75	0.99	1.19 (1.12-1.26)	$1.4x10^{-9}$	*CYP24A1*
21	37818871	rs2776288	Intronic	A	G	0.66	0.99	1.17 (1.12-1.24)	$2.7x10^{-9}$	*CLDN14*

Chr= chromosome; Pos= position based on NCBI Genome Build 37 (hg19); EA= effect allele; EAF= effect allele frequency in kidney stone formers; NEA= Non-effect allele; OR= odds ratio; 95% CI= 95% confidence interval.

Supplementary Table 10: Gene set enrichment analyses from UK Biobank-FinnGen meta-analysis executed in MAGMA

HPO GO Geneset	Number of genes	Beta	SE	P value	Overlapping genes
Hypermagnesemia	7	*1.68*	0.32	5.35x10-8	*CASR, CLDN10, SLC12A3*
Hypocalciuria	9	*1.63*	0.33	3.64x10-7	*CASR, CLDN10, SLC12A3*
Abnormal blood cation concentration	150	*0.39*	0.08	3.46x10-7	*ALPL, SLC30A10, GATA3, CLDN10, SLC12A3, CYP24A1, CASR, NSD1, SLC34A1*

Gene set enrichment analyses using MAGMA to test 15,685 genes with kidney stone disease meta-analysis summary statistics. Overlapping genes have a significant p-value after Bonferroni adjustment for the number of genes per geneset. GO= gene ontology; HPO= human phenotype ontology; SE= standard error

Supplementary Table 11: Significantly enriched gene expression in GTEx v8 kidney cortex tissue from UK Biobank-FinnGen meta-analysis

Gene	Symbol[a]	Expression log transformed	Expression normalised (zero mean)
ENSG00000036828	*CASR*	2.318651	2.214128
ENSG00000100218	*RTDR1*	1.664179	0.9625
ENSG00000121068	*TBX2*	5.548159	1.497086
ENSG00000134873	*CLDN10*	4.771091	3.313177
ENSG00000156222	*SLC28A1*	4.422584	4.120758
ENSG00000159261	*CLDN14*	1.636429	1.413068
ENSG00000160951	*PTGER1*	3.280588	2.436819
ENSG00000164626	*KCNK5*	4.749444	2.348992
ENSG00000165125	*TRPV6*	3.120967	1.752797
ENSG00000174607	*UGT8*	3.187313	1.721579
ENSG00000223392	*CLDN10-AS1*	0.962256	0.812909
ENSG00000267280	*TBX2-AS1*	3.642846	1.267815

[a]Genes with unique Entrez IDs and gene symbols.
12 genes in GTEx v8 kidney cortex tissue showed significant (absolute log-fold change ≥0.58 and Bonferroni corrected p-value $<1.3x10^{-5}$) differential gene expression with kidney stone disease GWAS meta-analysis

Supplementary Table 12: Heritability estimates and genetic correlation of kidney stone disease GWAS with GWAS of measures of adiposity.

Trait	N	λGC	Mean Chi2	LDSC intercept (SE)	Ratio (SE)	h2 estimate on liability scale (SE)	Genetic correlation with UK Biobank KSD (SE)	P-value for genetic correlation with UK Biobank KSD	Genetic correlation with FinnGen KSD (SE)	P-value for genetic correlation with FinnGen KSD	Genetic correlation with MA KSD (SE)	P-value for genetic correlation with MA KSD
UK Biobank KSD	397,323 (8,504 cases; 388,819 controls)	1.10	1.27	1.01 (0.01)	0.07 (0.06)	0.22 (0.02)	-	-	-0.81 (0.07)	3.24x10-33	-	-
FinnGen KSD	341725 (8,597 cases; 333,128 controls)	1.15	1.18	1.04 (0.01)	0.21 (0.06)	0.23 (0.02)	- 0.81 (0.07)	3.24x10-33	-	-	-	-
UK Biobank-FinnGen meta-analysis KSD	739,048 (17,101 cases; 721,947 controls)	1.12	1.27	1.0276 (0.0086)	0.10 (0.03)	0.22 (0.02)	-	-	-	-	-	-
BMI	806834	2.70	3.88	1.02 (0.02)	0.01 (0.01)	0.18 (0.01)	-0.17 (0.03)	1.94x10-10	0.10 (0.03)	4.00x10-4	0.15 (0.02)	9.72x10-10
WHR	697734	2.11	2.75	1.10 (0.03)	0.06 (0.02)	0.12 (4.00x10-3)	-0.24 (0.03)	4.86x10-16	0.14 (0.03)	6.17x10-7	0.20 (0.02)	2.45x10-17
WC	224459	0.25	0.35	0.21 (3.00x10-3)	NA (Mean Chi2 >1)	0.03 (0.18x10-3)	-0.19 (0.04)	3.17x10-7	0.09 (0.04)	0.02	0.15 (0.03)	2.96x10-06

Supplementary Table 13: Univariable Mendelian randomization analyses in UK Biobank

Analysis				Inverse variance weighted				Intercept		MR Egger			
				Estimate		Heterogeneity				Estimate		Heterogeneity	
Group	Exposure	Outcome	N SNP	OR (95% CI)	p*	Q	p	Beta (SE)	p	OR (95% CI)	p	Q	p
Adiposity	ASAT	KSD	5	**1.5 (0.9-2.52)**	**0.23**	12.97	0.01	0.05 (0.05)	0.35	0.59 (0.1-3.32)	0.59	9.19	0.03
Adiposity	ASAT/GFAT	KSD	14	**1.15 (0.85-1.55)**	**0.59**	40.98	9.59x10-5	8.99x10-4 (0.03)	0.98	1.13 (0.31-4.04)	0.86	40.98	4.93x10-5
Adiposity	BMI	KSD	593	**1.4 (1.27-1.55)**	**8.96x10-10**	753.62	6.94x10-6	9.83x10-5 (2.25x10-3)	0.97	1.4 (1.06-1.84)	0.02	753.62	6.11x10-6
Adiposity	BMI- IVs in WHR genetic instrument removed	KSD	567	**1.44 (1.3-1.6)**	**2.07x10-10**	717.20	1.55x10-5	-4.79x10-4 (2.42x10-3)	0.84	1.44 (1.3-1.6)	9.42x10-12	717.20	1.55x10-5
Adiposity	BMI	KSD (Males)	185	**1.57 (1.28-1.92)**	**1.31x10-4**	646.25	1.08x10-52	-5.18x10-4 (6.40x10-3)	0.94	1.59 (1.06-2.38)	0.03	646.22	5.78x10-53
Adiposity	BMI	KSD (Females)	257	1.36 (1.12-1.65)	9.83x10-3	292.46	0.06	0.02 (5.64x10-3)	5.84x10-3	**0.65 (0.38-1.13)**	**0.13**	283.85	0.1
Adiposity	GFAT	KSD	15	**1.15 (0.85-1.54)**	**0.58**	36.82	7.86x10-4	-0.04 (0.03)	0.21	2.51 (0.76-8.29)	0.16	32.44	2.06x10-3
Adiposity	VAT	KSD	4	**1.45 (0.93-2.27)**	**0.21**	5.19	0.16	0.06 (0.17)	0.77	0.45 (4.17x10-4-478.69)	0.84	4.91	0.09
Adiposity	VAT/ASAT	KSD	19	**1.09 (0.95-1.25)**	**0.36**	15.62	0.62	-9.97x10-4 (0.01)	0.94	1.11 (0.73-1.67)	0.63	15.61	0.55
Adiposity	VAT/GFAT	KSD	19	**1.19 (0.97-1.45)**	**0.21**	34.21	0.01	-4.18x10-3 (0.02)	0.82	1.27 (0.7-2.3)	0.45	34.11	8.13x10-3
Adiposity	WC	KSD	44	**1.39 (1.09-1.77)**	**0.03**	68.47	8.05x10-3	0.01 (0.01)	0.22	0.84 (0.36-1.93)	0.68	66.04	0.01
Adiposity	WC	KSD (Males)	15	**1.92 (1.28-2.89)**	**1.88x10-5**	32.96	2.92x10-3	-8.01x10-3 (0.03)	0.79	2.31 (0.57-9.34)	0.26	32.78	1.84x10-3
Adiposity	WC	KSD (Females)	20	**1.16 (0.71-1.91)**	**0.73**	30.57	0.04	0.05 (0.03)	0.14	0.30 (0.05-1.81)	0.21	27.04	0.08
Adiposity	WHR	KSD	287	**1.42 (1.24-1.63)**	**1.28x10-5**	375.95	2.77x10-4	-5.45x10-4 (3.36x10-3)	0.87	1.47 (0.99-2.18)	0.06	375.92	2.39x10-4
Adiposity	WHR- IVs in BMI genetic instrument removed	KSD	261	**1.43 (1.24-1.66)**	**1.31x10-5**	344.00	3.67x10-4	8.86x10-4 (3.50x10-3)	0.80	1.36 (0.9-2.07)	0.14	343.92	3.18x10-4
Adiposity	WHR	KSD (Males)	73	**1.76 (1.34-2.3)**	**2.99x10-4**	107.2	4.51x10-3	-7.17x10-3 (0.01)	0.54	2.39 (0.87-6.59)	0.1	106.62	3.99x10-3
Adiposity	WHR	KSD (Females)	184	**1.31 (1.06-1.62)**	**0.03**	250.14	7.17x10-4	7.75x10-3 (6.27x10-3)	0.22	0.98 (0.59-1.63)	0.93	248.06	8.20x10-4
Biochemistry	Heel bone mineral density	KSD	1016	**0.95 (0.89-1)**	**0.17**	1524.3	3.77x10-23	9.05x10-5 (1.43x10-3)	0.95	0.94 (0.84-1.06)	0.32	1524.29	3.07x10-23
Biochemistry	Serum 25-OH vitamin D concentration	KSD	172	**1.25 (1.05-1.48)**	**0.03**	418.16	1.01x10-22	-3.02x10-3 (3.72x10-3)	0.42	1.36 (1.04-1.78)	0.03	416.55	1.03x10-22
Biochemistry	Serum calcium concentration†	KSD	185	**1.59 (1.32-1.91)**	**1.51x10-5**	814.17	4.62x10-80	-3.09x10-3 (5.81x10-3)	0.60	1.73 (1.2-2.49)	3.85x10-3	812.92	3.56x10-80

Biochemistry	Serum phosphate concentration	KSD	95	**0.76 (0.6-0.96)**	**0.07**	321.38	1.24x10-26	-7.99x10-3 (6.67x10-3)	0.23	0.94 (0.62-1.43)	0.76	316.5	3.81x10-26
Biochemistry	Urate	KSD	353	**0.96 (0.85-1.07)**	**0.69**	955.97	2.87x10-57	-4.32x10-3 (2.55x10-3)	0.09	1.07 (0.9-1.27)	0.46	948.2	2.04x10-56
Metabolic Syndrome	2 hour glucose tolerance	KSD	12	**0.92 (0.68-1.24)**	**0.73**	49.76	6.92x10-7	0.04 (0.03)	0.25	0.58 (0.27-1.27)	0.2	43.22	4.55x10-6
Metabolic Syndrome	DBP	KSD	793	**1 (0.99-1.01)**	**0.73**	1275.56	2.95x10-25	-1.66x10-3 (2.10x10-3)	0.43	1.01 (0.98-1.03)	0.62	1274.55	2.82x10-25
Metabolic Syndrome	Fasting glucose adjusted for BMI	KSD	87	**1.05 (0.83-1.32)**	**0.85**	148.42	3.39x10-5	1.76x10-3 (4.98x10-3)	0.72	0.98 (0.64-1.51)	0.94	148.2	2.65x10-5
Metabolic Syndrome	Fasting insulin adjusted for BMI	KSD	42	**1.43 (0.83-2.47)**	**0.36**	104.47	1.89x10-7	-2.29x10-3 (0.01)	0.87	1.63 (0.31-8.68)	0.57	104.4	1.17x10-7
Metabolic Syndrome	HbA1c	KSD	72	**1.32 (0.95-1.84)**	**0.21**	120.47	2.26x10-4	2.32x10-3 (5.24x10-3)	0.66	1.18 (0.64-2.17)	0.61	120.13	1.82x10-4
Metabolic Syndrome	SBP	KSD	756	**1 (0.99-1.01)**	**0.87**	1282.14	6.58x10-30	2.44x10-3 (2.24x10-3)	0.28	0.99 (0.98-1.01)	0.37	1280.13	7.65x10-30
Metabolic Syndrome	Serum HDL concentration	KSD	617	0.99 (0.92-1.06)	0.87	936.06	1.27x10-15	-4.17x10-3 (1.53x10-3)	6.55x10-3	1.11 (1-1.24)	**0.06**	924.86	7.09x10-15
Metabolic Syndrome	Serum LDL concentration	KSD	278	**0.99 (0.91-1.08)**	**0.87**	400.28	1.72x10-6	3.78x10-3 (2.25x10-3)	0.09	0.91 (0.79-1.04)	0.15	396.24	2.71x10-6
Metabolic Syndrome	Serum TG concentration	KSD	528	1.12 (1.03-1.22)	0.03	856	4.54x10-18	4.50x10-3 (1.73x10-3)	9.29x10-3	**0.98 (0.86-1.11)**	**0.75**	845.05	2.96x10-17
Metabolic Syndrome	T2D	KSD	367	1.12 (1.06-1.17)	5.01x10-5	556.49	4.76x10-10	5.82x10-3 (2.45x10-3)	0.02	**1 (0.91-1.11)**	**0.96**	548.03	1.68x10-9
Systemic inflammation	APO-B	KSD	318	**0.98 (0.91-1.05)**	**0.73**	422.81	6.29x10-5	1.49x10-3 (1.96x10-3)	0.45	0.95 (0.85-1.06)	0.34	422.04	5.97x10-5
Systemic inflammation	CRP	KSD	72	**1.1 (0.98-1.23)**	**0.21**	156.51	2.21x10-8	-1.58x10-3 (4.83x10-3)	0.74	1.12 (0.94-1.33)	0.2	156.27	1.56x10-8
Exposure group	Exposure	Outcome	N SNP	Beta (SE)	p*	Q	p	Beta (SE)	p	Beta (SE)	p	Q	p
Adiposity	KSD	BMI	15	**-2.46x10-4 (8.87x10-3)**	**0.98**	54.67	9.87x10-7	-1.89x10-3 (3.14x10-3)	0.56	0.01 (0.02)	0.59	53.18	8.39x10-7
Adiposity	KSD	WC	10	**-8.58x10-3 (0.01)**	**0.73**	10.67	0.3	-3.99x10-3 (4.44x10-3)	0.4	0.02 (0.04)	0.57	9.69	0.29
Adiposity	KSD	WHR	15	**0.01 (5.70x10-3)**	**0.15**	21.27	0.09	6.25x10-4 (2.07x10-3)	0.77	6.31x10-3 (0.02)	0.7	21.12	0.07
Adiposity	BMI	Heel bone mineral density	593	**0.12 (0.02)**	**2.43x10-12**	4798.27	0.00	-7.94x10-4 (6.75x10-4)	0.24	0.16 (0.04)	1.29x10-4	4787.05	0.00
Adiposity	BMI	Serum 25-OH vitamin D concentration	584	**0.04 (9.64x10-3)**	**1.86x10-4**	1501.32	3.23x10-82	-4.80x10-4 (4.23x10-4)	0.26	0.07 (0.03)	0.01	1498	5.55x10-82
Adiposity	BMI	Serum calcium concentration†	592	**0.04 (0.01)**	**7.42x10-3**	2115.09	4.29x10-170	3.49x10-4 (6.00x10-4)	0.56	0.02 (0.04)	0.54	2113.87	3.51x10-170
Adiposity	BMI	Serum calcium concentration†	592	**0.04 (0.01)**	**7.42x10-3**	2115.09	4.29x10-170	3.49x10-4 (6.00x10-4)	0.56	0.02 (0.04)	0.54	2113.87	3.51x10-170
Adiposity	BMI- IVs in WHR genetic instrument removed	Serum calcium concentration†	566	0.04 (0.01)	0.02	1959.40	5.79x10-153	3.95x10-153 (2.82x10-4)	6.37x10-4	**0.02 (0.04)**	**0.58**	1958.72	3.95x10-153
Adiposity	BMI	Serum phosphate concentration	590	**-0.03 (0.01)**	**0.06**	1686.76	1.94x10-106	1.21x10-4 (5.33x10-4)	0.82	-0.04 (0.03)	0.28	1686.61	1.20x10-106
Adiposity	WC	Serum calcium concentration†	40	**0.07 (0.03)**	**0.08**	137.80	5.94x10-13	-6.63x10-4 (3.00x10-3)	0.82	0.09 (0.11)	0.42	137.62	3.28x10-13

Adiposity	WHR	Heel bone mineral density	286	0.14 (0.03)	1.95x10-6	3462.66	0.00	-3.14x10-3 (1.20x10-3)	9.56x10-3	**0.31 (0.07)**	**1.99x10-5**	3381.6	0.00
Adiposity	WHR	Serum 25-OH vitamin D concentration	275	**-0.04 (0.02)**	**0.04**	1035.11	7.86x10-89	-3.25x10-4 (8.84x10-4)	0.71	-0.02 (0.05)	0.68	1034.60	4.87x10-89
Adiposity	WHR	Serum calcium concentration†	286	**0.1 (0.02)**	**1.53x10-6**	1085.36	1.08x10-93	1.47x10-3 (9.08x10-4)	0.11	0.02 (0.05)	0.7	1075.38	2.20x10-92
Adiposity	WHR	Serum calcium concentration†	286	**0.1 (0.02)**	**1.53x10-6**	1085.36	1.08x10-93	1.47x10-3 (9.08x10-4)	0.11	0.02 (0.05)	0.7	1075.38	2.20x10-92
Adiposity	WHR- IVs in BMI genetic instrument removed	Serum calcium concentration†	260	0.10 (0.02)	1.66x10-6	931.69	1.13x10-76	2.98x10-77 (1.24x10-3)	9.19x10-4	**0.03 (0.06)**	**0.53**	925.18	6.30x10-76
Adiposity	WHR	Serum phosphate concentration	285	**-6.29x10-3 (0.02)**	**0.86**	984.42	4.97x10-78	1.95x10-4 (8.62x10-4)	0.82	-0.02 (0.05)	0.74	984.24	2.84x10-78
Biochemistry	Heel bone mineral density	Serum calcium concentration†	499	**-0.02 (0.01)**	**0.04**	1988.64	9.30x10-177	-1.94x10-4 (4.64x10-4)	0.68	-0.02 (0.02)	0.28	1987.93	6.04x10-177
Biochemistry	KSD	Serum calcium concentration†	15	**-2.46x10-4 (8.87x10-3)**	**0.98**	54.67	9.87x10-7	-1.89x10-3 (3.14x10-3)	0.56	0.01 (0.02)	0.59	53.18	8.39x10-7
Biochemistry	Serum calcium concentration†	BMI	172	**-9.8x10-3 (0.02)**	**0.73**	1552.3	4.88x10-221	1.01x10-3 (1.02x10-3)	0.32	-0.04 (0.03)	0.25	1543.45	8.34x10-220
Biochemistry	Serum calcium concentration†	Serum 25-OH vitamin D concentration	172	**-0.02 (0.02)**	**0.47**	1124.32	6.40x10-140	2.24x10-3 (9.45x10-4)	0.02	-0.08 (0.03)	0.01	1088.23	1.11x10-133
Biochemistry	Serum calcium concentration†	WHR	172	**0.03 (0.01)**	**0.03**	840.87	5.27x10-89	8.79x10-4 (7.78x10-4)	0.26	6.89x10-3 (0.02)	0.78	834.6	2.90x10-88
Biochemistry	Serum phosphate concentration	BMI	118	**-0.01 (0.02)**	**0.73**	861.85	7.99x10-114	-9.40x10-4 (1.06x10-3)	0.38	0.01 (0.04)	0.69	856.02	3.67x10-113

†Albumin adjusted serum calcium concentration; APO-B= apolipoprotein-B; ASAT=abdominal subcutaneous adipose tissue; BMI = body mass index; CI=confidence interval; CRP= C-reactive protein; GFAT= gluteofemoral adipose tissue; HDL = high density lipoprotein; LDL = low density lipoprotein; KSD = kidney stone disease; LDL = low density lipoprotein; N SNP= number of single nucleotide polymorphisms in analysis; OR=odds ratio for outcome per 1 standard deviation increase in genetically-instrumented exposure variable; p* = p value adjusted for multiple testing using 5% false discovery rate method; SE= standard error; T2D= type 2 diabetes; TG = triglyceride; VAT= visceral adipose tissue; WHR = waist-to-hip ratio; 25-OH vitamin D = hydroxyvitamin D. Bold text highlights the sensitivity analysis to be interpreted after considering the estimate of the intercept.

Supplementary Table 14: Univariable Mendelian randomization analyses on risk of kidney stone disease in FinnGen study

Analysis				Inverse variance weighted				Intercept		MR Egger			
				Estimate		Heterogeneity				Estimate		Heterogeneity	
Group	Exposure	Outcome	N SNP	OR (95% CI)	p*	Q	p	Beta (SE)	p	OR (95% CI)	p	Q	p
Adiposity	ASAT	KSD	5	**1.59 (0.68-3.72)**	**0.48**	31.99	1.92x10-6	-0.07 (0.09)	0.47	5.87 (0.23-146.93)	0.36	26.07	9.20x10-6
Adiposity	ASAT/GFAT	KSD	13	**1.02 (0.67-1.55)**	**0.95**	61.3	1.31x10-8	-0.06 (0.04)	0.23	2.88 (0.55-15.18)	0.24	53.54	1.43x10-7
Adiposity	BMI	KSD	574	**1.29 (1.16-1.44)**	**3.63x10-5**	783.21	1.07x10-8	-3.95x10-3 (2.38x10-3)	0.10	1.62 (1.21-2.17)	1.23x10-3	779.48	1.53x10-8
Adiposity	BMI-instruments in WHR instrumental variable removed	KSD	551	**1.32 (1.18-1.48)**	**9.86x10-6**	753.47	1.62x10-8	-5.70x10-3 (2.54x10-3)	0.03	1.86 (1.35-2.55)	1.47x10-4	746.62	3.55x10-8
Adiposity	GFAT	KSD	14	**0.96 (0.67-1.37)**	**0.87**	49.01	4.41x10-6	0.03 (0.03)	0.41	0.58 (0.17-1.93)	0.39	46.18	6.46x10-6
Adiposity	VAT	KSD	4	**1.17 (0.38-3.59)**	**0.87**	31.88	5.54x10-7	-0.03 (0.44)	0.96	2.05 (2.87x10-8-146134168.23)	0.94	31.83	1.23x10-7
Adiposity	VAT/ASAT	KSD	16	**1.17 (0.98-1.4)**	**0.18**	22.4	0.1	0.02 (0.02)	0.28	0.89 (0.53-1.48)	0.66	20.54	0.11
Adiposity	VAT/GFAT	KSD	16	**1.24 (0.94-1.63)**	**0.25**	46.7	4.11x10-5	4.87x10-3 (0.03)	0.85	1.15 (0.5-2.61)	0.75	46.58	2.25x10-5
Adiposity	WC	KSD	43	**1.3 (0.98-1.73)**	**0.17**	92.32	1.22x10-5	3.87x10-3 (0.01)	0.78	1.13 (0.41-3.13)	0.81	92.15	8.38x10-6
Adiposity	WHR	KSD	271	**1.23 (1.05-1.45)**	**0.03**	434.33	8.43x10-10	-1.68x10-3 (3.75x10-3)	0.66	1.36 (0.87-2.11)	0.18	434.01	7.02x10-10
Adiposity	WHR-instruments in BMI instrumental variable removed	KSD	248	**1.23 (1.04-1.46)**	**0.05**	408.91	4.03x10-10	-6.81x10-4 (3.94x10-3)	0.86	1.28 (0.8-2.03)	0.3	408.86	3.14x10-10
Biochemistry	Heel bone mineral density	KSD	971	**0.97 (0.91-1.03)**	**0.58**	1450.18	9.85x10-22	-1.02x10-3 (1.46x10-3)	0.49	1.01 (0.9-1.13)	0.91	1449.45	9.08x10-22
Biochemistry	Serum 25-OH vitamin D concentration	KSD	158	**1.21 (1.03-1.43)**	**0.06**	386.47	2.31x10-21	-2.16x10-3 (3.81x10-3)	0.57	1.28 (0.99-1.66)	0.06	385.68	1.85x10-21
Biochemistry	Serum calcium concentration†	KSD	164	**1.42 (1.19-1.7)**	**7.98x10-4**	652.89	7.98x10-60	-8.21x10-3 (5.68x10-3)	0.15	1.76 (1.25-2.47)	1.39x10-3	644.57	9.14x10-59
Biochemistry	Serum phosphate concentration	KSD	85	**0.76 (0.62-0.94)**	**0.04**	230.35	1.44x10-15	-8.92x10-3 (6.01x10-3)	0.14	0.94 (0.66-1.32)	0.71	224.4	5.91x10-15
Biochemistry	Urate	KSD	315	**0.96 (0.85-1.09)**	**0.73**	790.14	7.04x10-43	-4.13x10-3 (2.65x10-3)	0.12	1.07 (0.9-1.29)	0.44	784.04	2.82x10-42
Metabolic Syndrome	2 hour glucose tolerance	KSD	9	**0.96 (0.68-1.36)**	**0.87**	38.47	6.16x10-6	0.05 (0.04)	0.23	0.55 (0.23-1.35)	0.23	30.97	6.30x10-5
Metabolic Syndrome	DBP	KSD	747	**1.00 (0.99-1.01)**	**0.81**	1141.67	3.95x10-19	-1.72x10-4 (2.11x10-3)	0.94	1 (0.98-1.03)	0.8	1141.66	3.19x10-19
Metabolic Syndrome	Fasting glucose adjusted for BMI	KSD	82	**1.01 (0.84-1.23)**	**0.92**	94.37	0.15	7.70x10-3 (4.12x10-3)	0.07	0.77 (0.55-1.09)	0.14	90.42	0.2

Metabolic Syndrome	Fasting insulin adjusted for BMI	KSD	41	**1.6 (0.96-2.67)**	**0.17**	82.43	9.02x10-5	9.83x10-3 (0.01)	0.46	0.91 (0.19-4.34)	0.91	81.27	8.40x10-5
Metabolic Syndrome	HbA1c	KSD	70	**1.08 (0.79-1.48)**	**0.8**	89.81	0.05	-4.51x10-3 (5.10x10-3)	0.38	1.38 (0.74-2.57)	0.32	88.79	0.05
Metabolic Syndrome	SBP	KSD	722	**1.00 (1-1.01)**	**0.73**	1086.28	2.90x10-17	1.85x10-3 (2.17x10-3)	0.39	1 (0.98-1.01)	0.59	1085.18	2.84x10-17
Metabolic Syndrome	Serum HDL concentration	KSD	569	**1.09 (1.01-1.17)**	**0.06**	848.7	1.78x10-13	3.36x10-4 (1.59x10-3)	0.83	1.08 (0.96-1.21)	0.19	848.63	1.46x10-13
Metabolic Syndrome	Serum LDL concentration	KSD	250	**1.01 (0.91-1.11)**	**0.94**	412.4	3.33x10-10	1.89x10-3 (2.47x10-3)	0.45	0.97 (0.84-1.11)	0.63	411.43	3.12x10-10
Metabolic Syndrome	Serum TG concentration	KSD	496	**1.08 (1-1.17)**	**0.14**	748.83	1.15x10-12	2.92x10-3 (1.67x10-3)	0.08	1 (0.89-1.13)	0.99	744.2	2.07x10-12
Metabolic Syndrome	T2D	KSD	351	**1.08 (1.02-1.13)**	**0.03**	606.01	5.46x10-16	2.35x10-3 (2.76x10-3)	0.39	1.03 (0.92-1.15)	0.61	604.75	5.42x10-16
Systemic inflammation	APO-B	KSD	286	**1.03 (0.95-1.12)**	**0.69**	422.18	2.26x10-7	1.37x10-3 (2.19x10-3)	0.53	1 (0.89-1.13)	0.95	421.6	2.03x10-7
Systemic inflammation	CRP	KSD	69	1.01 (0.9-1.13)	0.92	153.87	1.34x10-8	0.01 (4.75x10-3)	0.02	**0.87 (0.74-1.03)**	**0.11**	142.52	2.23x10-7

†Albumin adjusted serum calcium concentration; APO-B= apolipoprotein-B; ASAT=abdominal subcutaneous adipose tissue; BMI = body mass index; CI=confidence interval; CRP= C-reactive protein; GFAT= gluteofemoral adipose tissue; HDL = high density lipoprotein; LDL = low density lipoprotein; KSD = kidney stone disease; LDL = low density lipoprotein; N SNP= number of single nucleotide polymorphisms in analysis; OR=odds ratio for outcome per 1 standard deviation increase in genetically-instrumented exposure variable; p* = p value adjusted for multiple testing using 5% false discovery rate method; SE= standard error; T2D= type 2 diabetes; TG = triglyceride; VAT= visceral adipose tissue; WHR = waist-to-hip ratio; 25-OH vitamin D = hydroxyvitamin D. Bold text highlights the sensitivity analysis to be interpreted after considering the estimate of the intercept.

Supplementary Table 15: Steiger test for directionality of instrumental variables used in Mendelian randomisation

Exposure Group	Exposure	Outcome	Exposure SNP r2	Outcome SNP r2	Steiger p
Adiposity	KSD (UK Biobank)	BMI	0.01	6.61x10-5	0.00
Adiposity	KSD (UK Biobank)	WC	7.62x10-3	7.49x10-5	8.38x10-156
Adiposity	KSD (UK Biobank)	WHR	0.01	3.82x10-5	0.00
Adiposity	KSD (Meta-analysis)	BMI	0.03	9.33x10-4	0.00
Adiposity	KSD (Meta-analysis)	WC	0.02	9.03x10-4	1.14x10-273
Adiposity	KSD (Meta-analysis)	WHR	0.03	4.35x10-4	0.00
Adiposity	ASAT	KSD (UK Biobank)	5.71x10-3	3.78x10-4	2.84x10-26
Adiposity	ASAT	KSD (FinnGen)	5.71x10-3	7.57x10-4	1.92x10-19
Adiposity	ASAT	KSD (Meta-analysis)	4.86x10-3	3.98x10-4	8.50x10-22
Adiposity	ASAT/GSAT	KSD (UK Biobank)	0.02	7.98x10-4	1.41x10-89
Adiposity	ASAT/GSAT	KSD (FinnGen)	0.02	1.23x10-3	8.44x10-72
Adiposity	ASAT/GSAT	KSD (Meta-analysis)	0.02	8.54x10-4	1.00x10-85
Adiposity	BMI	24h urine calcium	0.05	0.32	3.05x10-74
Adiposity	BMI	KSD (UK Biobank)	5.12x10-2	0.02	0.00
Adiposity	BMI	KSD (FinnGen)	0.05	0.02	0.00
Adiposity	BMI	KSD (Meta-analysis)	4.91x10-2	0.01	0.00
Adiposity	BMI- SNPs in WHR IV removed	KSD (UK Biobank)	0.05	0.01	0.00
Adiposity	BMI- SNPs in WHR IV removed	KSD (FinnGen)	0.05	0.02	0.00
Adiposity	BMI- SNPs in WHR IV removed	KSD (Meta-analysis)	0.04	0.01	0.00
Adiposity	BMI	KSD (UK Biobank males)	0.06	0.02	2.27x10-255
Adiposity	BMI	KSD (UK Biobank females)	4.34x10-2	0.02	0.00
Adiposity	BMI	Heel bone mineral density	5.11x10-2	0.01	0.00
Adiposity	BMI	Serum 25-OH vitamin D	5.05x10-2	3.27x10-3	0.00
Adiposity	BMI	Serum calcium concentration†	5.12x10-2	7.06x10-3	0.00
Adiposity	BMI- SNPs in WHR IV removed	Serum calcium concentration†	0.03	3.37x10-3	0.00
Adiposity	BMI	Serum phosphate concentration	5.08x10-2	5.64x10-3	0.00
Adiposity	BMI	WHR	5.13x10-2	0.02	0.00
Adiposity	GFAT	KSD (UK Biobank)	1.60x10-2	8.76x10-4	3.36x10-75
Adiposity	GFAT	KSD (FinnGen)	1.61x10-2	9.56x10-4	4.84x10-73
Adiposity	GFAT	KSD (Meta-analysis)	1.69x10-2	9.27x10-4	5.97x10-83
Adiposity	VAT	KSD (UK Biobank)	4.09x10-3	1.81x10-4	1.54x10-21
Adiposity	VAT	KSD (FinnGen)	4.09x10-3	6.00x10-4	1.40x10-13
Adiposity	VAT	KSD (Meta-analysis)	3.14x10-3	2.54x10-4	1.14x10-14
Adiposity	VAT/ASAT	KSD (UK Biobank)	0.03	3.36x10-4	1.98x10-210
Adiposity	VAT/ASAT	KSD (FinnGen)	0.03	6.16x10-4	1.88x10-177
Adiposity	VAT/ASAT	KSD (Meta-analysis)	0.03	3.49x10-4	5.84x10-211
Adiposity	VAT/GSAT	KSD (UK Biobank)	0.02	7.30x10-4	5.73x10-136
Adiposity	VAT/GSAT	KSD (FinnGen)	0.02	1.10x10-3	1.96x10-111
Adiposity	VAT/GSAT	KSD (Meta-analysis)	0.02	8.55x10-4	4.55x10-137
Adiposity	WC	KSD (UK Biobank males)	8.73x10-3	1.57x10-3	1.03x10-43
Adiposity	WC	KSD (UK Biobank females)	9.26x10-3	1.68x10-3	1.99x10-55
Adiposity	WC	KSD (UK Biobank)	0.01	1.59x10-3	1.47x10-147
Adiposity	WC	KSD (FinnGen)	0.01	2.18x10-3	5.62x10-106
Adiposity	WC	KSD (Meta-analysis)	0.01	1.53x10-3	3.40x10-176
Adiposity	WC	Serum calcium concentration†	0.01	6.04x10-4	1.21x10-196
Adiposity	WC	Serum phosphate concentration	0.01	1.25x10-3	1.05x10-151
Adiposity	WHR	24h urine calcium	0.03	0.16	3.18x10-31
Adiposity	WHR	KSD (UK Biobank males)	0.01	3.68x10-3	1.37x10-77
Adiposity	WHR	KSD (UK Biobank females)	0.04	0.01	5.18x10-181
Adiposity	WHR	KSD (UK Biobank)	0.03	7.60x10-3	0.00
Adiposity	WHR	KSD (FinnGen)	0.03	8.30x10-3	2.74x10-288
Adiposity	WHR	KSD (Meta-analysis)	0.03	5.15x10-3	0.00
Adiposity	WHR- SNPs in BMI IV removed	KSD (UK Biobank)	0.03	6.99x10-3	0.00
Adiposity	WHR- SNPs in BMI IV removed	KSD (FinnGen)	0.02	7.79x100-3	2.86x10-246
Adiposity	WHR- SNPs in BMI IV removed	KSD (Meta-analysis)	0.02	4.63x10-3	0.00
Adiposity	WHR	BMI	0.03	0.02	2.07x10-115
Adiposity	WHR	Heel bone mineral density	0.023	0.01	5.09x10-265
Adiposity	WHR	Serum 25-OH vitamin D	0.03	2.14x10-3	0.00
Adiposity	WHR	Serum calcium concentration†	0.03	3.91x10-3	0.00
Adiposity	WHR- SNPs in BMI IV removed	Serum calcium concentration†	0.05	6.52x10-3	0.00
Adiposity	WHR	Serum phosphate concentration	0.03	3.26x10-3	0.00
Biochemistry	KSD (UK Biobank)	Serum calcium concentration†	0.01	1.73x10-4	0.00
Biochemistry	KSD (Meta-analysis)	Serum calcium concentration†	0.03	0.01	4.10x10-265
Biochemistry	Heel bone mineral density	KSD (UK Biobank)	0.22	0.03	0.00
Biochemistry	Heel bone mineral density	KSD (FinnGen)	0.21	0.03	0.00
Biochemistry	Heel bone mineral density	KSD (Meta-analysis)	0.21	0.02	0.00
Biochemistry	Heel bone mineral density	Serum calcium concentration†	0.01	1.37x10-3	1.79x10-159
Biochemistry	Serum 25-OH vitamin D	KSD (UK Biobank)	0.03	8.01x10-3	0.00
Biochemistry	Serum 25-OH vitamin D	KSD (FinnGen)	0.03	7.63x10-3	0.00
Biochemistry	Serum 25-OH vitamin D	KSD (Meta-analysis)	0.03	5.58x10-3	0.00

Biochemistry	Serum 25-OH vitamin D	Serum calcium concentration†	0.03	5.52x10-3	0.00
Biochemistry	Serum calcium concentration†	KSD (UK Biobank)	0.06	0.02	0.00
Biochemistry	Serum calcium concentration†	KSD (FinnGen)	0.05	0.01	0.00
Biochemistry	Serum calcium concentration†	KSD (Meta-analysis)	0.06	0.01	0.00
Biochemistry	Serum calcium concentration†	Serum 25-OH vitamin D	0.05	2.30x10-3	0.00
Biochemistry	Serum calcium concentration†	WHR	0.05	1.26x10-3	0.00
Biochemistry	Serum phosphate concentration	KSD (UK Biobank)	0.04	9.94x10-3	0.00
Biochemistry	Serum phosphate concentration	KSD (FinnGen)	0.04	8.94x10-3	0.00
Biochemistry	Serum phosphate concentration	KSD (Meta-analysis)	0.04	7.86x10-3	0.00
Biochemistry	Urate	KSD (UK Biobank)	0.11	0.02	0.00
Biochemistry	Urate	KSD (FinnGen)	0.10	0.01	0.00
Biochemistry	Urate	KSD (Meta-analysis)	0.11	0.01	0.00
Metabolic syndrome	2 hour glucose tolerance adjusted for BMI	KSD (UK Biobank)	3.90x10-3	9.30x10-4	1.56x10-31
Metabolic syndrome	2 hour glucose tolerance adjusted for BMI	KSD (FinnGen)	3.51x10-3	7.38x10-4	2.92x10-30
Metabolic syndrome	2 hour glucose tolerance adjusted for BMI	KSD (Meta-analysis)	3.90x10-3	7.76x10-4	4.55x10-43
Metabolic syndrome	DBP	KSD (UK Biobank)	0.07	0.02	0.00
Metabolic syndrome	DBP	KSD (FinnGen)	0.07	0.02	0.00
Metabolic syndrome	DBP	KSD (Meta-analysis)	0.07	0.01	0.00
Metabolic syndrome	Fasting glucose adjusted for BMI	KSD (UK Biobank)	0.04	2.74x10-3	0.00
Metabolic syndrome	Fasting glucose adjusted for BMI	KSD (FinnGen)	0.04	1.74x10-3	0.00
Metabolic syndrome	Fasting glucose adjusted for BMI	KSD (Meta-analysis)	0.04	1.50x10-3	0.00
Metabolic syndrome	Fasting insulin adjusted for BMI	KSD (UK Biobank)	0.01	1.99x10-3	1.12x10-138
Metabolic syndrome	Fasting insulin adjusted for BMI	KSD (FinnGen)	0.01	1.64x10-3	3.49x10-139
Metabolic syndrome	Fasting insulin adjusted for BMI	KSD (Meta-analysis)	0.01	1.59x10-3	4.70x10-176
Metabolic syndrome	HbA1c	KSD (UK Biobank)	0.04	2.39x10-3	0.00
Metabolic syndrome	HbA1c	KSD (FinnGen)	0.04	1.77x10-3	0.00
Metabolic syndrome	HbA1c	KSD (Meta-analysis)	0.04	1.34x10-3	0.00
Metabolic syndrome	SBP	KSD (UK Biobank)	0.07	0.02	0.00
Metabolic syndrome	SBP	KSD (FinnGen)	0.07	0.02	0.00
Metabolic syndrome	SBP	KSD (Meta-analysis)	0.06	0.01	0.00
Metabolic syndrome	Serum HDL concentration	KSD (UK Biobank)	0.16	0.02	0.00
Metabolic syndrome	Serum HDL concentration	KSD (FinnGen)	0.15	0.02	0.00
Metabolic syndrome	Serum HDL concentration	KSD (Meta-analysis)	0.16	0.01	0.00
Metabolic syndrome	Serum LDL concentration	KSD (UK Biobank)	0.08	7.55x10-3	0.00
Metabolic syndrome	Serum LDL concentration	KSD (FinnGen)	0.07	7.90x10-3	0.00
Metabolic syndrome	Serum LDL concentration	KSD (Meta-analysis)	0.07	5.32x10-3	0.00
Metabolic syndrome	Serum TG concentration	KSD (UK Biobank)	0.11	0.02	0.00
Metabolic syndrome	Serum TG concentration	KSD (FinnGen)	0.11	0.01	0.00
Metabolic syndrome	Serum TG concentration	KSD (Meta-analysis)	0.11	0.01	0.00
Metabolic syndrome	T2D	KSD (UK Biobank)	0.02	0.01	8.71x10-87
Metabolic syndrome	T2D	KSD (FinnGen)	0.02	0.01	2.93x10-58
Metabolic syndrome	T2D	KSD (Meta-analysis)	0.02	7.98x10-3	2.03x10-262
Systemic inflammation	APOB	KSD (UK Biobank)	0.09	0.01	0.00
Systemic inflammation	APOB	KSD (FinnGen)	0.08	8.24x10-3	0.00
Systemic inflammation	APOB	KSD (Meta-analysis)	0.09	6.12x10-3	0.00
Systemic inflammation	CRP	KSD (UK Biobank)	0.04	2.98x10-3	0.00
Systemic inflammation	CRP	KSD (FinnGen)	0.04	2.87x10-3	0.00
Systemic inflammation	CRP	KSD (Meta-analysis)	0.04	2.19x10-3	0.00

†Albumin adjusted serum calcium concentration; APO-B= apolipoprotein-B; ASAT=abdominal subcutaneous adipose tissue; BMI = body mass index; CRP= C-reactive protein; GFAT= gluteofemoral adipose tissue; HDL = high density lipoprotein; IV= instrumental variable; LDL = low density lipoprotein; KSD = kidney stone disease; LDL = low density lipoprotein; p = p value; T2D= type 2 diabetes; TG = triglyceride; VAT= visceral adipose tissue; WHR = waist-to-hip ratio; 25-OH vitamin D = hydroxyvitamin D. Tests failing Steiger testing on initial inverse-weighted median and MR-Egger Mendelian randomisation estimates are highlighted in red.

Supplementary Table 16: Sensitivity analyses- Mendelian randomization analyses results after applying Steiger filtering to significant results in univariable Mendelian randomisation

Analysis				Inverse variance weighted				Intercept		MR Egger			
				Estimate		Heterogeneity				Estimate		Heterogeneity	
Exposure group	Exposure	Outcome	N SNP	OR (95% CI) or Beta (SE)	p*	Q	p	Beta	p	OR (95% CI) or Beta (SE)	p	Q	p
Adiposity	BMI	KSD (UK Biobank)	567	**1.36 (1.24-1.5)**	**6.00x10-10**	616.69	0.07	-5.26x10-5 (2.11x10-3)	0.98	1.37 (1.06-1.77)	0.02	616.69	0.07
Adiposity	BMI	KSD (FinnGen)	541	**1.27 (1.15-1.4)**	**8.62x10-6**	587.36	0.08	-2.34x10-3 (2.21x10-3)	0.29	1.45 (1.11-1.9)	6.81x10-3	586.14	0.08
Adiposity	BMI	KSD (Meta-analysis)	555	**1.36 (1.26-1.47)**	**4.95x10-13**	839.7	4.47x10-14	-9.90x10-4 (1.79x10-3)	0.58	1.44 (1.16-1.8)	1.01x10-3	839.23	3.92x10-14
Adiposity	BMI- instruments in WHR instrumental variable removed	KSD (UK Biobank)	543	**1.39 (1.26-1.53)**	**7.31x10-10**	594.62	0.06	-9.73x10-4 (2.29x10-3)	0.67	1.47 (1.11-1.95)	8.27x10-3	594.43	0.06
Adiposity	BMI- instruments in WHR instrumental variable removed	KSD (FinnGen)	519	**1.3 (1.17-1.43)**	**3.74x10-6**	561.83	0.09	-4.18x10-3 (2.35x10-3)	0.08	1.66 (1.24-2.23)	7.43x10-4	558.42	0.1
Adiposity	BMI- instruments in WHR instrumental variable removed	KSD (Meta-analysis)	531	**1.39 (1.28-1.52)**	**2.30x10-13**	801.51	2.07x10-13	-2.47x10-3 (1.92x10-3)	0.2	1.61 (1.27-2.05)	9.88x10-5	799.00	2.59x10-13
Adiposity	BMI	KSD (Males)	173	**1.45 (1.19-1.76)**	**1.89x10-4**	484.26	1.68x10-31	-2.36x10-3 (5.95x10-3)	0.69	1.55 (1.07-2.24)	0.02	483.81	1.15x10-31
Adiposity	BMI	24 hour urine calcium excretion	169	**0.77 (0.51-1.18)**	**0.24**	9.4	1.00	1.05x10-3 (0.02)	0.96	0.76 (0.28-2.07)	0.59	9.4	1.00
Adiposity	BMI	Heel bone mineral density	568	**1.12 (1.09-1.14)**	**3.31x10-24**	2232.3	1.05x10-195	-9.05x10-4 (4.77x10-4)	0.06	1.18 (1.11-1.25)	3.17x10-8	2218.19	1.03x10-193
Adiposity	BMI	Serum 25-OH vitamin D concentration	582	**1.04 (1.02-1.06)**	**2.74x10-5**	1415.57	2.18x10-71	-5.24x10-4 (4.11x10-4)	0.2	1.07 (1.02-1.13)	5.63x10-3	1411.63	4.47x10-71
Adiposity	BMI	Serum calcium concentration†	578	**1.05 (1.03-1.07)**	**2.69x10-5**	1207.47	8.85x10-47	1.14x10-4 (4.65x10-4)	0.81	1.04 (0.98-1.1)	0.17	1207.34	6.31x10-47
Adiposity	BMI	WHR	570	**1.62 (1.58-1.65)**	**0.00**	2381.89	1.29x10-219	-3.50x10-5 (4.63x10-4)	0.94	1.62 (1.53-1.71)	8.63x10-52	2381.87	6.35x10-220
Adiposity	WC	KSD (UK Biobank)	44	**1.39 (1.1-1.76)**	**0.01**	68.47	8.05x10-3	0.01 (0.01)	0.22	0.84 (0.36-1.93)	0.68	66.04	0.01
Adiposity	WC	KSD (Meta-analysis)	43	**1.35 (1.07-1.71)**	**0.02**	120.72	1.53x10-9	9.45x10-3 (0.01)	0.4	0.97 (0.43-2.18)	0.94	118.67	1.76x1x10-9
Adiposity	WC	KSD (Males)	15	**1.92 (1.27-2.89)**	**2.76x10-3**	32.96	2.92x10-3	-8.01x10-3 (0.03)	0.79	2.31 (0.57-9.34)	0.26	32.78	1.84x1x10-3
Adiposity	WHR	KSD (Males)	72	**1.7 (1.29-2.24)**	**1.85x10-4**	101.53	0.01	-9.40x10-3 (0.01)	0.41	2.56 (0.95-6.9)	0.07	100.55	9.78x10-3
Adiposity	WHR	KSD (Females)	167	**1.25 (1.02-1.52)**	**0.03**	180.16	0.21	3.85x10-3 (5.80x10-3)	0.51	1.09 (0.69-1.72)	0.72	179.68	0.21
Adiposity	WHR	KSD (UK Biobank)	277	**1.35 (1.18-1.55)**	**2.74x10-5**	327.15	0.02	-2.03x10-3 (3.22x10-3)	0.53	1.51 (1.04-2.21)	0.03	326.68	0.02
Adiposity	WHR	KSD (FinnGen)	257	**1.21 (1.03-1.41)**	**0.02**	357.82	2.66x10-5	-2.14x10-3 (3.55x10-3)	0.55	1.36 (0.9-2.06)	0.15	357.31	2.41x1x10-5
Adiposity	WHR	KSD (Meta-analysis)	261	**1.31 (1.16-1.47)**	**2.07x10-5**	447.29	4.33x10-12	-1.33x10-3 (2.78x10-3)	0.63	1.41 (1.02-1.96)	0.04	446.89	3.57x10-12
Adiposity	WHR- instruments in BMI instrumental variable removed	KSD (UK Biobank)	252	**1.34 (1.17-1.55)**	**9.07x10-5**	302.04	0.02	-1.16x10-3 (3.37x10-3)	0.73	1.43 (0.96-2.13)	0.08	301.90	0.01
Adiposity	WHR- instruments in BMI instrumental variable removed	KSD (Meta-analysis)	236	**1.31 (1.15-1.48)**	**5.75x10-5**	407.70	2.00x10-11	-4.06x10-4 (2.90x10-3)	0.89	1.34 (0.95-1.88)	0.09	407.66	1.52x10-11

Adiposity	WHR	24 hour urine calcium excretion	75	**3.25 (0.63-16.7)**	**0.17**	4.1	1.00	-0.02 (0.05)	0.63	9.02 (0.1-787.88)	0.34	3.86	1.00
Adiposity	WHR	Heel bone mineral density	264	1.10 (1.06-1.15)	6.42x10-6	1791.84	2.39x10-225	-3.18x10-3 (9.15x10-4)	6.09x10-4	**1.32 (1.18-1.47)**	**1.01x10-6**	1713.12	3.31x10-211
Adiposity	WHR	Serum calcium concentration†	277	1.07 (1.03-1.12)	1.08x10-5	690.93	1.76x10-37	2.01x10-3 (7.56x10-4)	8.46x10-3	**0.96 (0.88-1.05)**	**0.40**	673.7	1.98x10-35
Biochemistry	Serum 25-OH vitamin D concentration	KSD (Meta-analysis)	158	**1.17 (1.04-1.32)**	**0.02**	413.66	5.37x10-25	-2.45x10-3 (2.82x10-3)	0.39	1.25 (1.03-1.52)	0.03	411.67	6.12x10-25
Biochemistry	Serum calcium concentration†	KSD (UK Biobank)	174	**1.51 (1.29-1.76)**	**5.45x10-6**	567.46	1.74x10-43	-2.02x10-3 (5.19x10-3)	0.7	1.59 (1.15-2.2)	5.19x10-3	566.96	1.13x10-43
Biochemistry	Serum calcium concentration†	KSD (FinnGen)	156	**1.31 (1.12-1.53)**	**1.48x10-3**	444.03	1.10x10-29	-6.73x10-3 (4.90x10-3)	0.17	1.56 (1.16-2.09)	3.66x10-3	438.65	3.79x10-29
Biochemistry	Serum calcium concentration†	KSD (Meta-analysis)	172	**1.42 (1.21-1.66)**	**1.51x10-5**	882.43	2.90x10-96	-3.77x10-3 (4.61x10-3)	0.41	1.56 (1.18-2.07)	2.21x10-3	878.98	5.15x10-96
Biochemistry	Serum calcium concentration†	Serum 25-OH vitamin D concentration	172	**0.98 (0.94-1.02)**	**0.27**	1124.32	6.40x10-140	2.24x10-3 (9.45x10-4)	0.02	0.92 (0.87-0.98)	9.86x10-3	1088.23	1.11x10-133
Biochemistry	Serum calcium concentration†	WHR	172	**1.03 (1.01-1.05)**	**0.02**	840.87	5.27x10-89	8.79x10-4 (7.78x10-4)	0.26	1.01 (0.96-1.06)	0.78	834.6	2.90x10-88
Biochemistry	Serum phosphate concentration	KSD (FinnGen)	84	**0.75 (0.61-0.92)**	**9.26x10-3**	215.89	8.90x10-14	-9.52x10-3 (5.85x10-3)	0.11	0.94 (0.67-1.31)	0.7	209.12	4.56x10-13
Biochemistry	Serum phosphate concentration	KSD (Meta-analysis)	87	**0.74 (0.61-0.9)**	**4.00x10-3**	354.52	2.68x10-34	-4.63x10-3 (5.49x10-3)	0.4	0.83 (0.59-1.17)	0.29	351.58	4.02x10-34
Metabolic syndrome	T2D	KSD (FinnGen)	307	**1.06 (1.02-1.1)**	**0.02**	386.5	1.21x10-3	2.85x10-3 (2.48x10-3)	0.25	1 (0.91-1.11)	0.93	384.83	1.29x10-3

†Albumin adjusted serum calcium concentration; APO-B= apolipoprotein-B; BMI = body mass index; CI=confidence interval; CRP= C-reactive protein; HDL = high density lipoprotein; LDL = low density lipoprotein; KSD = kidney stone disease; LDL = low density lipoprotein; N SNP= number of single nucleotide polymorphisms in analysis; OR=odds ratio for outcome per 1 standard deviation increase in genetically-instrumented exposure variable; p= p-value; p* = p value adjusted for multiple testing using 5% false discovery rate method; SE= standard error; T2D= type 2 diabetes; TG = triglyceride; WHR = waist-to-hip ratio; 25-OH vitamin D = hydroxyvitamin D. Bold text highlights the sensitivity analysis to be interpreted after considering the estimate of the intercept.

Supplementary Table 17: Multivariable Mendelian randomization analyses in UK Biobank

Analysis					Inverse variance weighted				Intercept		MR Egger			
					Estimate		Heterogeneity				Estimate		Heterogeneity	
Multivariable model	Exposure	Adjusted for	Outcome	N SNP	OR (95% CI)	p	Q	p	Beta (SE)	p	OR (95% CI)	p	Q	p
WHR & BMI	WHR	BMI	KSD (Females)	416	**1.4 0 (1.14-1.72)**	**2.46x10-3**	485.19	8.90x10-3	5.00x10-3 (3.00x10-3)	0.07	1.42 (1.16-1.75)	1.00x10-3	481.27	0.01
	BMI	WHR	KSD (Females)	416	**1.20 (0.98-1.48)**	**0.12**	485.19	8.90x10-3	5.00x10-3 (3.00x10-3)	0.07	0.98 (0.69-1.4)	0.62	481.27	0.01
	WHR	BMI	KSD (Males)	250	**1.79 (1.27-2.53)**	**2.46x10-3**	330.70	3.00x10-4	-2.00x10-3 (4.00x10-3)	0.60	1.81 (1.28-2.56)	1.00x10-3	330.33	3.00x10-4
	BMI	WHR	KSD (Males)	250	**0.89 (0.69-1.14)**	**0.42**	330.70	3.00x10-4	-2.00x10-3 (4.00x10-3)	0.60	0.96 (0.65-1.41)	0.82	330.33	3.00x10-4
	WHR	BMI	KSD	777	**1.41 (1.2-1.67)**	**1.14x10-4**	978.38	4.48x10-5	-2.38x10-4 (1.70x10-3)	0.89	1.43 (1.11-1.85)	6.00x10-3	978.36	7.48x10-7
	BMI	WHR	KSD	777	**1.22 (1.07-1.39)**	**5.70x10-3**	978.38	4.48x10-5	-2.38x10-4 (1.70x10-3)	0.89	1.23 (1.05-1.43)	9.00x10-3	978.36	7.48x10-7
WC & BMI	WC	BMI	KSD (Females)	231	**1.36 (0.7-2.65)**	**0.43**	266.08	0.05	7.00x10-3 (4.00x10-3)	0.07	1.40 (0.72-2.73)	0.31	262.20	0.06
	BMI	WC	KSD (Females)	231	**0.95 (0.5-1.78)**	**0.87**	266.08	0.05	7.00x10-3 (4.00x10-3)	0.07	0.65 (0.31-1.37)	0.26	262.20	0.06
	WC	BMI	KSD (Males)	233	**1.09 (0.71-1.66)**	**0.74**	317.48	4.00x10-4	2.56x10-4 (4.00x10-3)	0.95	1.09 (0.71-1.67)	0.7	317.48	4.00x10-4
	BMI	WC	KSD (Males)	233	**1.20 (0.83-1.76)**	**0.41**	317.48	4.00x10-4	2.56x10-4 (4.00x10-3)	0.95	1.19 (0.71-1.98)	0.50	317.48	4.00x10-4
	WC	BMI	KSD	577	**1.16 (0.86-1.56)**	**0.38**	699.6	2.73x10-4	1.00x10-3 (2.00x10-3)	0.51	1.13 (0.83-1.54)	0.43	699.08	2.75x10-4
	BMI	WC	KSD	577	**1.26 (0.98-1.64)**	**0.11**	699.6	2.73x10-4	1.00x10-3 (2.00x10-3)	0.51	1.21 (0.9-1.62)	0.22	699.08	2.75x10-4
Serum calcium concentration† & BMI & WHR	Serum calcium concentration†	WHR and BMI	KSD	891	1.53 (1.33-1.75)	1.38x10-8	1561.99	8.65x10-40	-2.00x10-3 (1.00x10-4)	0.01	**1.71 (1.46-2.02)**	**1.07x10-10**	1551.47	6.35x10-39
	WHR	Serum calcium concentration† and BMI	KSD	891	1.39 (1.14-1.7)	2.47x10-3	1561.99	8.65x10-40	-2.00x10-3 (1.00x10-4)	0.01	**1.42 (1.16-1.74)**	**1.00x10-3**	1551.47	6.35x10-39
	BMI	Serum calcium concentration† and WHR	KSD	891	1.18 (1.01-1.37)	0.06	1561.99	8.65x10-40	-2.00x10-3 (1.00x10-4)	0.01	**1.19 (1.02-1.39)**	**0.03**	1551.47	6.35x10-39
Serum calcium concentration† & Serum 25 OH vitamin D	Serum calcium concentration†	Serum 25 OH vitamin D	KSD	235	**1.62 (1.34-1.96)**	**2.74x10-6**	888.67	2.96x10-77	-7.00x10-3 (5.00x10-3)	0.13	2.06 (1.43-2.96)	9.43x10-5	879.82	3.99x10-76
	Serum 25 OH vitamin D	Serum calcium concentration†	KSD	235	**1.13 (0.76-1.69)**	**0.57**	888.67	2.96x10-77	-7.00x10-3 (5.00x10-3)	0.13	1.17 (0.78-1.73)	0.45	879.82	3.99x10-76
T2D & BMI & WHR	T2D	WHR and BMI	KSD	614	**0.98 (0.93-1.03)**	**0.42**	866.38	4.03x10-11	1.00x10-3 (2.00x10-3)	0.60	0.96 (0.88-1.05)	0.36	866	3.57x10-11
	WHR	T2D and BMI	KSD	614	**1.79 (1.43-2.24)**	**1.80x10-6**	866.38	4.03x10-11	1.00x10-3 (2.00x10-3)	0.60	1.79 (1.43-2.24)	3.97x10-7	866	3.57x10-11
	BMI	T2D and WHR	KSD	614	**1.1 (0.92-1.3)**	**0.36**	866.38	4.03x10-11	1.00x10-3 (2.00x10-3)	0.60	1.1 (0.92-1.31)	0.30	866	3.57x10-11
Multivariable model	Exposure	Adjusted for	Outcome	N SNP	Beta	p	Q	p	Beta	p	Beta	p	Q	p

					(SE)				(SE)		(SE)			
BMI & WHR	BMI	WHR	Serum calcium concentration†	776	**-0.03 (0.02)**	**0.19**	2655.21	4.02x10-209	-1.16x10-4 (4.06x10-4)	0.77	-0.02 (0.03)	0.54	26549291	2.40x10-204
	WHR	BMI	Serum calcium concentration†	776	**0.13 (0.02)**	**2.77x10-8**	2655.21	4.02x10-209	-1.16x10-4 (4.06x10-4)	0.77	0.13 (0.02)	3.50x10-9	26549291	2.40x10-204

†Albumin adjusted serum calcium concentration; APO-B= apolipoprotein-B; BMI = body mass index; CI=confidence interval; CRP= C-reactive protein; HDL = high density lipoprotein; LDL = low density lipoprotein; KSD = kidney stone disease; LDL = low density lipoprotein; N SNP= number of single nucleotide polymorphisms in analysis; OR=odds ratio for outcome per 1 standard deviation increase in genetically-instrumented exposure variable; p* = p value adjusted for multiple testing using 5% false discovery rate method; SE= standard error; T2D= type 2 diabetes; TG = triglyceride; WHR = waist-to-hip ratio; 25-OH vitamin D = hydroxyvitamin D. Bold text highlights the sensitivity analysis to be interpreted after considering the estimate of the intercept.

Supplementary Table 18: Multivariable Mendelian randomization analyses in FinnGen study

Analysis					Inverse variance weighted				Intercept		MR Egger			
					Estimate		Heterogeneity				Estimate		Heterogeneity	
Multivariable model	Exposure	Adjusted for	Outcome	N SNP	OR (95% CI)	p	Q	p	Beta (SE)	p	OR (95% CI)	p	Q	p
WHR & BMI	WHR	BMI	KSD	759	**1.15 (0.96-1.38)**	**0.19**	1066.31	6.95x10-13	-0.02 (0.02)	0.34	1.27 (0.96-1.69)	0.09	1065.05	6.70x10-13
	BMI	WHR	KSD	759	**1.2 (1.04-1.38)**	**0.11**	1066.31	6.95x10-13	-0.02 (0.02)	0.34	1.19 (1-1.4)	0.05	1065.05	6.70x10-13
WC & BMI	WC	BMI	KSD	568	**1.19 (0.86-1.65)**	**0.36**	773.71	1.30x10-8	-1.00x10-3 (2.00x10-3)	0.59	1.21 (0.87-1.69)	0.25	773.31	1.31x10-8
	BMI	WC	KSD	568	**1.08 (0.82-1.44)**	**0.60**	773.71	1.30x10-8	-1.00x10-3 (2.00x10-3)	0.59	1.13 (0.82-1.56)	0.46	773.31	1.31x10-8
Serum calcium concentration† & BMI & WHR	Serum calcium concentration†	WHR and BMI	KSD	866	**1.53 (1.33-1.77)**	**2.66x10-8**	1554.31	3.32x10-42	-2.00x10-3 (1.00x10-3)	0.13	1.65 (1.39-1.95)	5.89x10-9	1550.18	6.22x10-42
	WHR	Serum calcium concentration† and BMI	KSD	866	**1.08 (0.87-1.33)**	**0.52**	1554.31	3.32x10-42	-2.00x10-3 (1.00x10-3)	0.13	1.09 (0.88-1.35)	0.41	1550.18	6.22x10-42
	BMI	Serum calcium concentration† and WHR	KSD	866	**1.16 (0.99-1.37)**	**0.11**	1554.31	3.32x10-42	-2.00x10-3 (1.00x10-3)	0.13	1.17 (0.99-1.38)	0.06	1550.18	6.22x10-42
Serum calcium concentration† & Serum 25 OH vitamin D	Serum calcium concentration†	Serum 25 OH vitamin D	KSD	216	**1.53 (1.27-1.84)**	**1.84x10-5**	721.6	2.94x10-56	-8.00x10-3 (4.00x10-3)	0.06	2.01 (1.43-2.83)	5.59x10-5	709.88	9.99x10-55
	Serum 25 OH vitamin D	Serum calcium concentration†	KSD	216	**0.96 (0.67-1.39)**	**0.85**	721.6	2.94x10-56	-8.00x10-3 (4.00x10-3)	0.06	0.99 (0.69-1.42)	0.95	709.88	9.99x10-55
T2D & BMI & WHR	T2D	WHR and BMI	KSD	595	**0.98 (0.93-1.03)**	**0.46**	928.36	2.52x10-17	1.00x10-3 (2.00x10-3)	0.60	0.96 (0.87-1.05)	0.38	927.92	2.17x10-17
	WHR	T2D and BMI	KSD	595	**1.37 (1.07-1.77)**	**0.02**	928.36	2.52x10-17	1.00x10-3 (2.00x10-3)	0.60	1.38 (1.07-1.77)	0.01	927.92	2.17x10-17
	BMI	T2D and WHR	KSD	595	**1.08 (0.89-1.31)**	**0.46**	928.36	2.52x10-17	1.00x10-3 (2.00x10-3)	0.60	1.08 (0.89-1.31)	0.41	927.92	2.17x10-17

†Albumin adjusted serum calcium concentration; APO-B= apolipoprotein-B; BMI = body mass index; CI=confidence interval; CRP= C-reactive protein; HDL = high density lipoprotein; LDL = low density lipoprotein; KSD = kidney stone disease; LDL = low density lipoprotein; N SNP= number of single nucleotide polymorphisms in analysis; OR=odds ratio for outcome per 1 standard deviation increase in genetically-instrumented exposure variable; p* = p value adjusted for multiple testing using 5% false discovery rate method; SE= standard error; T2D= type 2 diabetes; TG = triglyceride; WHR = waist-to-hip ratio; 25-OH vitamin D = hydroxyvitamin D. Bold text highlights the sensitivity analysis to be interpreted after considering the estimate of the intercept.

Supplementary Table 19: Observational associations of the effects of waist-to-hip ratio on albumin-adjusted serum calcium concentrations in UK Biobank.

	**Females (n=263,248)**	**Males (n=216,157)**
Mean WHR (SD)	0.82 (0.07)	0.94 (0.06)
Effect of 1SD increase in WHR on albumin-adjusted serum calcium concentrations	0.09 mmol/L	0.05 mmol/L
95% Confidence interval	0.086-0.096	0.047-0.057
Statistical significance	$p=2.90x10^{-284}$	$p=2.06x10^{-79}$

SD= Standard deviation; WHR = waist-to-hip ratio

Supplementary Table 20: Summary of Mendelian randomisation estimates for type 2 diabetes and kidney stone disease.

**MR analysis**		**UK Biobank**	**FinnGen**	**Meta-analysis of UK Biobank and FinnGen**
	**N SNP**	367	351	354
**Inverse-variance weighted**	**OR (95% CI)**	1.12 (1.06-1.17)	1.08 (1.02-1.13)	1.09 (1.05-1.14)
	**P***	5.06x10-5	0.03	1.49x10-4
**Intercept**	**Beta (SE)**	5.82x10-3 (2.45x10-3)	2.35x10-3 (2.76x10-3)	4.65x10-3 (2.17x10-3)
	**P**	0.02	0.39	0.03
**MR-Egger**	**OR (95% CI)**	1 (0.91-1.11)	1.03 (0.92-1.15)	1.01 (0.92-1.1)
	**P**	0.96	0.61	0.9
**Weighted median**	**OR (95% CI)**	1.05 (0.99-1.12)	1.05 (0.99-1.12)	1.04 (0.99-1.1)
	**P**	0.2	0.2	0.22
**Contamination mix**	**OR (95% CI)**	1.07 (0.98-1.16)	1.03 (0.96-1.11)	1.1 (1.06-1.15)
	**P**	0.1	0.45	4.49x10-3

CI=confidence interval; OR=odds ratio for outcome per 1 standard deviation increase in genetically-instrumented exposure variable; N SNP= number of single nucleotide polymorphisms in analysis; p* = p value adjusted for multiple testing using 5% false discovery rate method; SE= standard error

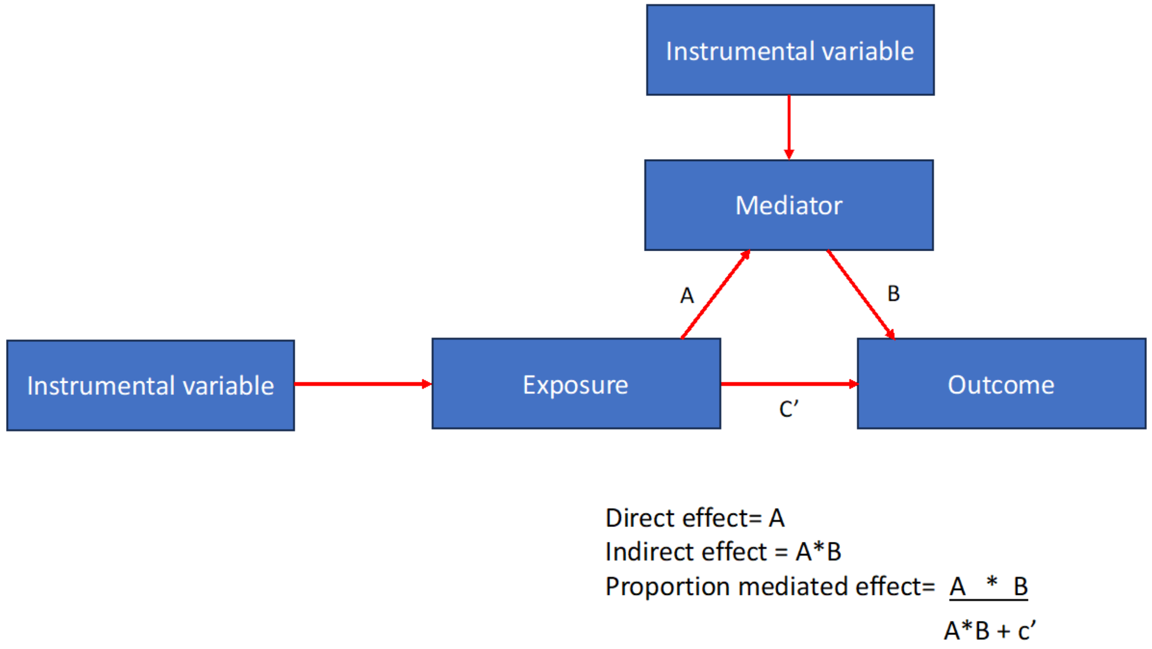

Supplementary Figure 1: Methods for mediation Mendelian randomisation

As described by Carter AR, Sanderson E, Hammerton G, Richmond RC, Davey Smith G, Heron J, et al. Mendelian randomisation for mediation analysis: current methods and challenges for implementation. European journal of epidemiology. May 2021;36(5):465-478. doi:10.1007/s10654-021-00757-1

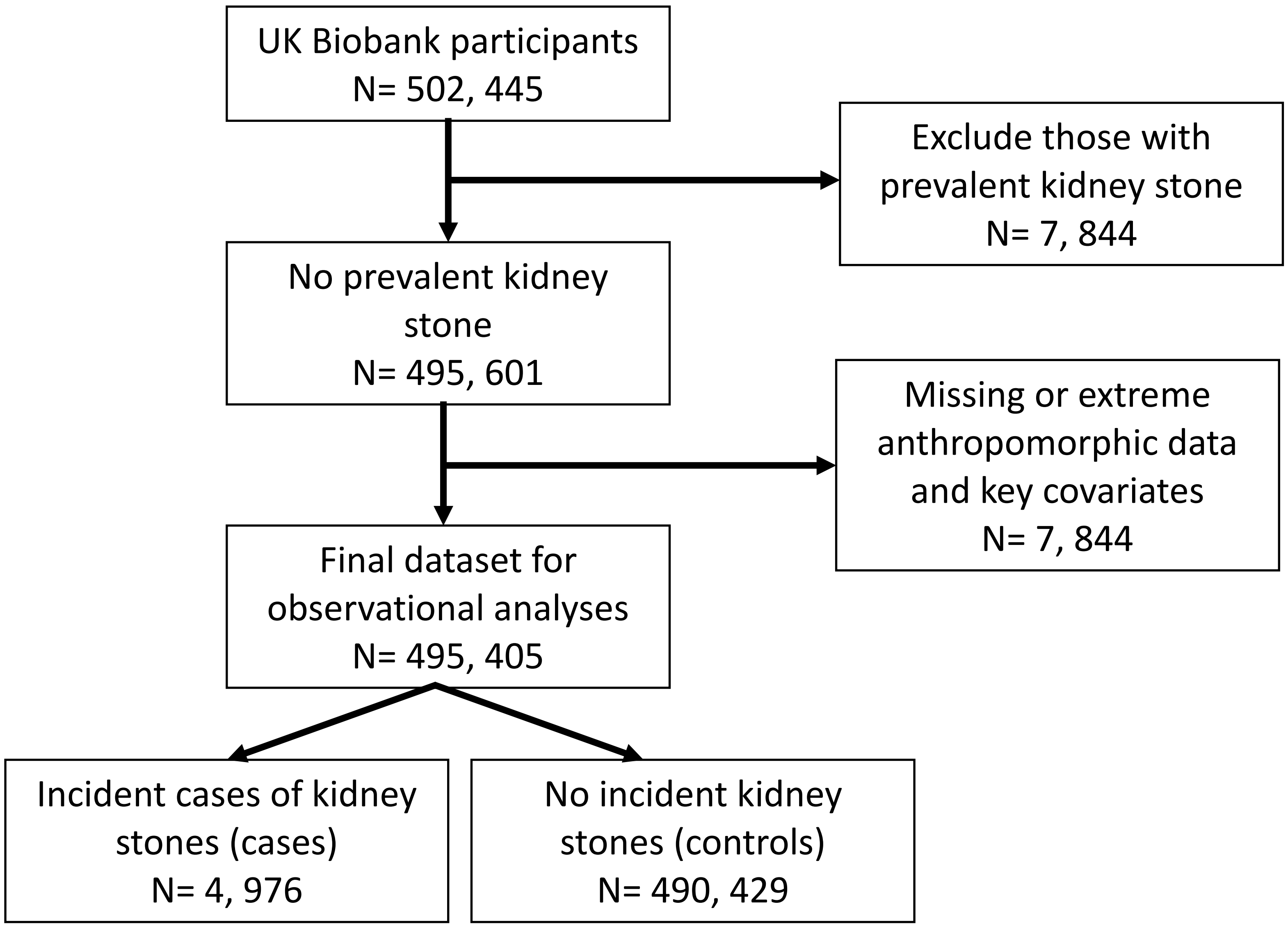

Supplementary Figure 2: Derivation of UK Biobank cohort for observational analyses

Anthropometric measures	Model	SD	HR (95% CI)	χ^2	SD	HR (95% CI)	χ^2
			Male			Female	
**BMI**	Standard Adjustment	4.04	1.28 (1.24 - 1.32)	210.5	4.94	1.25 (1.20 - 1.30)	98.8
	+WHR		1.20 (1.15 - 1.25)	71.8		1.14 (1.08 - 1.19)	25.4
	+WC		1.14 (1.06 - 1.22)	12.9		0.92 (0.83 - 1.00)	3.5
**WHR**	Standard Adjustment	0.05	1.33 (1.28 - 1.39)	169.3	0.06	1.43 (1.36 - 1.51)	160.2
	+BMI		1.16 (1.10 - 1.22)	30.6		1.34 (1.26 - 1.42)	86.8
**WC**	Standard Adjustment	10.16	1.31 (1.27 - 1.36)	210.8	11.34	1.35 (1.29 - 1.41)	151.9
	+BMI		1.16 (1.07 - 1.25)	12.9		1.47 (1.33 - 1.62)	56.7

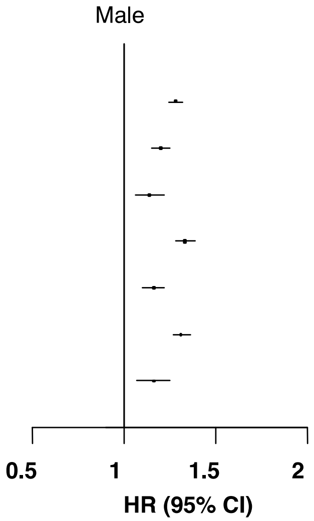

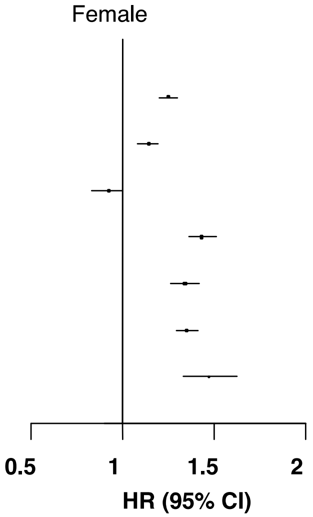

Supplementary Figure 3: Hazard ratio (HR) for incident kidney stone disease (KSD) and 95% confidence interval (CI) per standard deviation (SD) change in body mass index (BMI), waist-to-hip ratio (WHR) and waist circumference (WC) in observational analysis in the UK Biobank

HR per SD stratified by age-at-risk, and ethnicity, and adjusted for Townsend Deprivation Index, smoking and alcohol drinking (standard adjustments), with further adjustments for other anthropometric measures where indicated. Analyses exclude participants with pre-existing KSD (or conditions known to predispose to KSD) at baseline, and those with missing or outlying values in anthropometric variables or key covariates, leaving 478,405 participants. The variance of the category-specific log risk determines the CI.

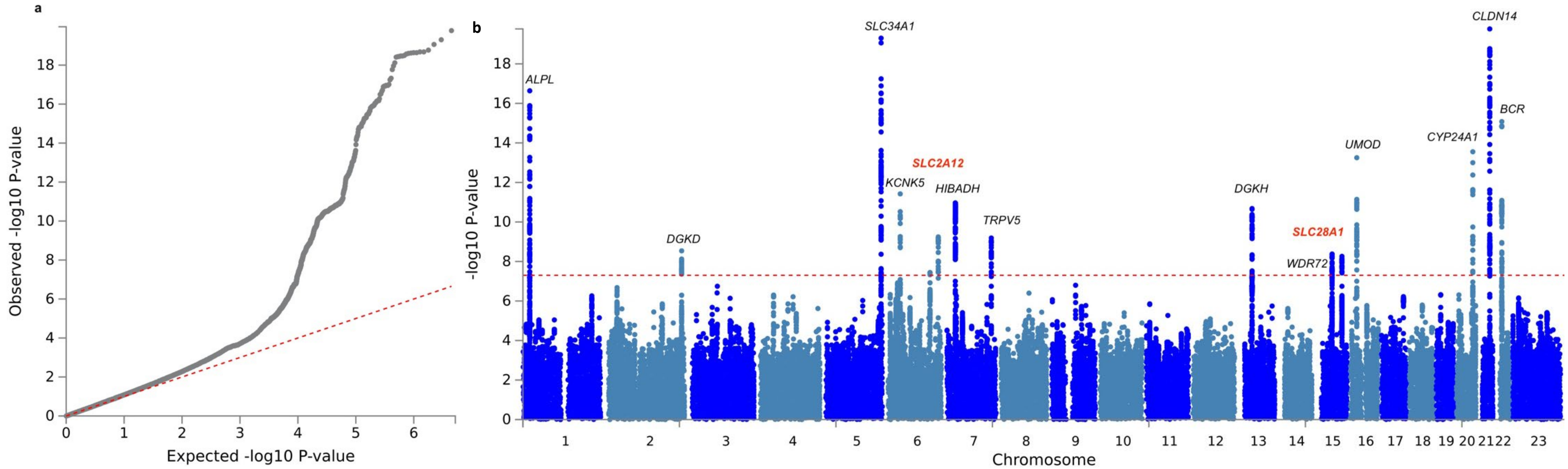

Supplementary Figure 4: Results of combined sex genome-wide association study (GWAS) in kidney stone disease (KSD) in UK Biobank.

GWAS of KSD was performed for 8,504 individuals with KSD and 318,819 controls from the UK Biobank. **a** is a quantile-quantile plot of observed vs. expected p-values. The λGC demonstrated some inflation (1.001), but the LD score regression (LDSC) intercept of 1.01, with an attenuation ratio of 0.31 indicated that the inflation was largely due to polygenicity and the large sample size. **b is** a Manhattan plot showing the genome-wide p values (-log10) plotted against their respective positions on each of the chromosomes. The horizontal red line indicates the genome-wide significance threshold of 5.0 x 10^{-8}. Loci have been labelled with the primary candidate gene at each locus, as shown in Table 2. Previously unreported GWAS-discovered kidney stone loci are highlighted in red.

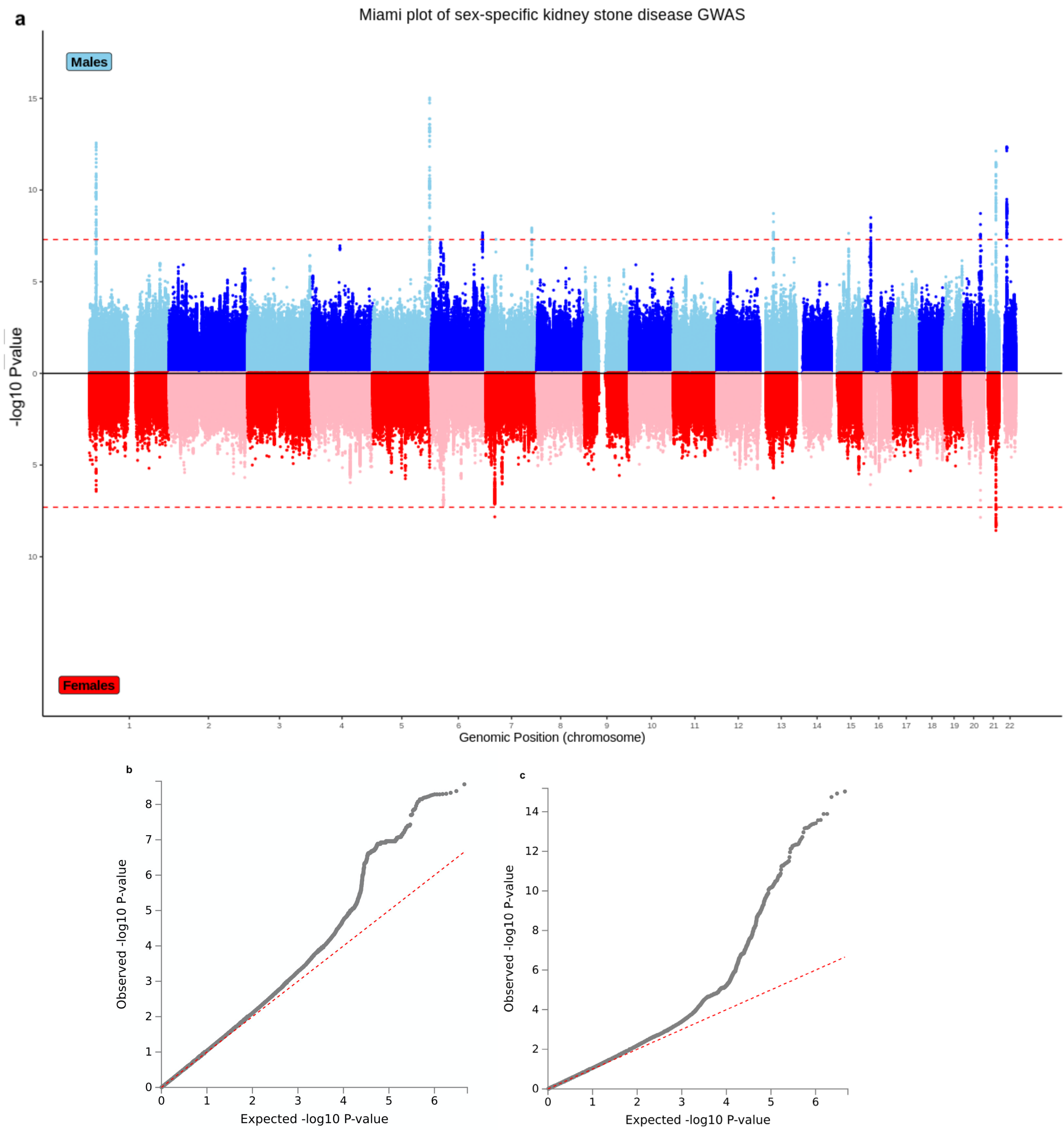

Supplementary Figure 5: Results of sex-specific genome wide association study in kidney stone disease in the UK Biobank

a Miami plot illustrating the results of sex-specific genome-wide association studies (GWAS) in kidney stone disease (KSD) in the UK Biobank. Analysis of genetically male individuals included 5,633 cases and 176,738 controls, blue. Analysis of genetically female individuals included 2,871 cases and 212,081 controls, red. Genome-wide p values (-log10) are plotted against their respective positions on each of the autosomes. The horizontal red line shows the genome-wide significance threshold of 5.0 x 10−8. **b** Quantile-quantile plot of observed vs. expected p-values for female-specific GWAS of KSD. λGC= 1.05, LD score regression (LDSC) intercept=1.003, attenuation ratio =0.283. **c** Quantile-quantile plot of observed vs. expected p-values for male-specific GWAS of KSD. λGC= 1.05, LD score regression (LDSC) intercept=1.005, attenuation ratio =0.147

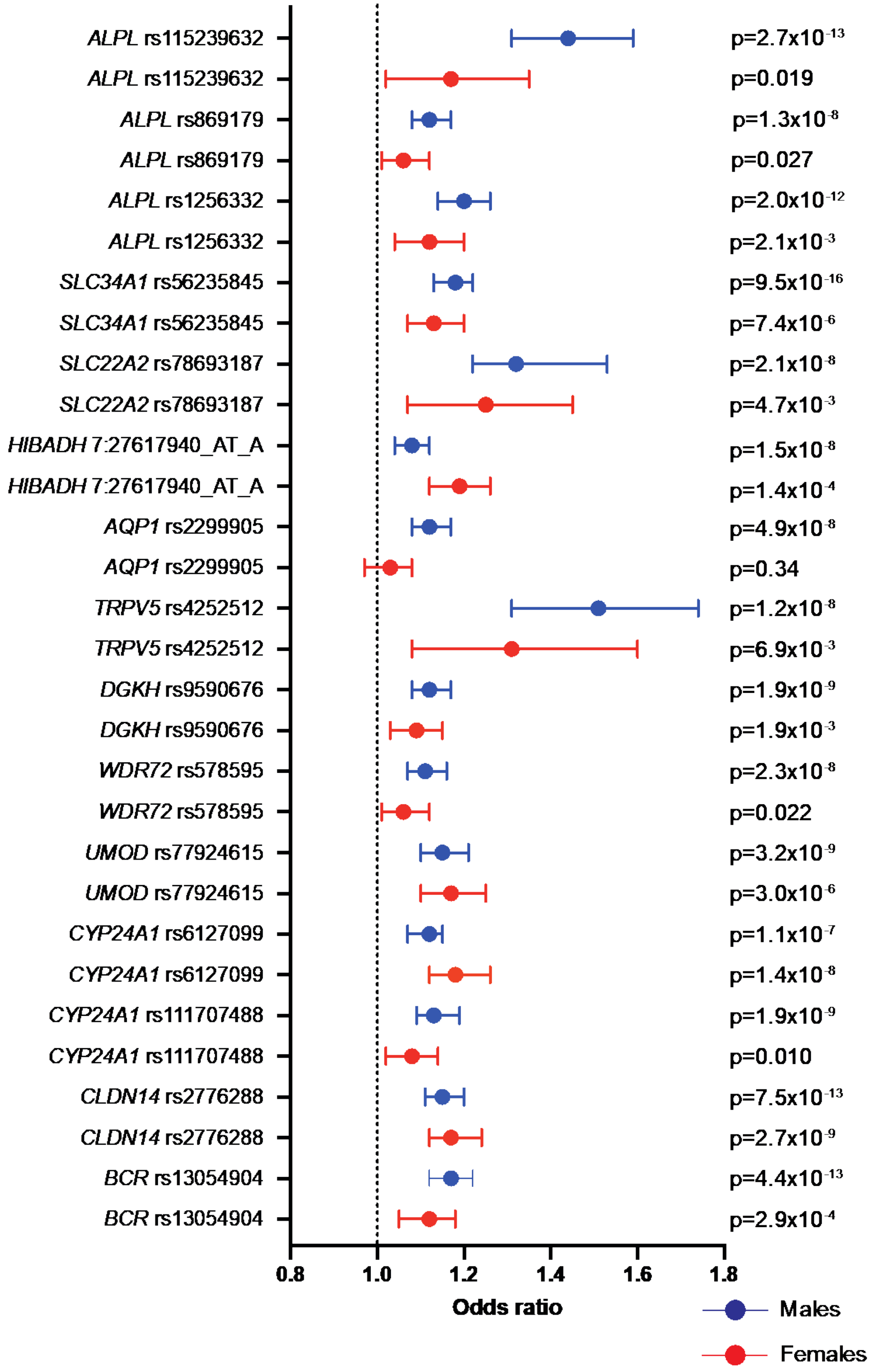

Supplementary Figure 6: Sex-specific odds ratios, 95% confidence intervals and p values for significant hits in genome wide association studies in UK Biobank of males or females.
Male data is represented in blue, female data is represented in red.

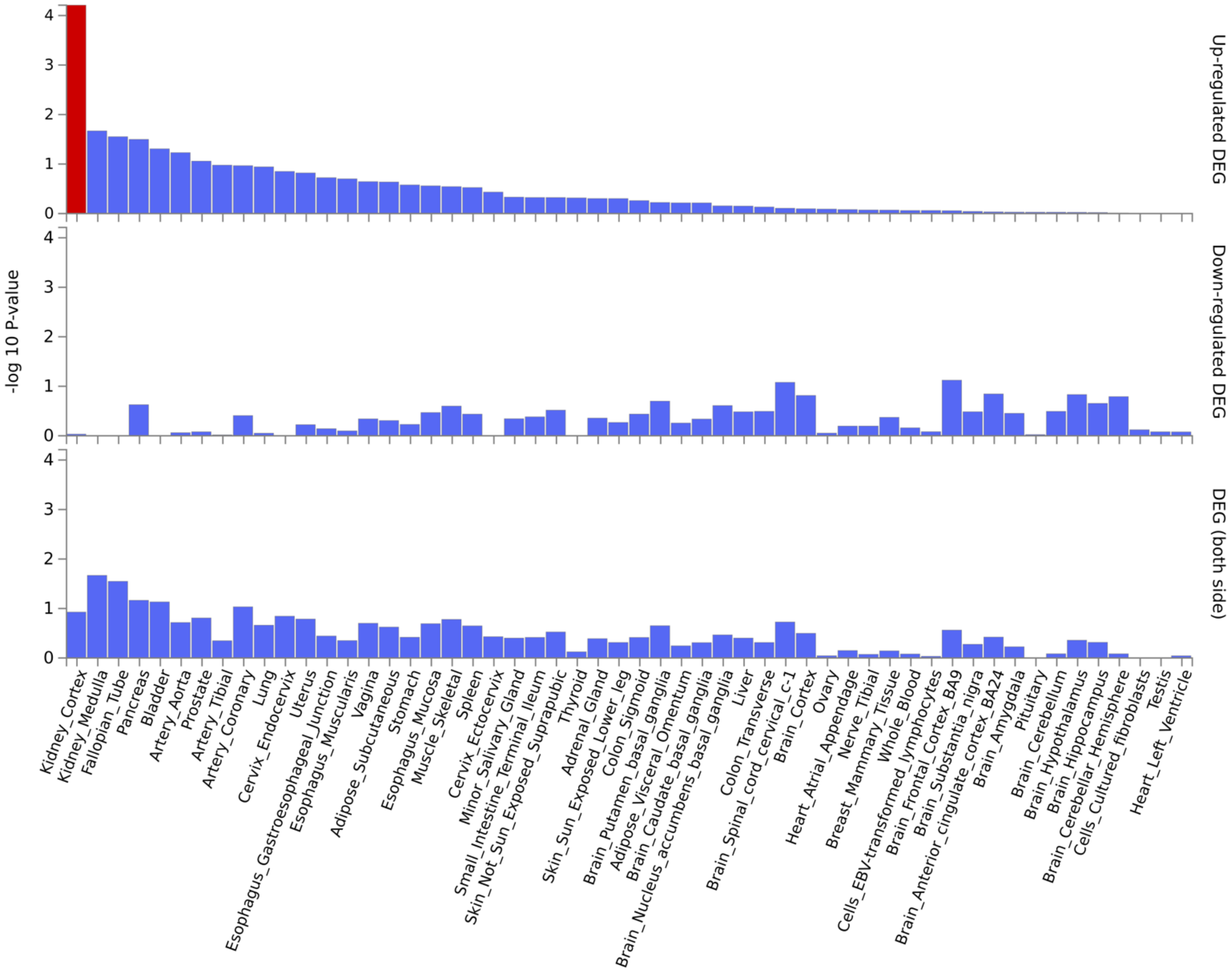

Supplementary Figure 7: Differential gene expression across 54 tissue types in GTEx v8.

Expression analysis of GWAS-meta-analysis summary data, implemented in FUMA. This tests the relationship between highly expressed genes in a specific tissue and the genetic associations from the GWAS summary statistics. Gene-property analysis is performed using average expression of genes per tissue type as a gene covariate. Gene expression values are log2 transformed average RPKM (Read Per Kilobase Per Million) per tissue type after winsorization at 50, and are based on GTEx v8 RNA-Seq data across 54 tissue types.

Tissues that meet Bonferroni-corrected α-level significance threshold are highlighted in red.

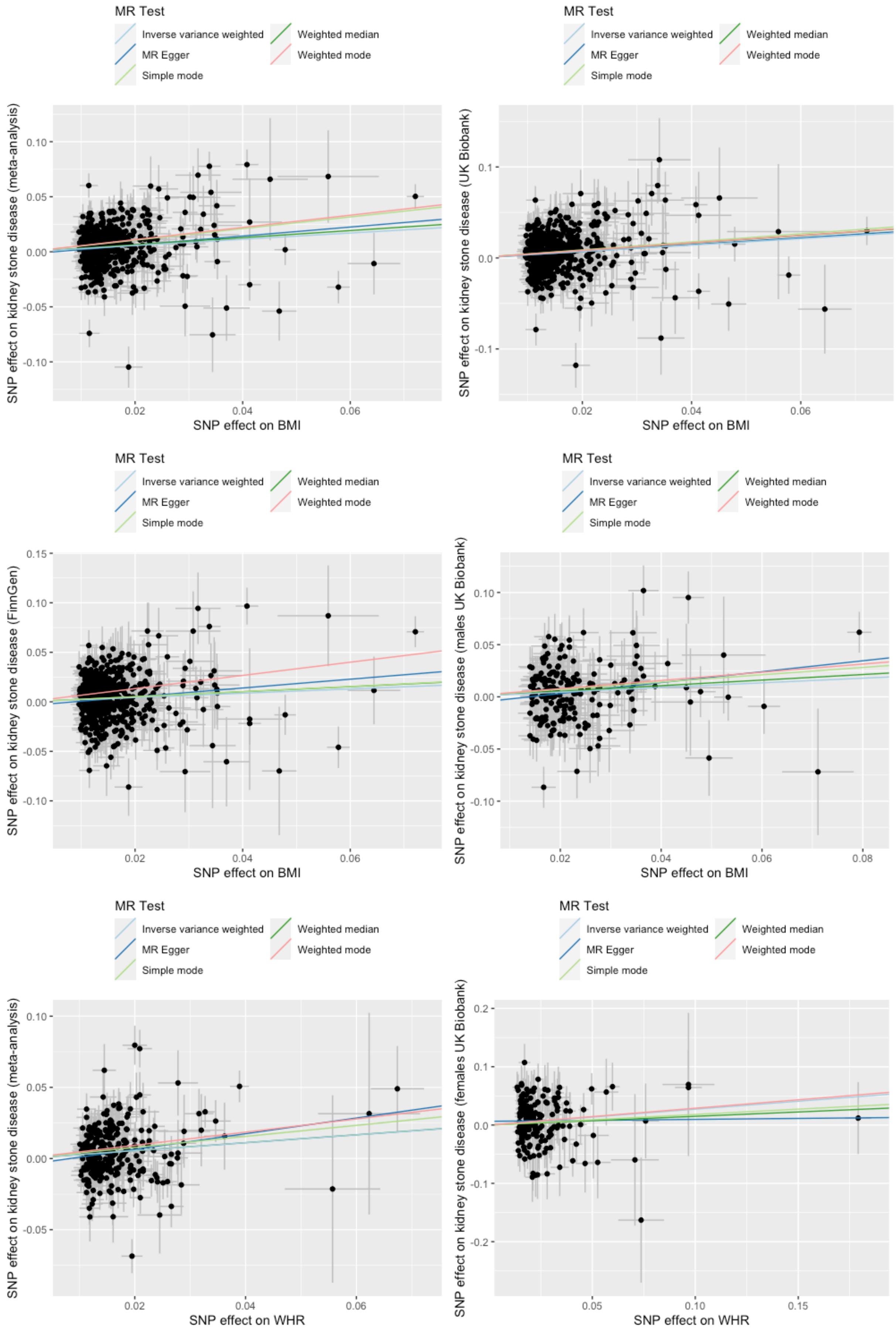
MR Test
Inverse variance weighted
MR Egger
Simple mode
Weighted median
Weighted mode
SNP effect on kidney stone disease (meta-analysis)
SNP effect on BMI
SNP effect on kidney stone disease (UK Biobank)
SNP effect on BMI
SNP effect on kidney stone disease (FinnGen)
SNP effect on BMI
SNP effect on kidney stone disease (males UK Biobank)
SNP effect on BMI
SNP effect on kidney stone disease (meta-analysis)
SNP effect on WHR
SNP effect on kidney stone disease (females UK Biobank)
SNP effect on WHR

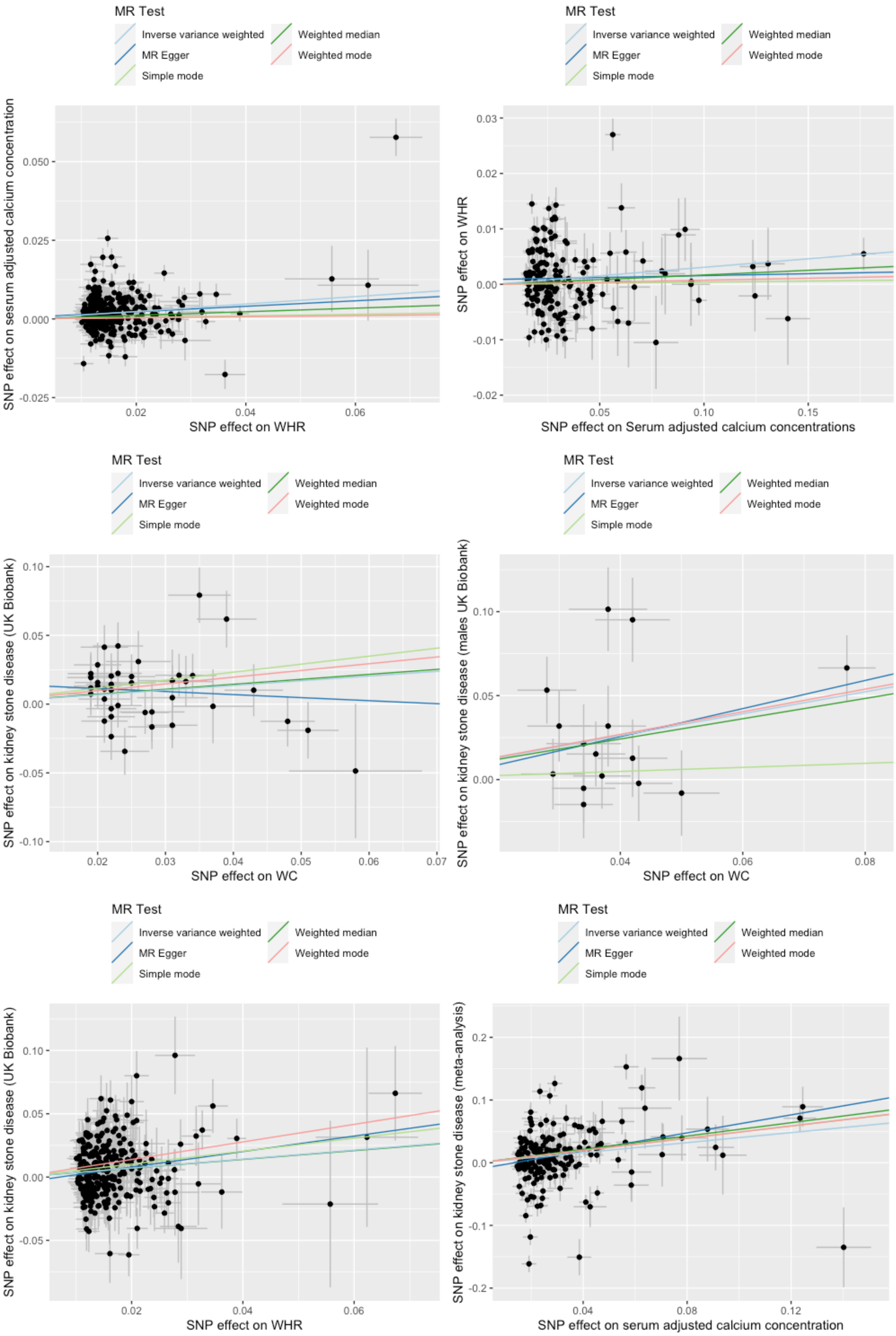
MR Test
Inverse variance weighted
Weighted median
MR Egger
Weighted mode
Simple mode
SNP effect on sesrum adjusted calcium concentration
SNP effect on WHR
SNP effect on WHR
SNP effect on Serum adjusted calcium concentrations
SNP effect on kidney stone disease (UK Biobank)
SNP effect on WC
SNP effect on kidney stone disease (males UK Biobank)
SNP effect on WC
SNP effect on kidney stone disease (UK Biobank)
SNP effect on WHR
SNP effect on kidney stone disease (meta-analysis)
SNP effect on serum adjusted calcium concentration

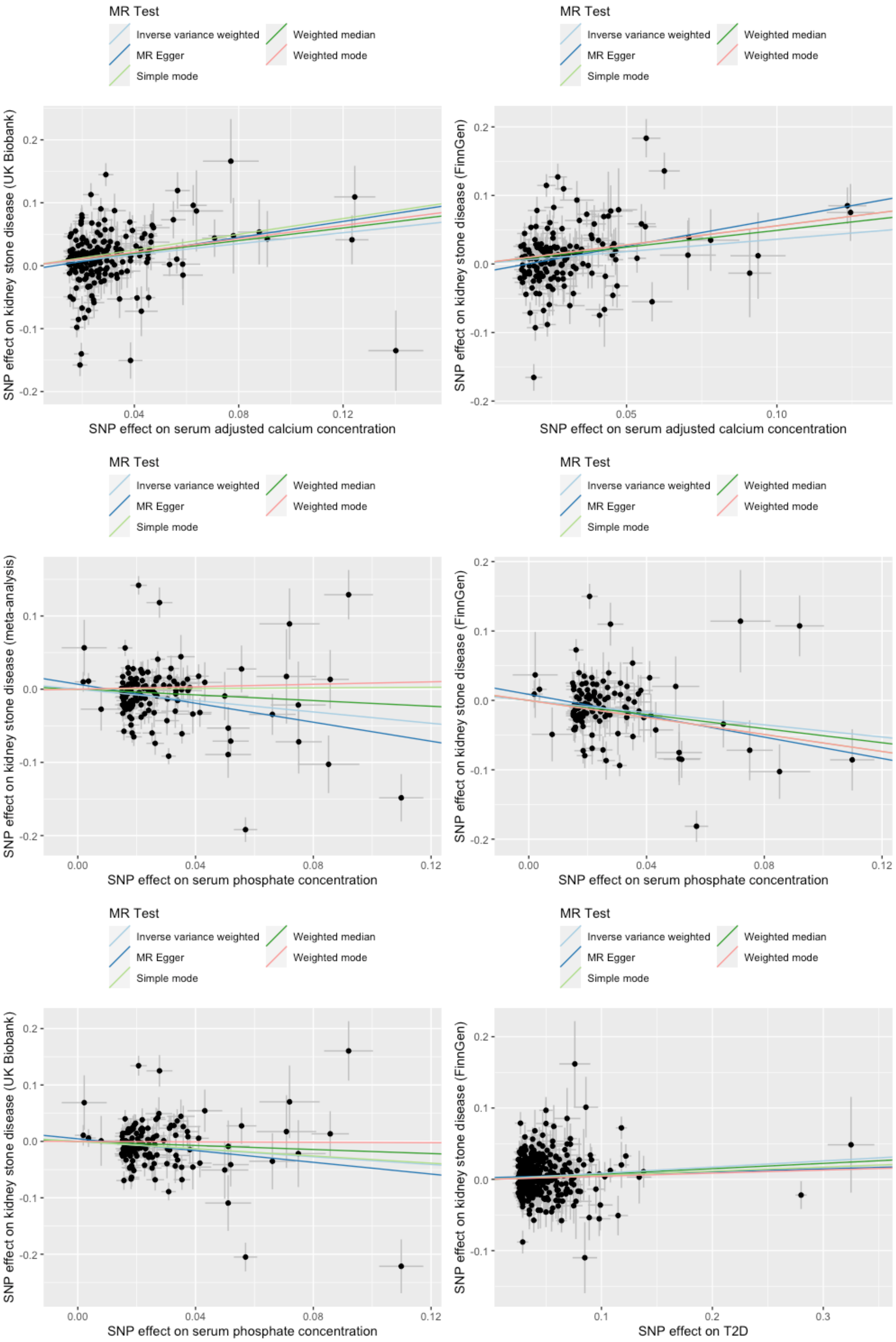
MR Test
Inverse variance weighted
MR Egger
Simple mode
Weighted median
Weighted mode
SNP effect on kidney stone disease (UK Biobank)
SNP effect on serum adjusted calcium concentration
SNP effect on kidney stone disease (FinnGen)
SNP effect on serum adjusted calcium concentration
SNP effect on kidney stone disease (meta-analysis)
SNP effect on serum phosphate concentration
SNP effect on kidney stone disease (FinnGen)
SNP effect on serum phosphate concentration
SNP effect on kidney stone disease (UK Biobank)
SNP effect on serum phosphate concentration
SNP effect on kidney stone disease (FinnGen)
SNP effect on T2D

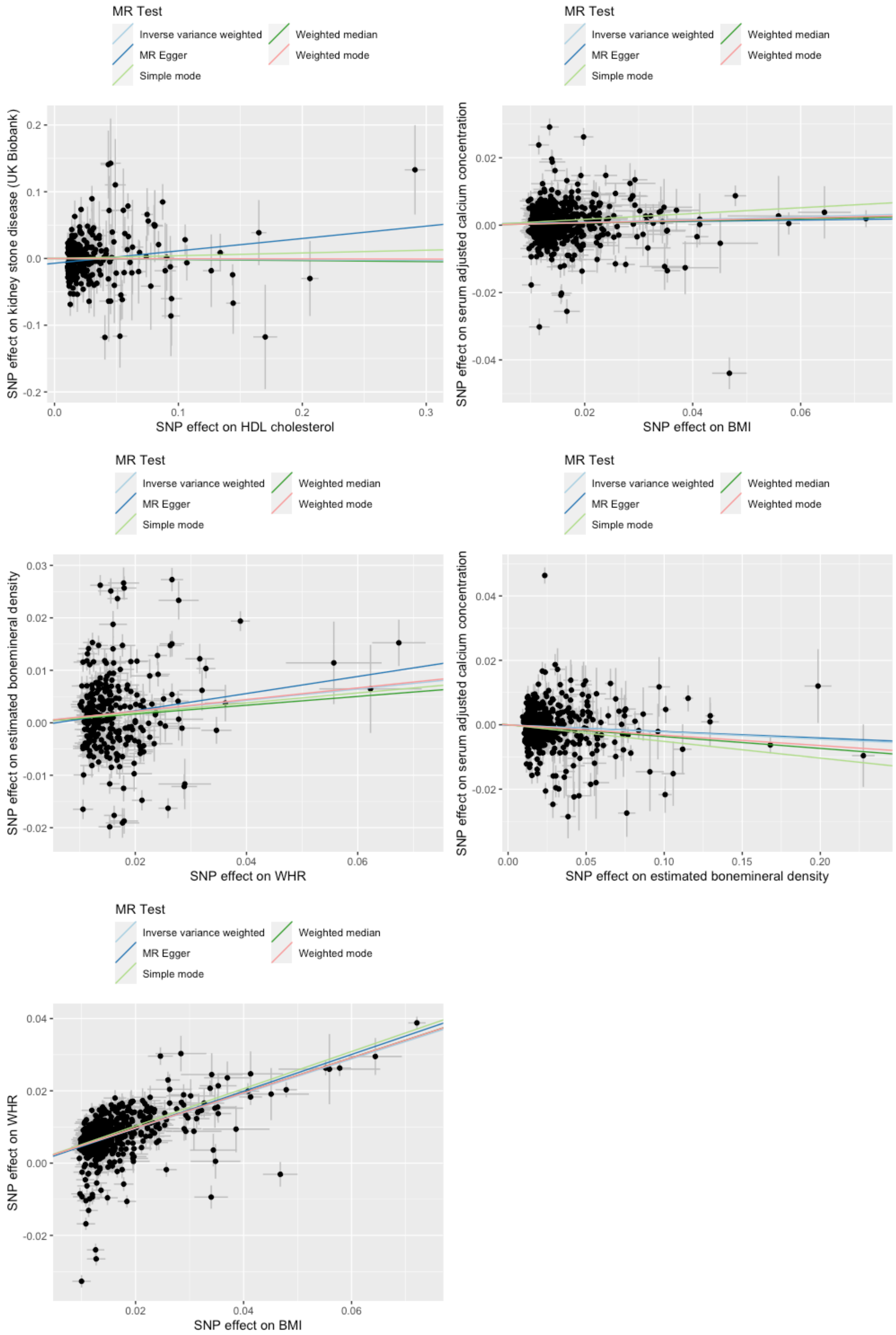

Supplementary Figure 8: Significant relationships identified on Mendelian randomisation.

Scatter plots of significant anthropometric, biochemical, and metabolic exposure variables verses kidney stone disease in meta-analysis, UK Biobank and FinnGen Mendelian randomisation analyses. Regression estimates are shown by coloured lines described in each figure legend. BMI= body mass index, WC= waist circumference, WHR= waist-to-hip ratio

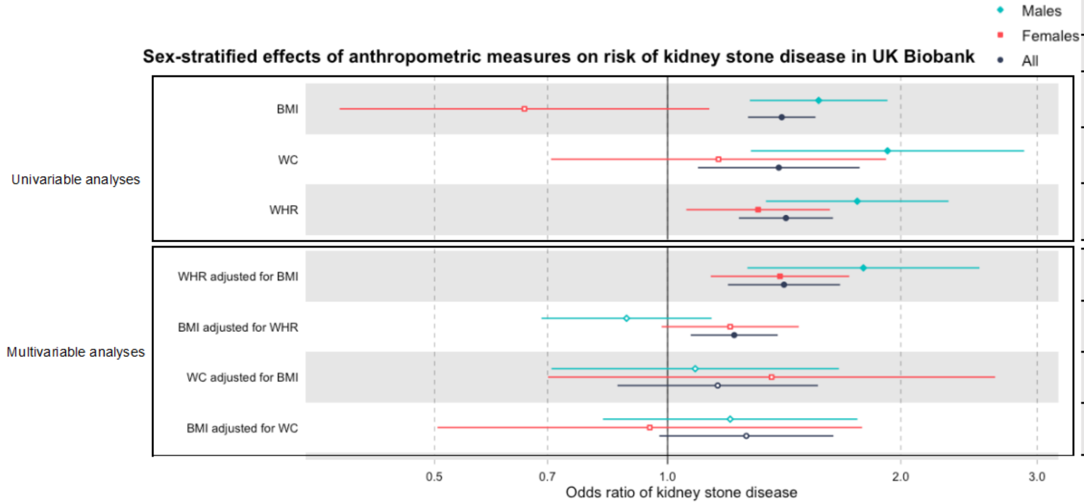

	Males			Females			All (combined-sex analysis)		
	OR	95% CI	P	OR	95% CI	P	OR	95% CI	P
BMI	1.57	(1.28-1.92)	1.31x10-4	0.65†	(0.38-1.13)	0.13	1.45	(0.93-2.27)	0.21
WC	1.92	(1.28-2.89)	1.88x10-5	1.16	(0.71-1.91)	0.73	1.42	(1.24-1.63)	1.28x10-5
WHR	1.76	(1.34-2.3)	2.99x10-4	1.31	(1.06-1.62)	0.03	1.15	(0.85-1.55)	1.28x10-5
WHR adjusted for BMI	1.79	(1.27-2.53)	2.46x10-3	1.40	(1.14-1.72)	2.46x10-3	1.41	(1.2-1.67)	1.14x10-4
BMI adjusted for WHR	0.89	(0.69-1.14)	0.42	1.20	(0.98-1.48)	0.12	1.22	(1.07-1.39)	5.70x10-3
WC adjusted for BMI	1.09	(0.71-1.66)	0.74	1.36	(0.7-2.65)	0.43	1.16	(0.86-1.56)	0.38
BMI adjusted for WC	1.20	(0.83-1.76)	0.41	0.95	(0.5-1.78)	0.87	1.26	(0.98-1.64)	0.11

Supplementary Figure 9. Sex-specific Mendelian randomisation estimates for adiposity-related exposure variables on risk of kidney stone disease.

Odds ratios (OR) and 95% confidence intervals (95% CI) of kidney stone disease per 1-standard deviation higher genetically-instrumented exposure variable. Non-significant results are displayed as hollow points. Estimates by default refer to inverse-variance weighted (IVW) estimates; where MR-Egger estimate has been used this is indicated by † annotation. BMI= body mass index, WC= waist circumference, WHR= waist-to-hip ratio.

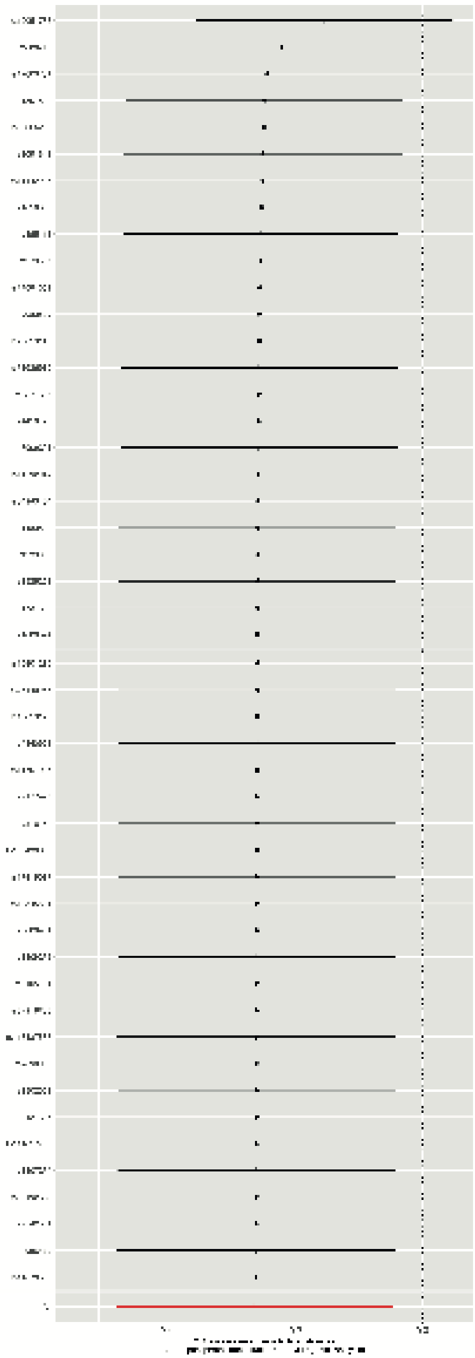

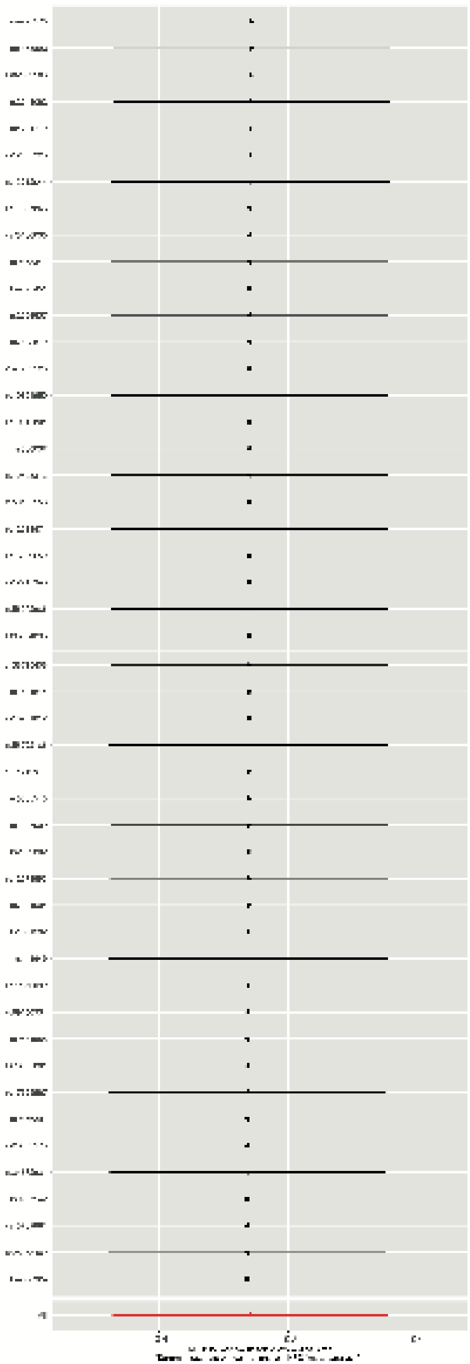

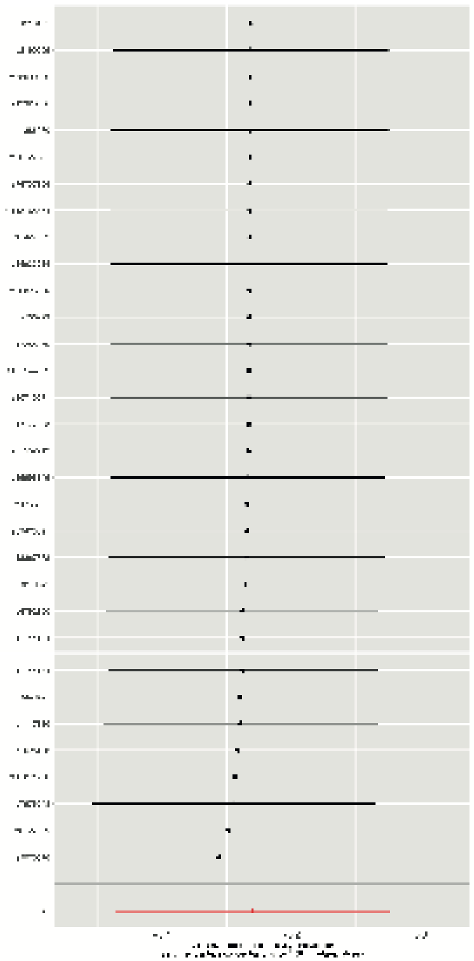

Supplementary Figure 10. Mendelian randomisation leave-one-out analyses for the effects of serum phosphate concentrations on risk of kidney stone disease in UK Biobank-FinnGen meta-analysis.

The x-axis reflects the log-odds ratio for kidney stone disease. All= overall inverse-variance weighted Mendelian randomisation estimate; KSD= kidney stone disease; MR= Mendelian randomisation

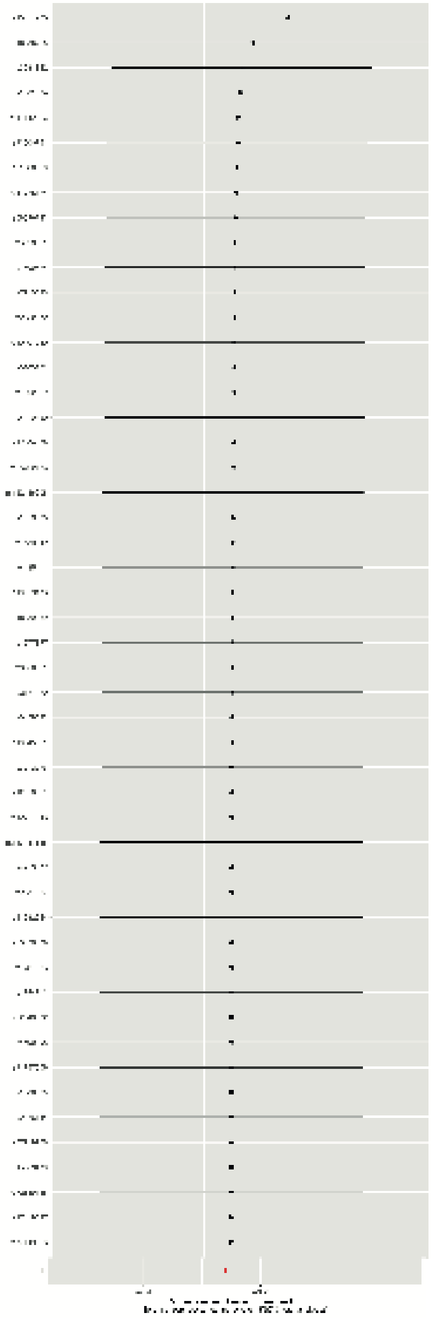

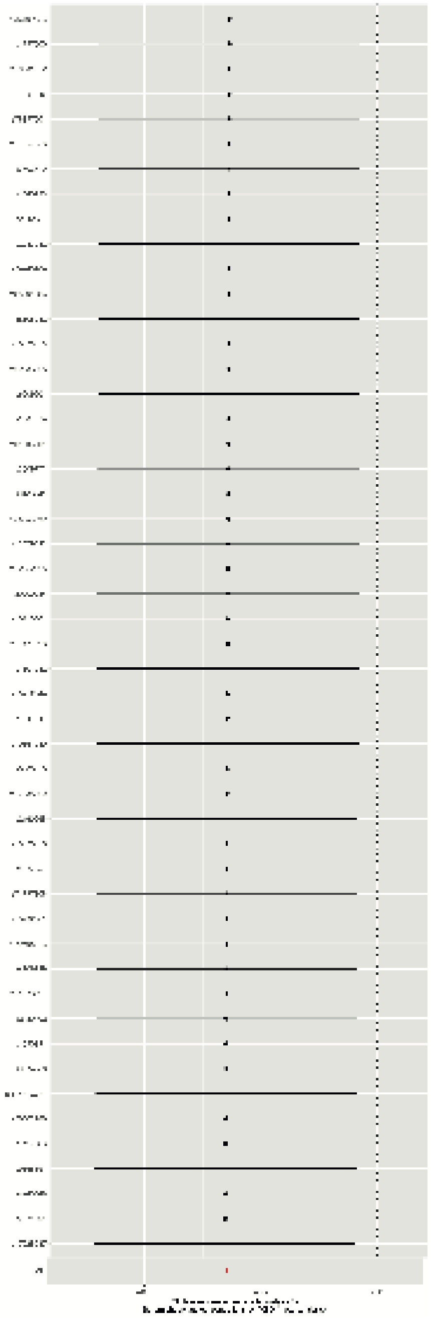

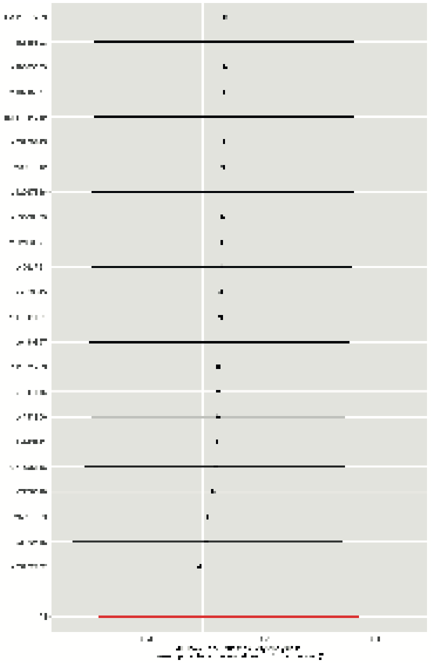

Supplementary Figure 11. Mendelian randomisation leave-one-out analyses for the effects of serum phosphate concentrations on risk of kidney stone disease in FinnGen.

The x-axis reflects the log-odds ratio for kidney stone disease. All= overall inverse-variance weighted Mendelian randomisation estimate; KSD= kidney stone disease; MR= Mendelian randomisation
