# Supplementary figures and images for: Central Adiposity Increases Risk of Kidney Stone Disease through Effects on Serum Calcium Concentrations
Source: J Am Soc Nephrol. 2023 Oct 3;34(12):1991–2011. doi: 10.1681/ASN.0000000000000238 (PMC10703081; doi:10.1681/ASN.0000000000000238)

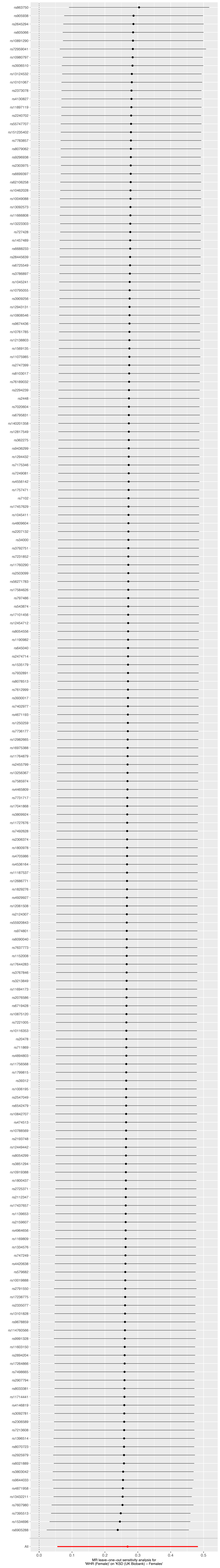

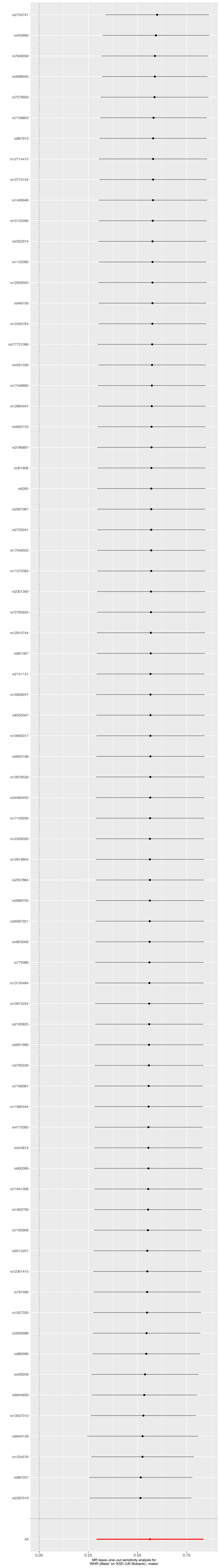

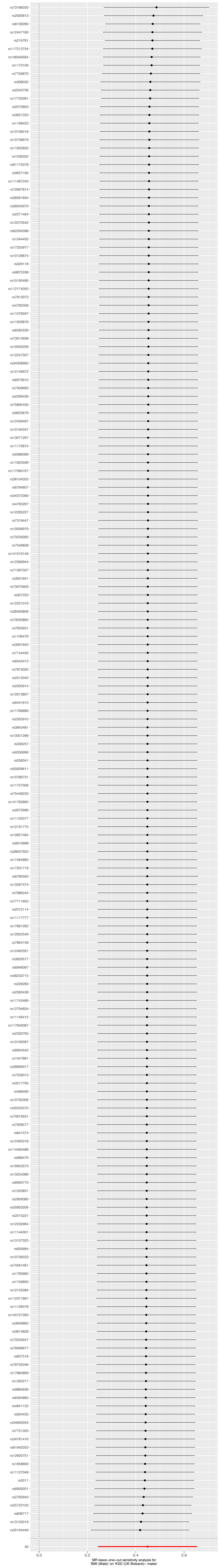

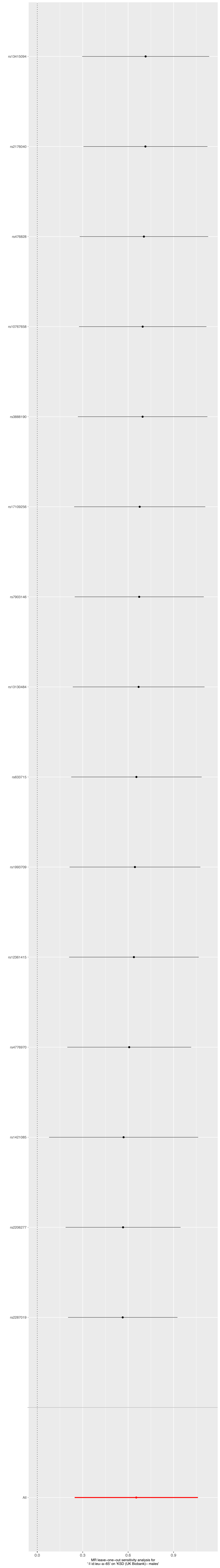

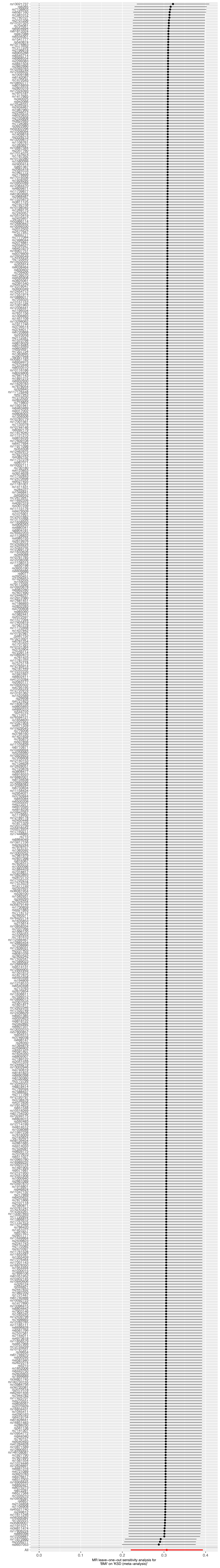

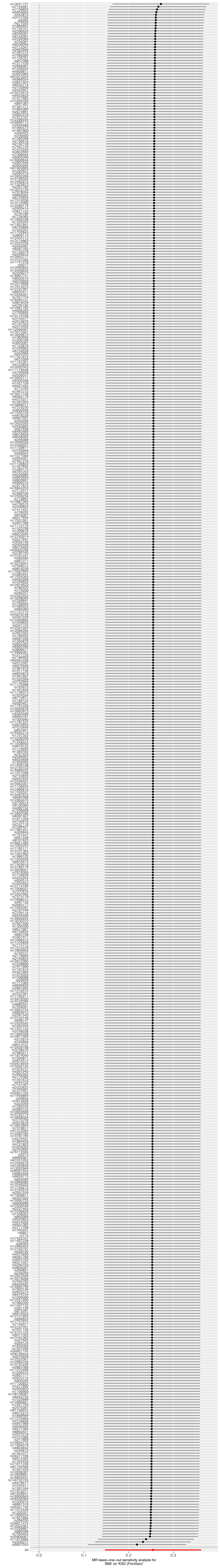

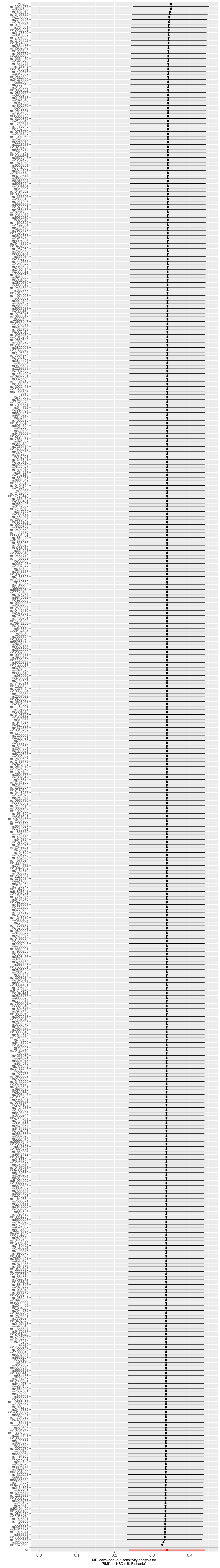

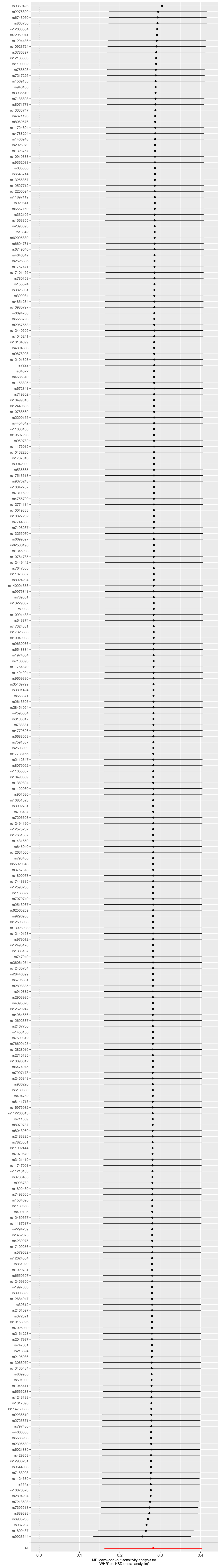

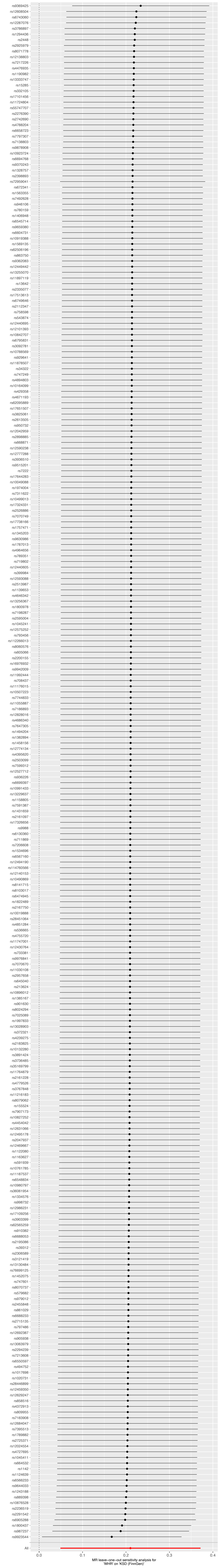

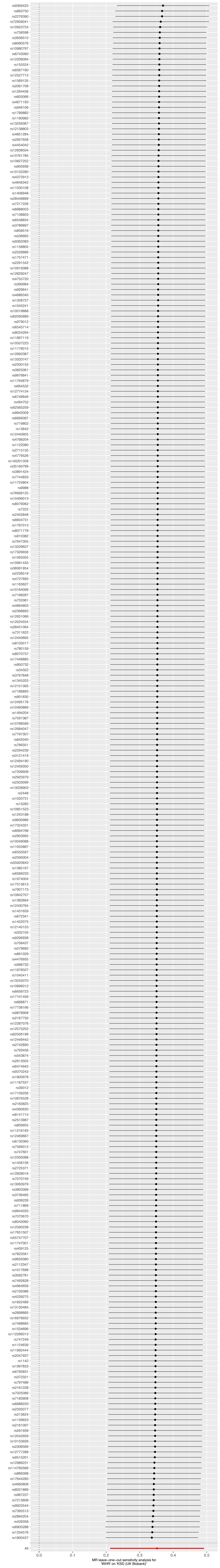

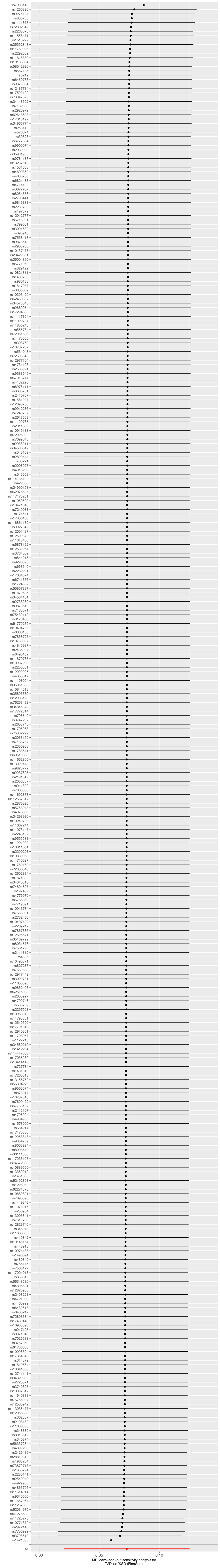

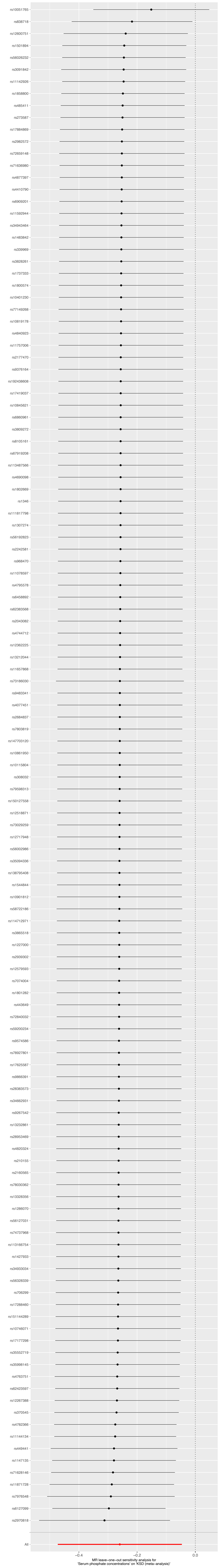

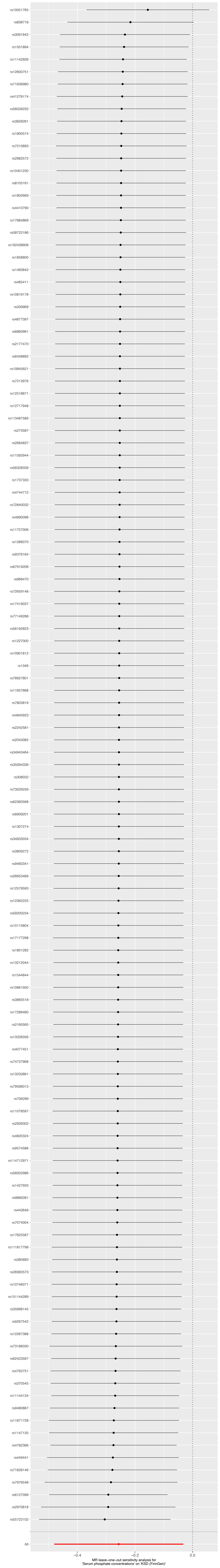

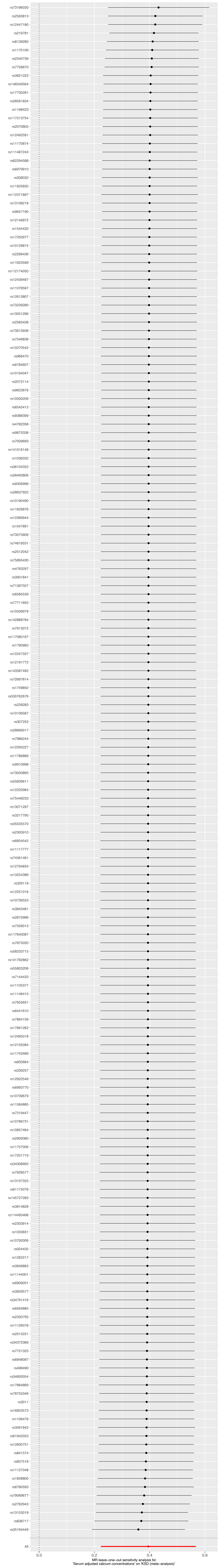

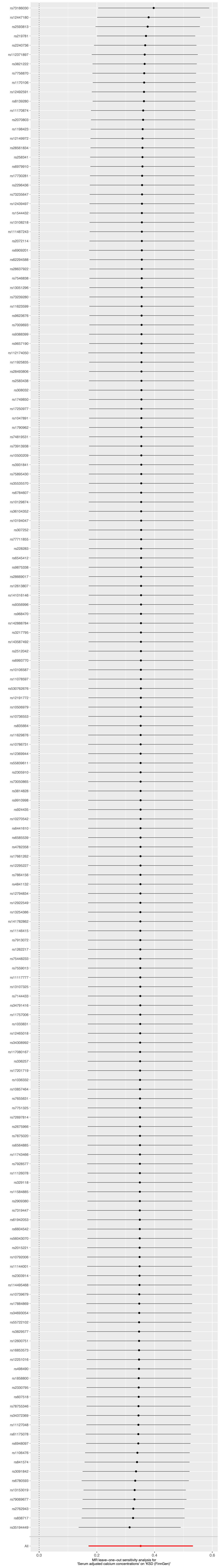

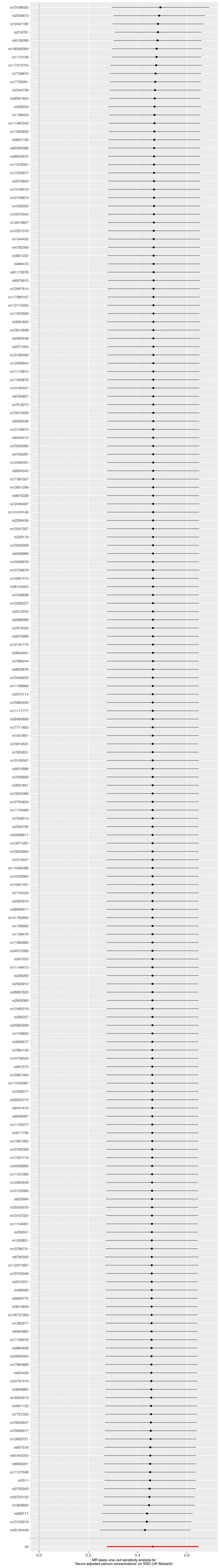

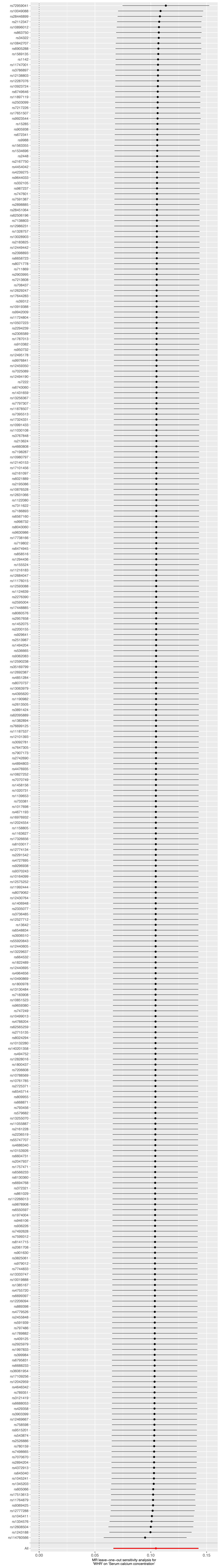

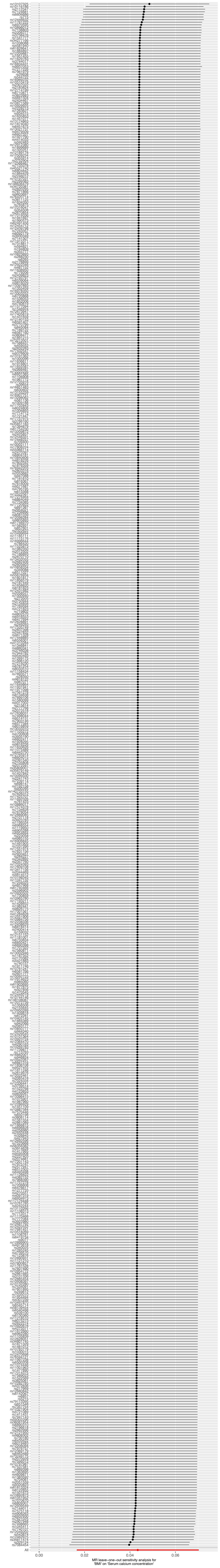

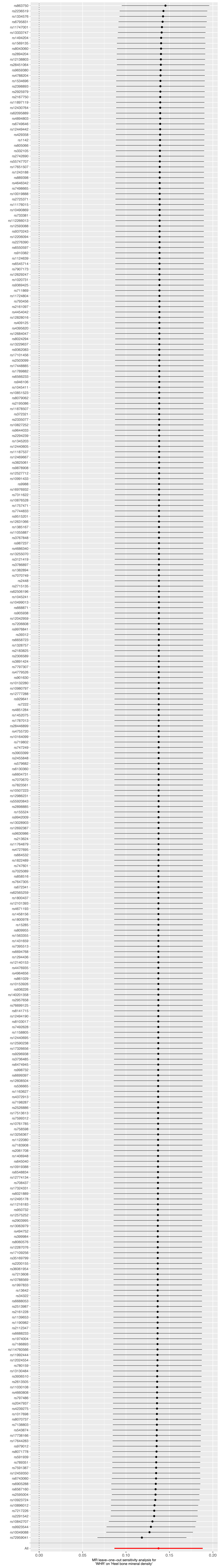

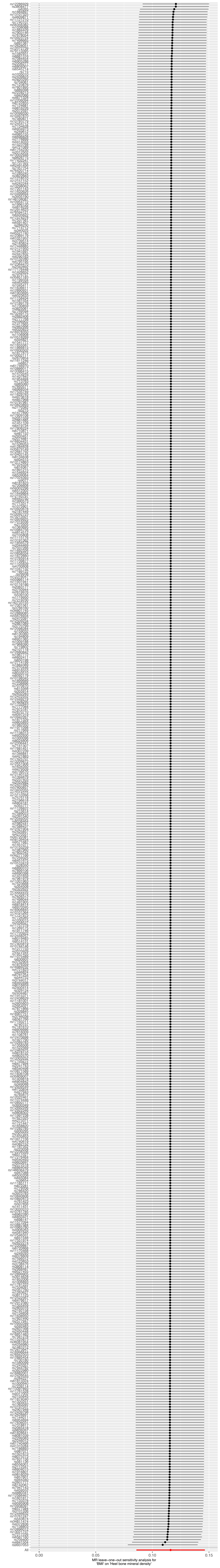

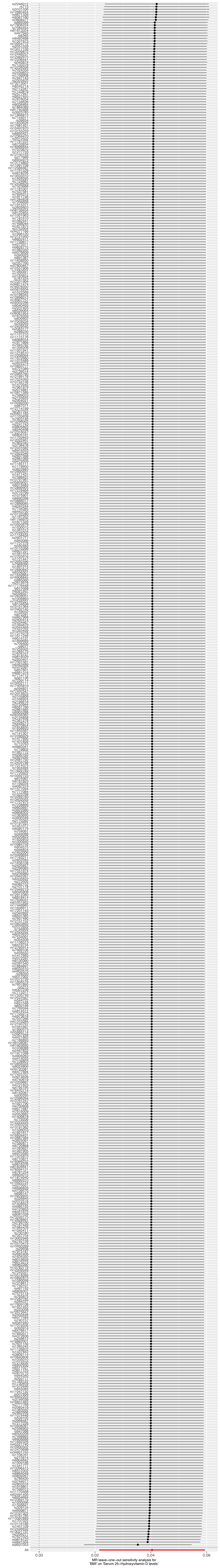

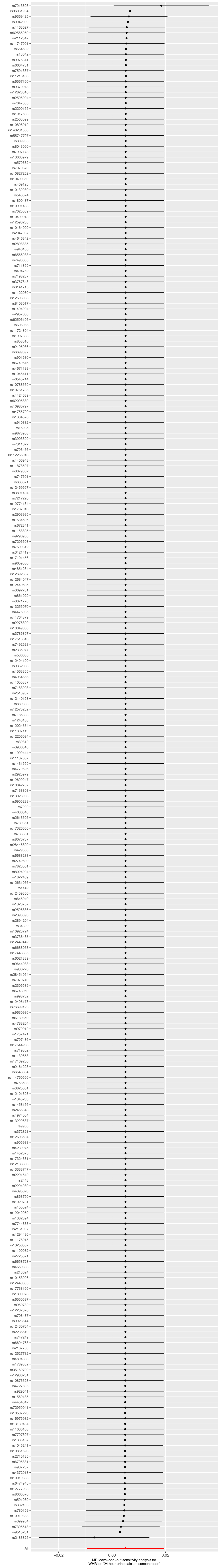

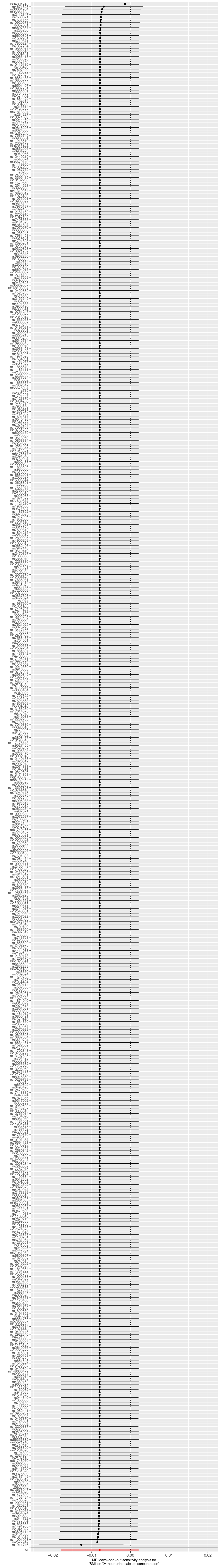

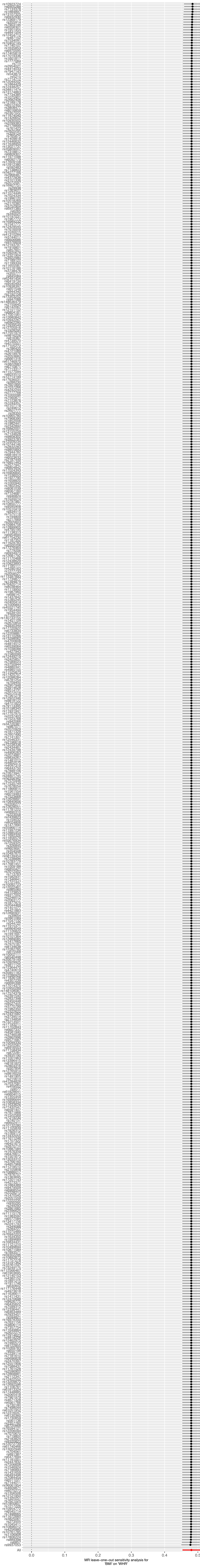

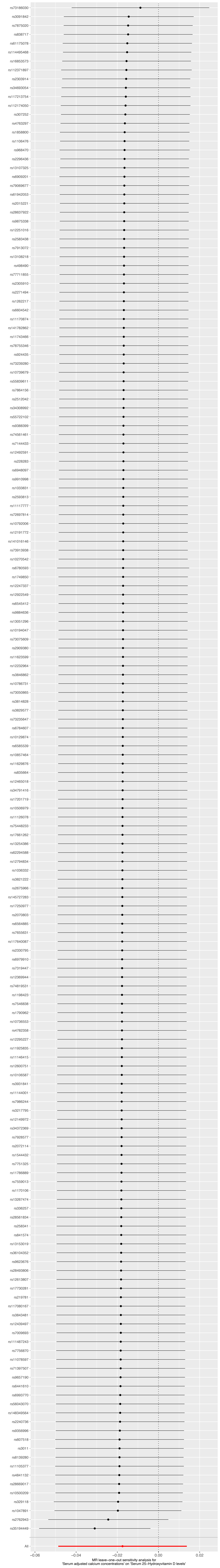

Supplement: Supplementary file 2 [file jasn-34-1991-s002.pdf]
